# Investigating how cancer-related symptoms influence work outcomes among cancer survivors: a systematic review

**DOI:** 10.1007/s11764-021-01097-5

**Published:** 2021-08-23

**Authors:** Chia Jie Tan, Samantha Yin Ching Yip, Raymond Javan Chan, Lita Chew, Alexandre Chan

**Affiliations:** 1 Department of Pharmacy, National University of Singapore, Singapore, Singapore; 2 Caring Futures Institute, College of Nursing and Health Sciences, Flinders University, Adelaide, South Australia Australia; 3 Department of Pharmacy, National Cancer Centre Singapore, Singapore, Singapore; 4 Department of Clinical Pharmacy Practice, School of Pharmacy & Pharmaceutical Sciences, University of California, 101 Theory, Suite 100, Irvine, CA 92612 USA

**Keywords:** Employment rate, Return to work, Absenteeism, Presenteeism, Work productivity, Symptom burden

## Abstract

**Purpose:**

The purpose of this study is to investigate how different cancer-related symptoms influence work outcomes among cancer survivors.

**Methods:**

A literature search was performed in PubMed, EMBASE, CINAHL, PsycINFO, and Scopus to identify studies published between 1st January 1999 and 30th October 2020 that investigated the impact of specific cancer-related symptoms on work outcomes among cancer survivors who have completed primary antineoplastic treatment. Study findings were extracted and grouped by symptoms and work outcomes, allowing comparison of associations between these outcomes.

**Results:**

Seventy-three articles representing 68 studies were eligible for inclusion. From these studies, 27 cancer-related symptoms, 9 work outcomes, and 68 unique associations between specific symptoms and work outcomes were identified. Work status (return to work and employment rates) was most commonly studied, and symptom burden was mainly measured from the patient’s perspective. Higher symptom burden was generally associated with trends of poorer work outcomes. Significant associations were reported in most studies evaluating body image issues and work status, oral dysfunction and work status, fatigue and work ability, and depression and work ability.

**Conclusion:**

Several cancer-related symptoms were consistently associated with inferior work outcomes among cancer survivors. Body image issues and oral dysfunction were shown to be associated with poorer employment rates, while fatigue and depression were linked to lower levels of work performance.

**Implications for Cancer Survivors:**

Failure to return to work and decreased productivity post-cancer treatment can have negative consequences for cancer survivors and society at large. Findings from this review will guide the development of work rehabilitation programs for cancer survivors.

**Protocol registration:**

PROSPERO identifier CRD42020187754

**Supplementary Information:**

The online version contains supplementary material available at 10.1007/s11764-021-01097-5.

## Introduction

With improved long-term survival rates of cancer, work and employment have emerged as increasingly prominent issues among cancer survivors. Across a range of various cancers, approximately 40% of cancer survivors do not return to work after completion of treatment [1]. Cancer survivors who remain employed are also more likely to miss work, reduce working hours, or report limitations at work compared to their non-cancer counterparts [1, 2]. Furthermore, cancer survivors have been reported to be less productive in unpaid components of work, such as homemaking and volunteering [3]. The ability of cancer survivors to resume normal levels of productivity is crucial for both survivors and society at large. From a societal perspective, inferior work outcomes among cancer survivors lead to productivity loss, which was estimated to cost US$3593 per capita annually in the USA [4].

Among cancer survivors, inferior work outcomes post-treatment result from a mismatch between an individual’s functional capabilities and work demands [5]. We speculate that cancer survivors often suffer from impaired functional capabilities due to lingering symptoms from cancer and antineoplastic treatment. This is supported by empirical evidence from published studies that have reported that cancer survivors continue to face mental and physical difficulties at work and were more likely to quit due to cancer-related disabilities [6, 7]. These issues could be addressed by rehabilitative care, which has been shown to facilitate return-to-work (RTW) and reduce early retirement among cancer survivors [8, 9].

Rehabilitative care encompasses a wide range of services that aim to mitigate symptom burden and functional impairments among cancer survivors. While rehabilitative care is specific to the individual needs of patients, survivorship services targeting cancer-related symptoms that strongly impact work outcomes should be prioritized when developing work rehabilitation programs, especially in resource-constrained healthcare settings. A comprehensive understanding and comparison of how specific cancer-related symptoms influence work outcomes is therefore crucial. Despite the abundance of observational studies that have examined the relationship between specific cancer-related symptoms and work outcomes, most systematic reviews have not focused on symptom burden [10–12] or did not identify the impact of specific symptoms [13, 14].

This systematic review, therefore, aims to describe and compare how different cancer-related symptoms affect work outcomes among cancer survivors based on findings reported in the primary literature, allowing the identification of symptoms that are closely linked to poor work outcomes. Besides guiding the prioritization of survivorship services for work rehabilitation programs, the findings of this review will also provide insights into the current state of research on the relationship between symptom burden and work outcomes, identifying gaps in the field that need to be addressed.

## Methods

The protocol for this systematic review has been registered on PROSPERO (ID: CRD42020187754) and reporting of the review is in accordance with the Preferred Reporting Items for Systematic Reviews and Meta-Analyses (PRISMA) guidelines [15].

### Search strategy

Literature search was performed in the PubMed, EMBASE, CINAHL, PsycINFO, and Scopus databases for studies published from 1st January 1999 to 30th October 2020 with the final search performed prior to data analysis. Initial searches were conducted using a combination of MeSH terms and free-text terms related to cancer survivors and work-related outcomes separately, with each term combined with “or.” Subsequently, the results of both searches were collectively combined with “and.” The search syntax was then adopted per database (Supplementary Material 1).

### Eligibility criteria

Studies that quantified the impact of symptom burden on work outcomes among adult cancer survivors who had completed primary treatment (surgery, cytotoxic chemotherapy, and/or radiotherapy) were eligible. If the treatment status of study participants was not specified, work outcomes should have been evaluated at least 12 months from diagnosis, when the primary treatment for most cancer diagnoses would have been completed. Studies were excluded if they included survivors of childhood cancer or if patients were undergoing palliative treatment during the assessment of work outcomes.

Eligible studies also had to identify a specific symptom or symptoms localized in a specific body region as the aim of this review was to provide insights on specific types of services that should be prioritized in work rehabilitation programs. Therefore, studies that only evaluated the exposure as a collation of symptoms (for example, a summation score from a quality-of-life questionnaire covering various symptoms) were not eligible. Furthermore, studies were excluded if work was measured as a part of a composite outcome that included non-work components such as schooling.

Studies that examined the effectiveness of interventions in alleviating symptom burden were also excluded as the findings would not have reflected the actual impact of symptoms on work outcomes. Other exclusion criteria were qualitative studies, systematic and narrative reviews, books, commentaries, editorials, magazine articles, and conference proceedings or abstracts. Only articles published in English with full texts available were reviewed.

### Study selection

After the removal of duplicates, search results were first screened based on the title and abstract. Full texts of potentially relevant articles were subsequently retrieved and reviewed for eligibility. The screening process was carried out by CJT and SYCY independently with discrepancies reconciled by discussion or adjudication by a third study team member AC. Articles that were included in the review were scrutinized based on the reported methodology and demographics to determine if findings were reported from the same studies.

### Data extraction and synthesis

Data was extracted from all eligible studies into a piloted spreadsheet by CJT and independently reviewed by SYCY. Extracted variables included study characteristics (author, publication year, country of origin, and study design), population characteristics (sample size, age, cancer type, staging, and other clinical variables), the tools and analytical methods used for measuring symptom burden (presence, severity, and/or frequency) and work outcomes, and the association between symptom burden and work outcomes. Work outcomes were grouped into several categories, which are work status (current employment, RTW, early retirement, and disability), absenteeism, presenteeism (at-work productivity, self-perceived work ability), unpaid work, and other outcomes. Study findings were organized by the type of symptoms and work outcomes evaluated and findings from different articles reporting on the same study were extracted to the same spreadsheet entry. All studies were included in the qualitative synthesis. Meta-analyses and assessment of publication bias were not carried out as a large number of studies did not report effect measures, especially when assessed symptoms were not statistically significant or excluded from the final model presented. Vote counting was performed where the proportion of studies yielding significant findings for each unique association was calculated. The proportions were compared between each unique association.

### Quality assessment

The quality of studies and risk of bias were assessed using the Newcastle–Ottawa quality assessment scale (NOS). NOS evaluates studies based on three main aspects—the selection of the study groups, comparability of the groups, and the ascertainment of the exposure and outcome of interest. Assessment was carried out independently by CJ and SYCY and any discrepancies were discussed until a consensus was reached or the opinion of a third study team member AC was sought.

## Results

**Fig. 1:**
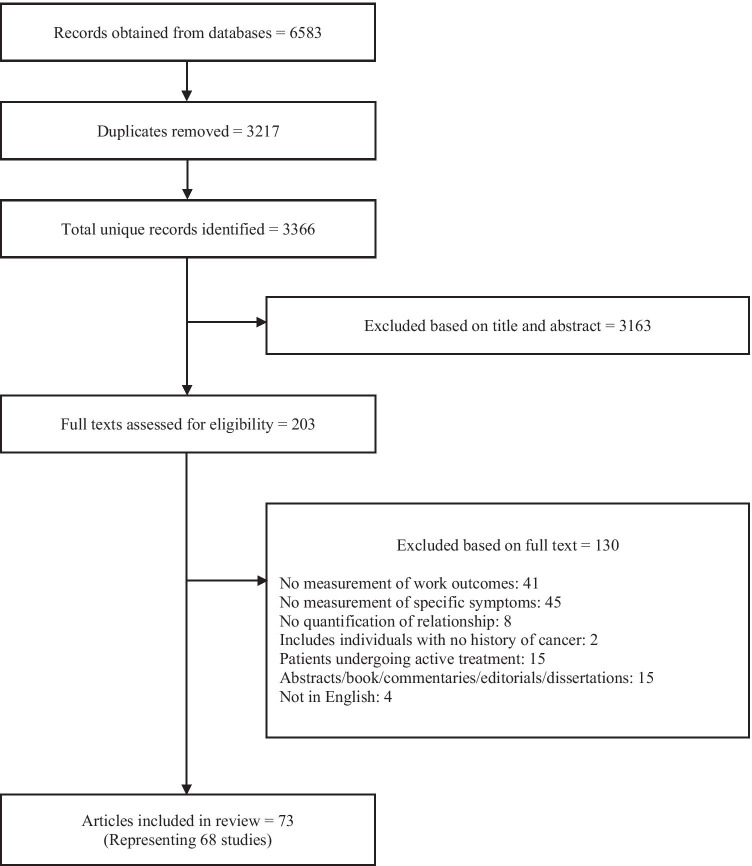
Literature search and study screening flow chart

The literature search identified a total of 6583 articles, of which 3217 were duplicates, yielding 3366 articles for assessment of eligibility. Another 3163 articles were excluded based on the title and abstract while 130 were removed during full-text screening. Finally, 73 articles, reporting findings from 68 studies, were selected for review (Fig. 1).

### Study and participant characteristics

Collectively, the studies represented 38,603 participants from 24 countries, mostly from Western Europe (35.3%) and North America (27.9%) with the vast majority being high-income nations (89.7%). Forty-two (61.8%) studies were cross-sectional in nature while the remaining 26 (38.2%) were cohort studies. Sample sizes ranged from 22 to 5306, with 11 (16.0%) studies consisting of more than 1000 participants.

The studies included a wide range of cancer types, with 25 (36.8%) consisting of only breast cancer survivors and 17 (25.0%) with patients diagnosed with various cancers. Among studies that reported a summary estimate of the age of participants, slightly more than half (55.9%) reported a mean or median higher than 50 years old. Forty-six studies (67.6%) included an upper age limit as part of the eligibility criteria, which is usually indicative of the retirement age in the region. Fifteen studies (22.1%) comprised of patients with advanced and late-stage disease, among which 5 included patients who had active disease and were still living with cancer. Among 40 studies that reported the time after treatment completion, work outcomes were evaluated at least 1 year after treatment in most studies (60%). The characteristics of the included studies are summarized in Table 1 and listed in Supplementary Material 2.

**Table 1:** Characteristics of studies reviewed

Characteristics	*N* (%)
Region^1,2^	
North America	19 (27.9)
South America	4 (5.9)
West Europe	24 (35.3)
Scandinavia	8 (11.8)
East Europe	1 (1.5)
Middle East	1 (1.5)
South Asia	1 (1.5)
East Asia	8 (11.8)
Southeast Asia	1 (1.5)
Oceania	2 (2.9)
Country income level^1,3^	
High-income	61 (89.7)
Upper middle-income	2 (2.9)
Lower middle-income	6 (8.8)
Study design	
Cross-sectional	42 (61.8)
Prospective cohort	23 (33.8)
Retrospective cohort	3 (4.4)
Number of participants	
< 100	14 (21)
100 to 500	34 (50)
501 to 1000	9 (13)
> 1000	11 (16)
Age estimate	
Reported as mean/median	
> 50 years old	31 (45.6)
< 50 years old	22 (31.4)
Reported as proportions	
Predominantly > 50 years old	7 (10.3)
Predominantly < 50 years old	5 (7.4)
Unreported	3 (4.4)
Cancer types	
Breast	25 (36.8)
Head and neck	7 (10.3)
Hematological	6 (8.8)
Gynecological	4 (5.9)
Lung	2 (2.9)
Prostate	2 (2.9)
Brain	2 (2.9)
Colorectal	1 (1.5)
Testicular	1 (1.5)
Thyroid	1 (1.5)
Various	17 (25.0)
Disease stage	
Included all stages^4^	15 (22.1)
Excluded advanced disease	22 (32.4)
Unreported	31 (45.6)

^1^Sum of percentages exceed 100% as one study was conducted in USA (North America, high-income) and Peru (South America, upper middle-income)

^2^Regions based on United Nations Geoscheme and cultural background

^3^Income level based on World Bank classification of countries by GNI per capita

^4^Five studies included patients with active disease; 7 excluded while 3 did not report

### Quality of study design and analysis

Based on the Newcastle–Ottawa quality assessment scale, most studies were considered to be at low risk of selection bias, with study cohorts that were truly or somewhat representative of the population of interest and controls that were derived from the same community as the participants with significant symptom burden. Confounding by baseline work status or performance was addressed in more than half of the studies (57.4%) by, for example, excluding participants who were not working prior to cancer diagnosis or the use of outcome measures that compared the participants’ current status to pre-cancer levels of work productivity or ability. Most studies (63.2%) adjusted for other potential confounders such as age, occupation type, and time since diagnosis. For cohort studies, the follow-up period was generally adequate (80.8% of studies), but the attrition rate was > 20% for half of them. A critical issue that was considered unsatisfactory for a large majority of the studies was the measurement of exposure and outcome, which was largely conducted with self-reporting tools. The quality assessment of all the studies reviewed is presented in Supplementary Material 3.

### Measurement of symptom burden

A total of 27 symptoms were identified from the studies reviewed (Supplementary Material 4 and 5). Fatigue was most widely reported (55.9%), followed by depression (50%), cognitive impairment (39.7%), and pain (38.2%). Several symptoms were only investigated in 1 or 2 studies, such as seizures, weight gain, weight loss, drowsiness, hot flashes, rash, sexual dysfunction, and urinary problems. Patient-reported outcomes (PRO) were commonly used in the assessment of symptom burden with validated PRO instruments found in 51 (75.0%) studies while non-validated tools, such as using a single item to inquire the presence of symptoms and visual analog scales, were featured in 14 (20.6%) studies (Table 2). In some studies, although validated instruments were used, the tools were originally developed for the measurement of health-related quality of life in general, and only selected items or domains were relevant to and used for the evaluation of specific symptoms. Clinician-reported outcome measures (16.2%) were also employed, predominantly in the identification of seizures, hot flashes, and lymphedema while performance outcomes, namely, neuropsychological testing and audiometry, were used in the measurement of cognitive impairment and hearing problems, respectively.

**Table 2:** Measurement of symptom burden in the studies reviewed

Symptom	Number of studies with outcomes, *N* (%)				Total	Examples of patient-reported outcome tools
	Performance outcomes	Clinician-reported	Patient-reported			
			Non-validated	Validated		
Oral dysfunction	0 (0%)	0 (0%)	0 (0%)	5 (100%)	5	UW-QoL MDASI-HN EORTC-QLQ-H&N35
Breast issues	0 (0%)	0 (0%)	0 (0%)	4 (100%)	4	EORTC-QLQ-B23
Upper limb problems	0 (0%)	4 (28.6%)	4 (28.6%)	7 (50%)	14	EORTC-QLQ-B23
Lower limb problems	0 (0%)	1 (33.3%)	1 (33.3%)	1 (33.3%)	3	EORTC-QLQ-CX24
Cognitive impairment	2 (7.4%)	1 (3.7%)	4 (14.8%)	21 (77.8%)	27	EORTC-QLQ-C30 CFQ CSC-W FACT-Cog AFI
Neuropathy	0 (0%)	0 (0%)	0 (0%)	4 (100%)	4	PAOFI SCIN FACT-GOG
Pain	0 (0%)	0 (0%)	7 (26.9%)	19 (73.1%)	26	EORTC-QLQ-C30 MDASI-ECOG MDASI-HN BPI
Seizures	0 (0%)	2 (100%)	0 (0%)	0 (0%)	2	
Sensory issues	1 (16.7%)	0 (0%)	1 (16.7%)	5 (83.3%)	6	EORTC-QLQ-H&N35 MDASI-HN
Anorexia	0 (0%)	0 (0%)	0 (0%)	9 (100%)	9	EORTC-QLQ-C30 MDASI-ECOG MDASI-HN UW-QoL
Weight gain	0 (0%)	0 (0%)	0 (0%)	1 (100%)	1	EORTC-QLQ-H&N35
Weight loss	0 (0%)	0 (0%)	0 (0%)	1 (100%)	1	EORTC-QLQ-H&N35
Nausea/vomiting	0 (0%)	0 (0%)	1 (9.1%)	10 (90.9%)	11	EORTC-QLQ-C30 MDASI-ECOG
Diarrhea/↑bowel urgency	0 (0%)	0 (0%)	0 (0%)	9 (100%)	9	EORTC-QLQ-C30 MDASI-ECOG EPIC
Constipation	0 (0%)	0 (0%)	0 (0%)	9 (100%)	9	EORTC-QLQ-C30 MDASI-ECOG
Anxiety	0 (0%)	0 (0%)	2 (9.5%)	19 (90.5%)	21	UW-QoL HADS POMS
Depression	0 (0%)	1 (2.9%)	2 (5.9%)	31 (91.2%)	34	UW-QoL HADS CES-D BDI PHQ-9
Body image issues	0 (0%)	0 (0%)	2 (33.3%)	4 (66.7%)	6	UW-QoL EORTC-QLQ-B23 MDASI-ECOG
Coughing	0 (0%)	0 (0%)	0 (0%)	5 (100%)	5	EORTC-QLQ-H&N35 MDASI-HN LCSS
Dyspnea	0 (0%)	0 (0%)	0 (0%)	10 (100%)	10	EORTC-QLQ-C30 MDASI-HN LCSS
Fatigue	0 (0%)	2 (5.3%)	4 (10.5%)	33 (86.8%)	38	BFI EORTC-QLQ-C30 FQ FAQ FACIT-F MFI
Insomnia	0 (0%)	0 (0%)	1 (7.7%)	12 (92.3%)	13	EORTC-QLQ-C30 Pittsburgh Sleep Quality Index
Drowsiness	0 (0%)	0 (0%)	0 (0%)	2 (100%)	2	MDASI-ECOG MDASI-HN
Hot flashes	0 (0%)	1 (100%)	0 (0%)	0 (0%)	1	
Rash	0 (0%)	0 (0%)	0 (0%)	2 (100%)	2	MDASI-ECOG
Sexual dysfunction	0 (0%)	0 (0%)	0 (0%)	2 (100%)	2	EORTC-QLQ-BR23
Urinary problems	0 (0%)	1 (33.3%)	0 (0%)	2 (66.7%)	3	EPIC
Total	2 (2.9%)	11 (16.2%)	14 (20.6%)	51 (75.0%)	68	

Abbreviations: *AFI*, Attentional Fatigue Inventory; *BDI*, Beck Depression Inventory; *BFI*, Brief Fatigue Inventory; *BPI*, Brief Pain Scale; *CES-D*, Center for Epidemiologic Studies Depression Scale; *CFQ*, Cognitive Failures Questionnaire; *CSC-W*, Cognitive Symptom Checklist-Work; *EORTC-QLQ-C30*, EORTC-QLQ-Core 30; *EORTC-QLQ-B23*, EORTC-QLQ-Breast 23; *EORTC-QLQ-H&N35*, EORTC-QLQ-Head and Neck 35; *EORTC-QLQ-CX24*, EORTC-QLQ-Cervical 24; *EPIC*, Expanded Prostate Cancer Index Composite; *FQ*, Fatigue Questionnaire; *FAQ*, Fatigue Assessment Questionnaire; *FACIT-F*, Functional Assessment of Chronic Illness-Fatigue; *FACT-Cog*, Functional Assessment of Cancer Therapy–Cognitive; *FACT-GOG*, Functional Assessment of Cancer Therapy Gynaecological Oncology Group; *HADS*, Hospital Anxiety and Depression Scale; *MDASI-ECOG*, MD Anderson Symptom Inventory-Modified; *MDASI-HN*, MD Anderson Symptom Inventory-Head and Neck; *MFI*, Multidimensional Fatigue Inventory; *PAOFI*, Patient Assessment of Own Functioning Inventory; *PHQ-9*, Patient Health Questionnaire-9; *POMS*, Profile of Mood States; *SCIN*, Scale for Chemotherapy-Induced Long-Term Neurotoxicity; *UW-QoL*, University of Washington Quality of Life Questionnaire

### Evaluation of work outcomes

Work outcomes reported in more than half of the studies were related to current employment status or RTW (70.6%), followed by work ability (14.7%) and work productivity (13.2%). Other work outcomes that were measured included absenteeism, decrease in income level or working hours, changes in work scope, and occupational role limitations (Supplementary Material 4 and 5). Unpaid work was not measured in any of the eligible studies. Similar to the measurement of symptom burden, work outcomes were overwhelmingly reported from the patient’s perspective but mostly with instruments with no description of prior validation (77.9%) (Table 3). The exception to this was the evaluation of work ability and work productivity, which were almost entirely measured using the Work Ability Index and the Work Limitations Questionnaire, respectively. Both questionnaires have been validated and extensively used in the cancer population. In 2 studies, work outcomes were determined by clinicians, who assessed work disability status or determined if work restrictions could be lifted based on institution-specified criteria while in 1 study, employment status was directly extracted from an occupational health database containing data reported from employers.

**Table 3:** Measurement of work outcomes in the studies reviewed

Work outcome	Number of studies with outcomes, *N* (%)				Total	Examples of patient-reported outcome tools
	Employer-reported	Clinician-reported	Patient-reported			
			Non-validated	Validated		
Absenteeism	0 (0%)	0 (0%)	1 (100%)	0 (0%)	1	
Employment/RTW	1 (2.1%)	1 (2.1%)	45 (93.8%)	1 (2.1%)	48	
Early retirement/disability	0 (0%)	1 (20%)	4 (80%)	0 (0%)	5	
Work ability	0 (0%)	0 (0%)	1 (10%)	9 (90%)	10	WAI FACT/GOG
Work productivity	0 (0%)	0 (0%)	1 (11.1%)	8 (88.9%)	9	WLQ
Occupational role limitations	0 (0%)	0 (0%)	0 (0%)	1 (100%)	1	Occupational Role Questionnaire
Changes in work	0 (0%)	0 (0%)	3 (100%)	0 (0%)	3	
Income	0 (0%)	0 (0%)	1 (100%)	0 (0%)	1	
Working hours	0 (0%)	0 (0%)	1 (100%)	0 (0%)	1	
Total	1 (1.5%)	2 (2.9%)	53 (77.9%)	18 (26.5%)	68	

Abbreviations: *FACT/GOG*, Functional Assessment of Cancer Therapy Gynaecological Oncology Group; *WAI*, Work Ability Index; *WLQ*, Work Limitations Questionnaire

### Association of symptom burden with work outcomes

A total of 68 unique associations between symptoms and work outcomes were identified from the eligible studies (Table 4). A higher symptom burden was generally associated with trends of poorer work outcomes. However, most associations were not found to be statistically significant, and many studies (35.3%) did not report all effect measures, especially if the association was not statistically significant or the symptom was not included in the final analytical model. Additionally, some studies (11.8%) did not report measures of data dispersion.

**Table 4:**
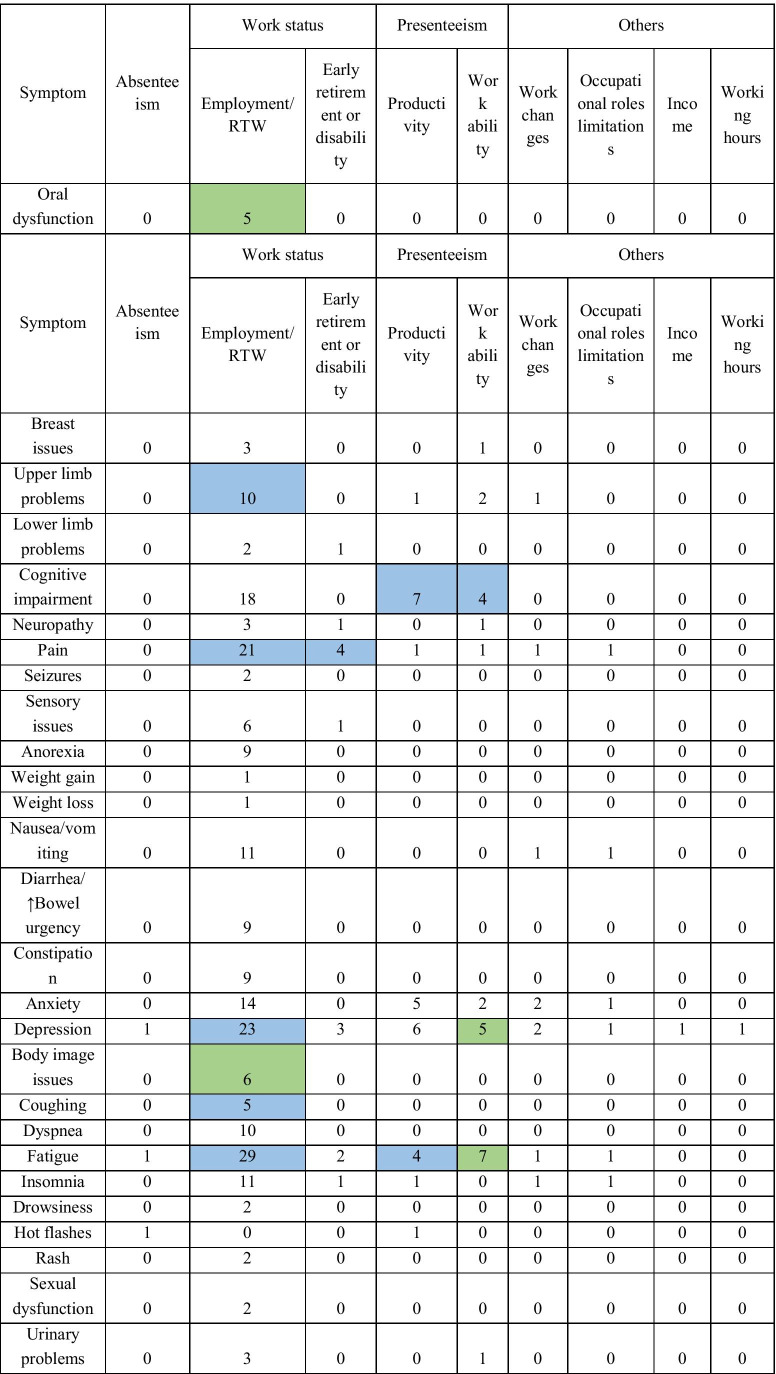
Number of studies evaluating association between specific symptoms and work outcomes

Cells shaded in green indicate that more than half of the studies evaluating the association reported significant findings (excluding associations investigated by 2 or less studies) while cells shaded in blue indicate that more than one-third of the studies evaluating the association reported significant findings (excluding associations investigated by 2 or less studies)

The association between work status (employment status/RTW or early retirement/work disability) and the following symptoms was examined in more than 10 studies: fatigue, depression, pain, cognitive function, anxiety, nausea/vomiting, and insomnia. However, significant findings were reported in less than half of the studies evaluating the association between work status and these symptoms. Among the remaining associations between symptoms and work status, statistically significant findings were demonstrated in the majority of the studies assessing employment/RTW and oral dysfunction (5 of 5 studies) and employment/RTW and body image issues (4 of 6 studies). However, it should be noted that several dimensions of oral dysfunction were measured with no adjustment for multiple testing.

With regards to work ability and work productivity, none of the associations between work outcomes and symptom burden was investigated in more than 10 studies. Significant findings were demonstrated in more than half of the studies evaluating the association between work ability and fatigue (4 of 7 studies) and work ability and depression (3 of 5 studies). For absenteeism and other work outcomes, not more than 2 studies evaluated the relationship between each work outcome and specific symptoms. No significant associations were detected for studies evaluating absenteeism, income, and working hours.

Symptoms with notable findings in the review are summarized below. A complete description of findings for each association between symptom and work outcome identified from the literature can be found in the Supplementary Material 6.

#### Fatigue and insomnia

The impact of fatigue on a range of work outcomes was evaluated in 38 (55.9%) studies, in which fatigue was predominantly assessed with validated PRO measures. The studies included survivors of various cancers, mostly previously diagnosed with breast cancer. More than half of the studies that identified work ability as an outcome found a significantly negative association between symptom severity and work outcome [16–19] while only one-third of the studies that investigated work status yielded significant results [20–30]. None of the studies evaluating insomnia showed significant findings.

#### Anxiety and depression

Anxiety and depression were evaluated in approximately 21 (30.9%) and 34 (50.0%) studies, respectively, and were often investigated in the same studies due to the use of PRO tools that measure both constructs. The most common outcome of interest was employment/RTW but work ability, productivity, and other work outcomes were also examined. Similar to fatigue, approximately one-third of the studies evaluating depression and work status demonstrated significant findings [18, 20, 21, 27, 31–37] but more than half of the studies evaluating work ability yielded significantly negative associations between depression and work ability [16–18]. In contrast, studies consistently shown non-significant findings when the association of anxiety and various work outcomes was evaluated.

#### Pain

Studies evaluating the impact of pain on work outcomes largely focused on the work status of participants, evaluating employment status/RTW or early retirement/disability. Notably, PROs used to measure the presence or severity of pain in a quarter of the studies were not validated tools [22, 31, 38–40]. Studies with more than 1000 participants consistently showed that patients with more severe pain were less likely to return to work or be employed [28, 40–43]. Studies investigating other work outcomes mostly did not detect any significant relationship [44–46].

#### Cognitive impairment

Work outcomes that were examined in association with cognitive impairment included employment status, work productivity, and work ability. Most studies evaluating work status reported effect measures that were not statistically significant [20, 22, 23, 26, 33, 36, 47–53] or of small magnitude [29, 37, 54]. An exception to this was Dieluweit et al., where survivors of various cancers with cognitive impairment had half the odds of being employed more than 5 years after diagnosis compared to those without [55]. In contrast, several studies consistently demonstrated that poorer cognitive function was correlated to lower levels of work ability or work productivity, predominantly among breast cancer survivors [17, 56–58]. Specific domains of cognitive function were also investigated in a number of studies, such as attention, executive function, memory, and verbal fluency, but no consistent trends could be observed. One study that employed performance outcomes (neuropsychological testing) as an indicator of cognitive function reported no significant impact of cognitive impairment on employment status and work ability [50].

#### Nausea and vomiting

The association between nausea and vomiting and work status was assessed in 11 (16.2%) of the studies reviewed. Although the target population was no longer on active antineoplastic treatment and hence not expected to be affected by nausea and vomiting, 3 studies demonstrated significant findings [20, 37, 46]. For example, Steiner et al. reported that up to 46% and 55% of survivors who experienced changes at work or had limitations in occupational roles complaint of nausea and vomiting [46]. Interestingly, in this study, approximately 20% or participants with no work issues cited nausea and vomiting as a concern as well [46].

#### Oral dysfunction

Five (7.4%) studies investigated the association of employment status with different aspects of oral health, including dry mouth, sticky saliva, teeth problems, and functional issues (speaking, swallowing, and chewing), mostly among head and neck cancer survivors. The dimensions examined depended on the PRO tool that was used. Significant associations were observed in all studies, mainly with dry mouth, sticky saliva, and chewing function [23, 37, 48, 53, 59]. Patients who reported a higher level of symptom burden in these domains had lower odds of employment.

#### Body image issues

A few studies evaluated the impact of body image issues on employment and RTW using PRO instruments. Half of the studies demonstrated significant associations [29, 47, 59]. The largest effect size was reported by Chen et al. where cancer survivors with scarring had 3 times the odds of being denied a job due to their medical history compared to those without [59].

## Discussion

This systematic review summarized the association of cancer-related symptom burden and work outcomes among cancer survivors who have completed primary antineoplastic treatment. To the best of our knowledge, this is the first systematic review that has included all cancer-related symptoms or complications that have been examined in the literature in association with work outcomes. In this review, body image issues and oral dysfunction were consistently demonstrated to be associated with lower employment rates, while fatigue and depression were linked to lower levels of work ability.

Among the studies included in this review, work status (employment, RTW, early retirement, and work disability) was most commonly studied. However, it should be noted that work status alone only captures extreme consequences where there is a complete loss of employment. Cancer survivors who remain employed can experience inferior outcomes at work, manifesting as decreased productivity, reduced working hours, and absenteeism [5]. This was evident from our review where cognitive impairment, fatigue, and depression were more consistently shown to be associated with reduced work ability or productivity than to lower employment rates. It is also important to emphasize that measures of unpaid work, such as household chores, caregiving, childcare, or volunteering, were not investigated in any of the studies reviewed. These outcomes hold significant value to patients and are an important component of productivity valuation [60]. Therefore, future studies should consider a wider range of work outcomes beyond changes in work status to comprehensively describe poor work outcomes that result from cancer-related complications. Focusing on work status alone may risk neglecting cancer survivors who are employed but face difficulties at work or reduced productivity beyond the scope of paid work.

Our review also showed that symptom burden was overwhelmingly measured using self-reported outcomes. Although patient-reported instruments are often considered to be at high risk of measurement bias, these tools have been shown to capture symptom burden among cancer survivors more accurately than clinician-reported outcomes [61]. Patient-reported outcomes are also more relevant than performance outcomes as they are closely related to how cancer-related complications affect daily activities from the perspective of the patients themselves. Nevertheless, it is important to select PROs that have been validated in the population of interest. In a number of the studies reviewed, single items or subscales from health-related quality-of-life questionnaires were used to measure symptom burden. This approach should be avoided unless validation of these components has previously been carried out. Furthermore, as symptom burden can be measured in terms of multiple dimensions, such as severity or frequency, further investigation should be carried out to ascertain if the relationship between symptom burden and work outcomes changes depending on how the former is quantified. PROs that have established clinically important thresholds should also be considered to enable comparison between studies and pooling of data for quantitative synthesis.

Contrary to expectations, significant relationships were not consistently demonstrated in most of the studies evaluating cancer-related complications that have been widely reported to persist among cancer survivors beyond the treatment phase, such as cognitive impairment, fatigue, pain, anxiety, and depression. Only approximately one-third of studies evaluating work status and fatigue or depression showed significant associations while a small proportion of studies assessing anxiety symptoms and cognitive impairment reported significant findings. This could potentially be due to the influence of psychosocial and interpersonal factors, such as family support and professional relationships at the workplace, on how cancer-related symptoms impact work outcomes. Although these factors have been postulated to affect work outcomes, they are generally difficult to quantify and control for in studies, leading to wide variability in the effect measures estimated and the lack of statistically significant findings. An alternative explanation was the use of vote counting in this review, where the proportions of studies reporting significant findings for each symptom were compared to identify symptoms consistently associated with poor work outcomes. Ideally, estimates from studies should be pooled in meta-analyses for the purpose of quantitative synthesis [62]. However, this approach was not feasible as almost half of the studies reviewed did not report all effect measures or measures of dispersion, especially if symptoms were not significantly associated with work outcomes or were excluded during model building. A pooled estimate from the reported effect measures would thus be biased away from the null. While vote counting was the best possible approach to identify trends in this review, the strength of the relationship between symptom burden and work outcomes was most likely underestimated [62]. Non-significant findings in individual studies could be due to the lack of statistical power rather than the absence of a true effect, particularly as the number of eligible studies increased [62]. This was supported by an observation in our review where studies that reported significant associations between pain and employment/RTW all consisted of samples of more than 1000 participants. To allow quantitative synthesis of findings and robust conclusions to be drawn from pooled data, future studies should report all results, including those from univariate analysis and model building exercises.

There is a continual need for more research to demonstrate how specific cancer-related symptoms influence work and employment among cancer survivors. Most of the associations between specific symptoms and work outcomes were only evaluated in a relatively small number of studies, with 47 of 68 of the unique associations identified evaluated in less than 5 studies. Associations that were found to be consistently significant in the majority of studies were also investigated in less than 10 studies each. The majority of the studies included were also cross-sectional in nature. Due to the lack of longitudinal observations, it is not possible to rule out reverse causality, where the symptom burden observed could have resulted from inferior work outcomes. This is plausible for a number of psychological symptoms such as depression and anxiety, suggesting the need for more prospective cohort studies examining these symptoms. Furthermore, most of the eligible studies were conducted in high-income countries predominantly in Europe or the USA, where labor market conditions, work demands, and cultural values may not be representative of all settings. More work should be carried out to validate if findings from existing studies are generalizable to lower-income or non-Western countries. Exploring these research gaps will allow a more comprehensive picture of how cancer-related symptom burden influences work outcomes among cancer survivors.

A potential limitation of this review was the lack of restrictions on the cancer diagnoses of participants recruited in the studies, which can increase the heterogeneity of the pooled findings. The incidence of certain symptoms, or the association between symptoms and work outcomes, may also be unique to specific patient populations. For example, oral dysfunction was mainly investigated and found to be significantly associated with poor work outcomes among survivors of head and neck cancer. The inclusion of all cancer diagnoses, however, ensured that the findings of this review would be beneficial to the wider survivor population and can assist the development of rehabilitative services that cater to all survivors regardless of cancer diagnoses. Cancer-related complications that are demonstrated to have a consistent and strongly negative impact on work outcomes, even if unique to a particular group of cancer survivors, are still important to address in work rehabilitation programs.

As survivorship care improved over the last two decades, interventions have been established for various cancer-related complications that plague cancer survivors, ranging from pharmacotherapeutic options such as non-opioid and opioid analgesics for pain [63] to non-pharmacological approaches, for example, cognitive training for cognitive impairment [64], speech rehabilitation for oral dysfunction [65], and cognitive behavioral therapy for depressive and anxiety symptoms [66]. Corroborating our observation that several different symptoms are linked to poor work outcomes, a review of current evidence suggested that only a multidisciplinary approach incorporating physical, psychoeducational, and medical interventions is effective in improving RTW among cancer survivors [9]. On a health-system level, findings from this review are therefore highly valuable to guide the selection of components to be prioritized when designing complex interventions for work rehabilitation, ensuring the efficient use of resources. This is particularly important in resource-constrained settings where the focus of oncology care is often almost entirely on antineoplastic treatment with limited capacity available for survivorship services [67]. From a clinical perspective, our findings also serve to increase the awareness of care providers to discuss any impact on employment or work when cancer survivors present with symptoms that are linked to poor work outcomes.

In conclusion, this systematic review has comprehensively investigated the extent to which different cancer-related symptoms significantly impact work outcomes of cancer survivors. While a range of complications, including body image issues, oral dysfunction, fatigue, and depression, were found to be linked to poor work outcomes among cancer survivors, our review has indicated that more studies with comprehensively reported findings are needed to draw robust conclusions. A wider range of work outcomes should also be considered, including absenteeism, presenteeism, and unpaid work while symptom burden should be measured with PRO tools that have been validated in the cancer population. The findings of this review can assist clinicians and policy makers in making informed decisions about the symptoms that adversely affect work outcomes and should be targeted in work rehabilitation programs.

## Supplementary Information

Below is the link to the electronic supplementary material.

Supplementary file1 (DOCX 1145 KB)

## Data Availability

The datasets generated and/or analyzed during the current study are available from the corresponding author on reasonable request.
