# Supplementary material for: Investigating how cancer-related symptoms influence work outcomes among cancer survivors: a systematic review
Source: J Cancer Surviv. 2021 Aug 23;16(5):1065–78. doi: 10.1007/s11764-021-01097-5 (PMC9489549; doi:10.1007/s11764-021-01097-5)
Supplement: Supplementary file 1 — Supplementary file1 (DOCX 1145 KB) [file 11764_2021_1097_MOESM1_ESM.docx]

| Supplementary Material 1: Search strategy | 2 |
| --- | --- |
| Supplementary Material 2A: Study design and setting of studies reviewed | 5 |
| Supplementary Material 2B: Population characteristics of studies reviewed | 8 |
| Supplementary Material 3: Assessment of risk of bias in the studies reviewed | 16 |
| Supplementary Material 4: Proportion of studies reviewed evaluating specific symptoms and work outcomes | 19 |
| Supplementary Material 5A: Summary of symptoms evaluated by studies reviewed |  |
| Localised, neurological symptoms | 20 |
| Gastrointestinal, psychological symptoms | 22 |
| Respiratory, fatigue/sleep-related and other symptoms | 24 |
| Supplementary Material 5B: Summary of work outcomes evaluated by studies reviewed | 27 |
| Supplementary Material 6: Summary of findings from studies reviewed |  |
| Localised symptoms | 30 |
| Neurological symptoms | 42 |
| Gastrointestinal symptoms | 60 |
| Psychological symptoms | 69 |
| Respiratory symptoms | 82 |
| Fatigue/sleep-related symptoms | 85 |
| Other symptoms: hot flashes, rash, sexual dysfunction, urinary problems | 98 |

**Supplementary Material 1: Search strategy**

First search: 1 Jan 1999 – 31 Dec 2019

Final search: 30 Oct 2020

**PubMed**

| Search | Query |
| --- | --- |
| #1 | “Cancer Survivor” |
| #2 | survivor*[tiab]AND (neoplasm*[tiab] OR cancer[tiab] OR oncology[tiab] OR carcin*[tiab]) |
| #3 | #1 OR #2 |
| #4 | “Absenteeism“ OR “Sick Leave“ OR “Return to Work“ OR “Work Performance“ OR “Unemployment“ OR “Retirement“ OR “Work Capacity Evaluation“ OR (“Efficiency“ AND (work*[tiab] OR job*[tiab])) OR Housekeeping OR Job Satisfaction OR absenteeism[tiab] OR work absence*[tiab] OR disability absence*[tiab] OR sickness absence*[tiab] OR sick day*[tiab] OR illness day*[tiab] OR work day loss*[tiab] OR work time loss*[tiab] OR medical leave*[tiab] OR sick leave*[tiab] OR sickness leave*[tiab] OR disability leave*[tiab] OR presenteeism[tiab] OR sickness presence[tiab] OR return-to-work[tiab] OR back-to-work[tiab] OR re-employment[tiab] OR job re-entry[tiab] OR work productivit*[tiab] OR work function*[tiab] OR work participation[tiab] OR work performance*[tiab] OR employment status[tiab] OR work status[tiab] OR unemployment[tiab] OR unemployed[tiab] OR work abilit*[tiab] OR work ability[tiab] OR work disabilit*[tiab] OR work inabilit*[tiab] OR work capacit*[tiab] OR work incapacity[tiab] OR work capabilit*[tiab] OR work incapabilit*[tiab] OR work inhibition*[tiab] OR work limitation*[tiab] OR work function*[tiab] OR job function*[tiab] OR work participation[tiab] OR job performanc*[tiab] OR vocational performanc*[tiab] OR performance at work[tiab] OR work productivit*[tiab] OR work efficien*[tiab] OR job efficien*[tiab] OR work retention[tiab] OR work sustainability[tiab] OR retirement*[tiab] OR job satisfaction* [tiab] OR work satisfaction* [tiab] OR house work* [tiab] OR housework [tiab] OR unpaid work [tiab] OR unpaid labo?r [tiab] OR housekeep* [tiab] OR domestic labo?r [tiab] OR domestic work [tiab] |
| #5 | #3 AND #4 |

Publication date: AND (("1999/01/01"[Date - Publication] : "2020/10/30"[Date - Publication]))

**Embase**

| Search | Query |
| --- | --- |
| #1 | (‘cancer survivor’/exp OR ‘cancer survivor’ OR ‘cancer survival’/exp OR ‘cancer survival’) |
| #2 | (‘survivor*’ AND (neoplasm* OR cancer OR oncology OR carcin*)) |
| #3 | #1 OR #2 |
| #4 | (‘absenteeism’/exp OR ‘job performance’/exp OR presenteeism/exp OR (productivity/exp AND (work:ab,ti OR job:ab,ti)) OR ‘return to work’/exp OR ‘work capacity’/exp OR ‘medical leave’/exp OR ‘employment status’/exp OR unemployment/exp OR retirement/exp OR 'housekeeping'/exp OR ‘job satisfaction’/exp OR 'housekeep*':ab,ti OR ‘house work’:ab,ti OR ‘housework’:ab,ti OR ‘unpaid work’:ab,ti OR ‘unpaid labo?r’:ab,ti OR ‘domestic labo?r’:ab,ti OR ‘domestic work’:ab,ti OR ‘absenteeism’:ab,ti OR ‘work absence*’:ab,ti OR ‘disability absence*’:ab,ti OR ‘sickness absence*’:ab,ti OR ‘sick day*’:ab,ti OR ‘illness day*’:ab,ti OR ‘work day loss*’:ab,ti OR ‘work time loss*’:ab,ti OR ‘medical leave*’:ab,ti OR ‘sick leave*’:ab,ti OR ‘sickness leave*’:ab,ti OR ‘disability leave*’:ab,ti OR ‘presenteeism’:ab,ti OR ‘sickness presence’:ab,ti OR ‘return-to- work’:ab,ti OR ‘back- to-work’:ab,ti OR re-employment:ab,ti OR ‘job re-entry’:ab,ti OR ‘employment status’:ab,ti OR ‘work status’:ab,ti OR unemployment:ab,ti OR unemployed:ab,ti OR ‘work abilit*’:ab,ti OR workabilit*:ab,ti OR ‘work disabilit*’:ab,ti OR ‘work inabilit*’:ab,ti OR ‘work capacit*’:ab,ti OR ‘work incapacit*’:ab,ti OR ‘work capabilit*’:ab,ti OR ‘work incapabilit*’:ab,ti OR ‘work inhibition*’:ab,ti OR ‘work limitation*’:ab,ti OR ‘work function*’:ab,ti OR ‘job function*’:ab,ti OR ‘work participation’:ab,ti OR ‘job performanc*’:ab,ti OR ‘vocational performance’:ab,ti OR ‘performance at work’:ab,ti OR ‘work productivit*’:ab,ti OR ‘work efficien*’:ab,ti OR ‘job efficien*’:ab,ti OR ‘work retention’:ab,ti OR ‘work sustainability’:ab,ti OR retirement:ab,ti OR ‘job satisfaction*’:ab,ti OR ‘work satisfaction*’:ab,ti) |
| #4 | #3 AND #4 |
| #5 | #5 AND ('human'/de) AND ('article'/it OR 'review'/it) |

Publication date: “AND [1999-2020]/py”

**PsycInfo**

| Search | Query |
| --- | --- |
| #1 | (Exp neoplasms/ AND exp survivors/) |
| #2 | ((cancer.mp. OR neoplasm*.mp. OR oncology.mp. OR carcin*.mp.) AND (survivor*.mp. OR survival.mp.)) |
| #3 | #1 OR #2 |
| #4 | Exp employee absenteeism/ OR exp employee productivity/ OR exp reemployment/ OR exp employee leave benefits/ OR exp unemployment/ OR exp job performance/ OR exp employment status/ OR exp disability evaluation/ OR exp employee efficiency/ OR exp retirement/ OR exp household management/ OR exp job satisfaction/ OR Absenteeism.mp. OR presenteeism.mp. OR (sick leave).mp. OR (job re-entry).mp. OR (job performance*).mp. OR retirement.mp. OR productivity.mp. OR employment.mp. OR (employment status).mp. OR unemployment.mp. OR (work absence*).mp. OR (disability absence*).mp. OR (sickness absence*).mp. OR (sick day*).mp. OR (illness day*).mp. OR (work day loss*).mp. OR (work time loss*).mp. OR (medical leave*).mp. OR (sick leave*).mp. OR (sickness leave*).mp. OR (disability leave*).mp. OR (return to work).mp. OR (back to work).mp. OR (re-employment).mp. OR (job re-entry).mp. OR (work productiv*).mp. OR (work status).mp. OR unemployed.mp. OR (work ability*).mp. OR workability*.mp. OR (work disabilit*).mp. OR (work inability*).mp. OR (work capacit*).mp. OR (work incapacit*).mp. OR (work capabilit*).mp. OR (work incapabilit*).mp. OR (work inhibition*).mp. OR (work limitation*).mp. OR (work function*).mp. OR (job function*).mp. OR (work participation).mp. OR (job performanc*).mp. OR (vocational performance).mp. OR (performance at work).mp. OR (work productivit*).mp. OR (work efficien*).mp. OR (job efficien*).mp. OR (work retention).mp. OR (work sustainability).mp. OR (housework).mp. OR (house work).mp. OR (housekeep*).mp. OR (unpaid work).mp. OR (unpaid labo?r).mp. OR (domestic labo?r).mp. OR (domestic work).mp. OR (job satisfaction*).mp. OR (work satisfaction*).mp. |
| #5 | #3 AND #4 |

Publication date: yr="1999 - 2020"

**CINAHL**

| Search | Query |
| --- | --- |
| #1 | (MM "Cancer Survivors") OR (MM "Cancer Patients") OR ((TI survivor OR AB survivor) AND (TI cancer OR AB cancer OR TI neoplasm* OR AB neoplasm* OR TI oncology OR AB oncology OR TI carcin* OR AB carcin*)) |
| #2 | (MM “Absenteeism”) OR (MM “Presenteeism”) OR (MM “Sick Leave”) OR (MM “Job Re-Entry”) OR (MM “Job Performance”) OR (MM “Retirement”) OR (MM “Productivity”) OR (MM “Work Capacity Evaluation”) OR (MM “Employment”) OR (MM “Employment Status”) OR (MM “Unemployment”) OR (MM “Quality of Working Life”) OR (MM “Home Maintenance”) OR (MM “Job Satisfaction”) OR TI absenteeism* OR AB absenteeism* OR TI “work absence*” OR AB “work absence*” OR TI “disability absence*” OR AB “disability absence*” OR TI “sickness absence*” OR AB “sickness absence*” OR TI “sick day*” OR AB “sick day*” OR TI “illness day*” OR AB “illness day*” OR TI “work day loss*” OR AB “work day loss* OR TI “work time loss*” OR AB “work time loss*” OR TI “medical leave*” OR AB “medical leave*” OR TI “sick leave*” OR AB “sick leave*” OR TI “sickness leave*” OR AB “sickness leave*” OR TI “disability leave*” OR AB “disability leave*” OR TI presenteeism OR AB presenteeism OR TI “sickness presence” OR AB “sickness presence” OR TI “return to work” OR AB “return to work” OR TI “back to work” OR AB “back to work” OR TI “Re-employment” OR AB “Re-employment” TI “job re-entry” OR AB “job re-entry” OR TI “work productivit*” OR AB “work productivit*” OR ((TI work OR AB work OR TI job OR AB job OR TI vocational OR AB vocational) AND (TI “Performanc*” OR TI “Efficien*” OR TI “Productiv*” OR “capacity* OR TI disabilit* OR AB disabilit*)) OR TI “employment status” OR AB “employment status” OR TI “work status” OR AB “work status” OR TI “unemployment” OR AB “unemployment” OR TI “unemployed” OR AB “unemployed” OR TI “work abilit*” OR AB “work abilit*” OR TI “workabilit*” OR AB workabilit*” OR TI “work disabilit*” OR AB work disabilit*” OR TI “work inabilit*” OR AB work inabilit*” OR OR TI “work retention” OR AB “work retention” OR TI “work sustainability” OR AB”work sustainability” OR TI “retirement” OR AB “retirement” OR AB “job satisfaction*” OR AB “job satisfaction*” OR TI “work satisfaction*” OR AB “job satisfaction*” OR TI “housekeep*” OR AB “housekeep*” OR TI “housework” OR AB “housework” OR TI “house work” OR AB “house work” OR TI “unpaid work” OR AB “unpaid work” OR TI “unpaid labo?r” OR AB “unpaid labo?r” OR TI “domestic labo?r” OR AB “domestic labo?r” OR TI “domestic work” OR AB “domestic work” |
| #3 | S1 AND S2 |

Publication date: Limiters - Published Date: 19990101-20201030)

**Scopus**

| Search | Query |
| --- | --- |
| #1 | TITLE-ABS-KEY (cancer OR neoplasm OR oncology OR carcin*) AND TITLE-ABS-KEY (survivor* OR survival) |
| #2 | TITLE-ABS-KEY (absenteeism OR presenteeism OR “sick leave” OR “job re-entry” OR “job performance*” OR retirement OR productivity OR “Work Capacity Evaluation” OR employment OR “employment status” OR unemployment OR “Quality of Working Life” OR “work absence*” OR “disability absence*” OR “sickness absence*” OR “sick day*” OR “illness day*” OR “work day loss*” OR “work time loss*” OR “medical leave*” OR “sick leave*” OR “sickness leave*” OR “disability leave*” OR “return to work” OR “back to work” OR “re-employment” OR “job re-entry” OR “work productiv*” OR “work status” OR unemployed OR “work ability*” OR workability* OR “work disabilit*” OR “work inability*” OR “work capacit*” OR “work incapacit*” OR “work capabilit*” OR “work incapabilit*” OR “work inhibition*” OR “work limitation*” OR “work function*” OR “job function*” OR “work participation” OR “job performanc*” OR “vocational performance” OR “performance at work” OR “work productivit*” OR “work efficien*” OR “job efficien*” OR “work retention” OR “work sustainability” OR “housekeep*” OR “house work” OR “housework” OR “unpaid work” OR “unpaid labo?r” OR “domestic labo?r” OR “domestic work” OR “job satisfaction*” OR “work satisfaction*”) |
| #3 | #1 AND #2 |

Publication date:

AND ( LIMIT-TO ( PUBYEAR , 2020 ) OR LIMIT-TO ( PUBYEAR , 2019 ) OR LIMIT-TO ( PUBYEAR , 2018 ) OR LIMIT-TO ( PUBYEAR , 2017 ) OR LIMIT-TO ( PUBYEAR , 2016 ) OR LIMIT-TO ( PUBYEAR , 2015 ) OR LIMIT-TO ( PUBYEAR , 2014 ) OR LIMIT-TO ( PUBYEAR , 2013 ) OR LIMIT-TO ( PUBYEAR , 2012 ) OR LIMIT-TO ( PUBYEAR , 2011 ) OR LIMIT-TO ( PUBYEAR , 2010 ) OR LIMIT-TO ( PUBYEAR , 2009 ) OR LIMIT-TO ( PUBYEAR , 2008 ) OR LIMIT-TO ( PUBYEAR , 2007 ) OR LIMIT-TO ( PUBYEAR , 2006 ) OR LIMIT-TO ( PUBYEAR , 2005 ) OR LIMIT-TO ( PUBYEAR , 2004 ) OR LIMIT-TO ( PUBYEAR , 2003 ) OR LIMIT-TO ( PUBYEAR , 2002 ) OR LIMIT-TO ( PUBYEAR , 2001 ) OR LIMIT-TO ( PUBYEAR , 2000 ) OR LIMIT-TO ( PUBYEAR , 1999 ))

**Supplementary Material 2A: Study design and setting of studies reviewed**

| Study | N | Country | Study design | Age restriction |
| --- | --- | --- | --- | --- |
| Agarwal 2017 | 250 | India | Cross-sectional | <60 |
| Alleaume 2018 | 969 | France | Cross-sectional | 18 to 54 at time of diagnosis |
| Azarkish 2015 | 175 | Iran | Cross-sectional | 20 to 60 |
| Back 2019 | 146 | Australia | Prospective cohort | None |
| Balak 2008 | 72 | The Netherlands | Retrospective cohort | 18 to 65 |
| Behringer 2016 | 5306 | Germany | Prospective cohort | 18 to 60 |
| Bennett 2018 | 3913 | United Kingdom | Cross-sectional | <60 at time of diagnosis |
| Boscher 2020 | 1393 | France | Cross-sectional | >=18 |
| Boyages 2016 | 269 | Australia | Cross-sectional | None |
| Brick 2019 | 24 | USA | Prospective cohort | <75 |
| Calvio 2010 | 122 | USA | Cross-sectional | 18 to 65 |
| Check 2019 | 675 | USA | Cross-sectional | None |
| Chen 2012 | 511 | USA | Cross-sectional | None |
| Chen 2019 | 174 | Taiwan | Cross-sectional | 20 to 64.5 |
| Cheng 2016 | 30 | Hong Kong | Cross-sectional | 18 to 65 |
| Cheng 2018 | 267 | China | Cross-sectional | 20 to 60 |
| Cheville 2011 | 2405 | USA | Prospective cohort | <65 |
| Cooper 2013 | 332 | United Kingdom | Prospective cohort | >18 |
| Cox-Martin 2020 | 1702 | USA | Cross-sectional | 25 to 64 |
| Dahl 2015, 2016 | 563 | Norway | Prospective cohort | None |
| Dahl 2019 | 1198 | Norway | Cross-sectional | 19 to 39 at time of diagnosis |
| Dahl 2020 | 354 | Norway | Cross-sectional | <=75 |
| Dieluweit 2011 | 820 | Germany | Cross-sectional | 20 to 46 |
| Dorland 2018 | 384 | The Netherlands | Prospective cohort | 18 to 65 |
| Duijts 2017 | 252 | The Netherlands | Prospective cohort | 18 to 64 |
| Dumas 2020 | 1874 | France | Prospective cohort | 18 to 57 |
| Dunberger 2013 | 606 | Sweden | Cross-sectional | <80 |
| Ekenga 2019 | 347 | USA | Prospective cohort | >40 |
| Fantoni 2010 | 379 | France | Retrospective cohort | 18 to 60 |
| Gudbergsson 2008 | 431 | Norway | Cross-sectional | 25 to 57 at time of diagnosis |
| Hansen 2008 | 100 | USA | Cross-sectional | 20 to 70 |
| Ho 2018 | 168 | Singapore | Cross-sectional | 18 to 65 |
| Kenzik 2015 | 1007 | USA | Prospective cohort | None |
| Kerns 2020 | 1815 | USA | Cross-sectional | None |
| Kim 2014 | 906 | South Korea | Cross-sectional | >18 |
| Kiserud 2015 | 233 | Norway | Cross-sectional | <=50 at time of diagnosis |
| Kiserud 2016 | 312 | Norway | Cross-sectional | >=18 at time of treatment |
| Koch 2015 | 55 | Germany | Cross-sectional | <=60 |
| Landeiro 2018 | 121 | Brazil | Prospective cohort | 18 to 57 |
| Lavigne 2008 | 83 | USA | Cross-sectional | None |
| Lee 2017 | 299 | South Korea | Prospective cohort | 20 to 65 |
| Lindbohm 2014 | 1111 | Finland, Norway, Denmark, Iceland | Cross-sectional | 25 to 57 at time of diagnosis |
| Magyari 2017 | 140 | Hungary | Cross-sectional | <65 |
| Mehnert 2017 | 1148 | Germany | Prospective cohort | 18 to 60 |
| Murray 2019 | 205 | United Kingdom | Retrospective cohort | 18 to 58 |
| Musti 2018 | 503 | Italy | Cross-sectional | 18 to 65 |
| Nakamura 2017 | 82 | Japan | Cross-sectional | <65 |
| Ng 2020 | 74 | France | Prospective cohort | None |
| Nieuwenhuijsen 2009 | 45 | The Netherlands | Prospective cohort | 18 to 55 |
| Pryce 2007 | 328 | United Kingdom | Cross-sectional | None |
| Quinlan 2009, 2011 | 372 | Canada | Prospective cohort | >18 |
| Schmidt 2019 | 137 | Germany | Prospective cohort | <=65 |
| Smoot 2010 | 144 | USA | Cross-sectional | >18 |
| So 2020 | 73 | Canada | Cross-sectional | 18 to 65 |
| Spelten 2003 | 235 | The Netherlands | Prospective cohort | 18 to 60 |
| Steiner 2008 | 100 | USA | Cross-sectional | 21 to 64 at time of diagnosis |
| Syrjala 2004 | 281 | USA | Prospective cohort | >=18 |
| Tamminga 2016 | 223 | The Netherlands | Cross-sectional | 18 to 65 |
| Tevaarwerk 2013 | 356 | USA, Peru | Cross-sectional | <65 |
| Van Muijen 2014, 2017, 2019 | 392 | The Netherlands | Prospective cohort | 18 to 64 |
| Vartanian 2006 | 301 | Brazil | Cross-sectional | None |
| Verdonck-de Leeuw 2010 | 53 | The Netherlands | Cross-sectional | <64 at time of diagnosis |
| Von Ah 2017, 2018 | 68 | USA | Cross-sectional | 18 to 70 |
| Wolvers 2019 | 89 | The Netherlands | Prospective cohort | 18 to 60 |
| Yoo 2013 | 858 | South Korea | Cross-sectional | None |
| Zanville 2016 | 22 | USA | Prospective cohort | <=69 |
| Zeng 2017 | 159 | China | Cross-sectional | >=18 |
| Zomkowski 2020 | 62 | Brazil | Cross-sectional | None |

**Supplementary Material 2B: Population characteristics of studies reviewed**

| Study | Age | Dx | Stage | Act dis | Time since dx | Time since tx | S | C | R | Work sector |
| --- | --- | --- | --- | --- | --- | --- | --- | --- | --- | --- |
| Agarwal 2017 | Median = 48 years | H&N | All | No | NR | >6 months  (Mean = 19 months) | 75% | 57% | 100% | White collar: 57.6%; manual work: 42.4% |
| Alleaume 2018 | [Age groups at time of diagnosis =  18-39: 20.3%;  40-49: 55.4%;  50-54: 24.3%] | Various | NR | NR | 5 years | NR | NR | 55% | 66% | Execution: 56%; supervisors: 44% |
| Azarkish 2015 | Mean = 44 years | Breast | NR | NR | NR | 1 year | NR | NR | NR | NR |
| Back 2019 | [Age groups at time of diagnosis =  <40: 40%;  >40: 60%] | Brain | NR | No | NR | [3 years after RT] | 100% | 88% | 100% | NR |
| Balak 2008 | [Mean age at time of diagnosis =  49 years] | Breast | 0-II | No | [2 years since sick leave] | NR | 100% | 57% | 46% | Healthcare: 33%; postal services: 18% |
| Behringer 2016 | Median = 34 years | Hemato | NR | NR | NR | 5 years | NR | 100% | 100% | NR |
| Bennett 2018 | Mean = 56 years | Prostate | All | Yes | 18 to 42 months | NR | 49% | 3% | 30% | NR |
| Boscher 2020 | Median = 52 years | Breast | NR | Yes | NR | <=1 year: 26.8%; 1-3 years: 24.1%;  >=3 years: 49.1% | NR | 77% | NR | NR |
| Boyages 2016 | <55 years: 40%;  >=55 years: 60% | Breast | I-III | No | NR | >1 year  (<5 years: 67%;  >=5 years: 33%) | NR | NR | NR | Education: 25%; health service: 18% |
| Brick 2019 | Mean = 61 years | Breast | I-IIIa | No | NR | [6 months after surgery and start of chemotherapy or endocrine therapy] | 100% | 21% | NR | Adm personnel: 46%; clerical/sales: 33% |
| Calvio 2010 | Mean = 45 years | Breast | I-III | No | NR | 1 to 10 years (Mean = 3 years) | 97% | 87% | 73% | Prof/tech/sc: 46%; mgmt/adm: 31% |
| Check 2019 | Mean = 62 years | H&N | All | No | 1 to 22 years (Median = 6 years) | >1 year | 3% | 68% | 98% | NR |
| Chen 2012 | Median = 44 years | Hemato | NR | NR | >5 years | NR | NR | 64% | 93% | NR |
| Chen 2019 | Mean = 53 years | H&N | All | NR | NR | >6 months  (Mean = 33 months) | 100% | 36% | 39% | Unskilled: 70%; skilled: 26% |
| Cheng 2016 | <40 years: 3.3%;  40-49 years: 40.0%;  >=50 years: 56.7% | Breast | I-III | No | NR | >6 months  (Mean = 2.9 years) | 87% | 67% | 90% | Clerical/sales/service/nontechnical worker: 90% |
| Cheng 2018 | 20-39: 44%;  40-49: 52%;  >=50: 4% | Breast | I-III | No | NR | >2 years  (2-3 years: 45%;  >=3: 55%) | 89% | 45% | 79% | NR |
| Cheville 2011 | NR | Lung | All | Yes | 1 to 5 years | NR | NR | NR | NR | NR |
| Cooper 2013 | Mean = 55 years | Various | NR | No | [Median time since start of sick leave = 4.7 months] | [Median time since start of tx = 3.9 months] | 86% | 38% | 50% | White collar: 58%; blue collar: 24% |
| Cox-Martin 2020 | 25-34 years: 3.3%; 35-44 years: 7.9%; 45-54 years: 24%; 55-64 years: 64.8% | Various | NR | NR | NR | NR | NR | NR | NR | NR |
| Dahl 2015, 2016 | Mean = 61 years | Prostate | NR | NR | NR | Mean = 3 years | 100% | NR | 13% | NR |
| Dahl 2019 | Mean = 50 years | Various | NR | No | >6 years  (Mean = 17 years) | NR | 84% | 66% | 67% | NR |
| Dahl 2020 | Median = 50 years | Gynae | NR | Yes | Mean = 11 years | NR | 83% | 17% | 17% | NR |
| Dieluweit 2011 | Mean = 30 years | Various | NR | NR | >5 years  (Mean = 13.7 years) | NR | 72% | 91% | 58% | NR |
| Dorland 2018 | Mean = 51 years | Various | NR | Yes | 6, 12 and 18 months | NR | 15% | 71% | 13% | NR |
| Duijts 2017 | Mean = 51 years | Various | NR | Yes | 2 to 4 years | NR | NR | 73% | NR | NR |
| Dumas 2020 | Mean = 47 years | Breast | I-III | No | 2 years  (Median = 23 months) | NR | 100% | 66% | NR | Prof/mngrs: 24.6%; clerks: 39.4% |
| Dunberger 2013 | Mean = 64 years | Gynae | NR | No | NR | Mean = 83 months | 90% | 33% | 100% | NR |
| Ekenga 2019 | Median = 42 years | Breast | 0-IIA | No | NR | [>2 years after surgery] | 100% | 33% | 63% | NR |
| Fantoni 2010 | Mean = 48 years | Breast | NR | No | >2 years | NR | NR | 36% | 78% | Off workers: 39.7%;  Intermediate professions: 23.9% |
| Gudbergsson 2008 | Mean = 50 years | Various | NR | No | NR | 2 to 6 years | NR | NR | NR | NR |
| Hansen 2008 | Mean = 50 years | Breast | I-III | No | Mean = 3.8 years | NR | 93% | 79% | 67% | Mngr: 25% |
| Ho 2018 | Median = 53 years | Breast | All | Yes | >1 year  (Median = 4 years) | NR | 99% | 65% | 63% | NR |
| Kenzik 2015 | <=54: 26%;  55-64: 25%;  65-74: 27%;  >=75: 22% | C/rectal | All | NR | >1 year | NR | 92% | 52% | 14% | NR |
| Kerns 2020 | Median = 37 years | Testic | NR | No | NR | >1 year  (Median = 3.8 years) | NR | 100% | 0% | NR |
| Kim 2014 | <50 years: 7%;  >=50 years: 93% | Lung | 0-III | No | Median =  4.1 years | NR | 100% | 34% | 14% | NR |
| Kiserud 2015 | Median = 48 years | Hemato | NR | NR | >5 years | NR | NR | 86% | 78% | NR |
| Kiserud 2016 | Mean = 54 years | Hemato | NR | No | Mean =  12.4 years | Mean = 9.7 years | NR | 100% | NR | NR |
| Koch 2015 | Mean = 53 years | H&N | All | No | >2 years  (Mean = 5.6 years) | >2 years | 95% | 22% | 67% | Med phy work: 33%;  hard phy work: 20% |
| Landeiro 2018 | Mean = 45 years | Breast | I-III | No | 24 months | NR | 99% | 93% | 84% | Domestic serv: 20.7%;  business serv: 11.6% |
| Lavigne 2008 | Mean = 53 years | Breast | I-III | No | NR | >12 months (Mean = 36 months) | 100% | NR | 100% | NR |
| Lee 2017 | 20-44: 55%;  45-54: 43%;  55-65: 9% | Breast | I-III | No | Up to 36 months | NR | 100% | 78% | 87% | NR |
| Lindbohm 2014 | <55 years: 48%;  55-59 years: 35%;  60-64 years: 17% | Breast | NR | No | 1 to 8 years | NR | NR | 41% | NR | White collar: 74%; blue collar: 26% |
| Magyari 2017 | Mean = 45 years | Hemato | All | No | NR | NR | NR | 100% | 74% | NR |
| Mehnert 2017 | Mean = 49 years | Various | All | Yes | NR | [1 year from rehab] | NR | NR | NR | Off workers: 75.1%;  manual work: 18.4% |
| Murray 2019 | Mean = 38 years | Various | NR | Yes | 18 months | NR | 44% | 42% | 25% | Engineering: 30%; aircrew: 21% |
| Musti 2018 | Mean = 52 years | Breast | NR | NR | >2 years | NR | 100% | 50% | 73% | Off staff/sales asst: 62.2%;  labourers: 21.5% |
| Nakamura 2017 | [Mean age at time of diagnosis =  43 years] | Gynae | I-II | No | NR | >1 year  (Mean = 4.5 years) | 82% | 67% | 67% | NR |
| Ng 2020 | Mean = 36 years | Brain | Grade II | No | [Mean time from diagnosis to treatment = 34.4 months] | Up to 12 months | 100% | 32% | 10% | NR |
| Nieuwenhuijsen 2009 | Mean = 44 years | Various | NR | No | [12 months after first day of sick leave] | NR | 66% | 33% | 11% | NR |
| Pryce 2007 | Mean = 50 years | Various | NR | NR | NR | NR | 65% | 59% | 52% | Skilled/operational - non-manual: 54%; mngr/technical: 19% |
| Quinlan 2009, 2011 | NR | Breast | I-III | NR | NR | [6 to 12 months from surgery] | 100% | NR | NR | Business, finance, and administrative: 26%; sales and service: 19% |
| Schmidt 2019 | [Mean age at time of surgery = 51 years] | Breast | 0-IIIA | No | NR | [>1 years from surgery]  Median = 247 days | 100% | 60% | 93% | NR |
| Smoot 2010 | Mean = 56 years | Breast | NR | No | NR | >6 months | 100% | 70% | 74% | NR |
| So 2020 | Median = 53 years | H&N | All | NR | NR | [>=4 years disease free] Median = 7.3 years | NR | 93% | 100% | Service: 25%; manufacturing: 15% |
| Spelten 2003 | Mean = 42 years | Various | NR | No | [18 months after sick leave] | NR | >70% | >15% | >35% | NR |
| Steiner 2008 | NR | Various | NR | NR | Mean = 23 months | NR | 88% | 51% | 53% | NR |
| Syrjala 2004 | Mean = 36 years | Hemato | NR | NR | NR | Up to 5 years from transplant | NR | 80% | 17% | NR |
| Tamminga 2016 | Mean = 50 years | Thyroid | All | No | Mean = 9.9 years | NR | 99% | NR | NR | NR |
| Tevaarwerk 2013 | Median = 56 years | Various | NR | No | >6 months  (6 to 12 months: 13%;  12 to 24 months: 24%;  >24 months: 63%) | NR | NR | 84% | 62% | NR |
| Van Muijen 2014, 2017, 2019 | Mean = 51 years | Various | All | Yes | [36 months after sick leave] | NR | 72% | 73% | 60% | White collar: 29%; civil servant: 10%; blue collar: 37%; HCW: 24% |
| Vartanian 2006 | Median = 52 years | H&N | All | No | NR | [>2 years from disease remission] | 85% | 3% | 47% | NR |
| Verdonck-de Leeuw 2010 | Median = 59 years | H&N | All | No | NR | >2 years | 57% | 15% | 75% | NR |
| Von Ah 2017, 2018 | Mean = 52 years | Breast | 0-III | No | NR | >12 months (Mean = 59.5 months) | 99% | 62% | 65% | Prof: 43%; mgmt/adm: 16% |
| Wolvers 2019 | Mean = 48 years | Various | NR | NR | 12 months | NR | 89% | 89% | 64% | NR |
| Yoo 2013 | <50 years: 33%;  >=50 years: 67% | Gynae | I-IVA | No | NR | <5 years: 29%;  >=5 years: 71% | 85% | 29% | 27% | NR |
| Zanville 2016 | Mean = 50 years | Breast | 0-IIIC | No | NR | >1 year | 100% | 100% | 82% | Prof/tech: 50%; mngr/clerical/official: 45% |
| Zeng 2017 | <40 years: 15.2%; 40-49 years: 54.4%;  >=50 years: 30.4% | Breast | I-III | No | NR | >2 years  (Mean = 2.9 years) | 92% | 55% | 91% | White collar: 82%; blue collar: 11% |
| Zomkowski 2020 | Mean = 52 years | Breast | I-III | No | [Mean time from surgery = 48 months] | >1 month | 100% | 77% | 69% | NR |

Abbreviations – Dx: diagnosis; Act dis: active disease; tx: treatment; S: surgery; C: chemotherapy; R: radiotherapy; NR: not reported

**Supplementary Material 3 Assessment of risk of bias in the studies reviewed**

|  | Representativeness of exposed cohort | Selection of controls | Assessment of exposure | Outcome prior to study | Adjustment for prior work status | Adjustment for other factors | Assessment of outcome | Follow up period | Attrition rate |
| --- | --- | --- | --- | --- | --- | --- | --- | --- | --- |
| Agarwal 2017^51^ | 1 | NA | 4 | NA | 2 | 2 | 3 | NA | NA |
| Alleaume 2018^68^ | 1 | 1 | 2 | NA | 1 | 1 | 3 | NA | NA |
| Azarkish 2015^39^ | 3 | 1 | 4 | NA | 1 | 2 | 3 | NA | NA |
| Back 2019^69^ | 3 | 1 | 2 | 2 | 2 | 1 | 3 | 1 | 3 |
| Balak 2008^70^ | 3 | 1 | 3 | 1 | 1 | 1 | 2 | 1 | 1 |
| Behringer 2016^26^ | 2 | 1 | 4 | 2 | 1 | 1 | 3 | 1 | 3 |
| Bennett 2018^71^ | 2 | 1 | 4 | NA | 1 | 1 | 3 | NA | NA |
| Boscher 2020^54^ | 2 | 1 | 4 | NA | 2 | 2 | 3 | NA | NA |
| Boyages 2016^72^ | 2 | 1 | 4 | NA | 1 | 2 | 3 | NA | NA |
| Brick 2019^73^ | 3 | NA | 2 | 1 | 2 | 2 | 3 | 2 | 1 |
| Calvio 2010^45^ | 1 | NA | 4 | NA | 2 | 2 | 3 | NA | NA |
| Check 2019^31^ | 1 | NA | 4 | NA | 1 | 2 | 3 | NA | NA |
| Chen 2012^74^ | 2 | 1 | 4 | NA | 2 | 1 | 3 | NA | NA |
| Chen 2019^59^ | 1 | NA | 4 | NA | 1 | 2 | 3 | NA | NA |
| Cheng 2016^75^ | 2 | NA | 2 | NA | 2 | 1 | 3 | NA | NA |
| Cheng 2018^76^ | 2 | NA | 2 | NA | 2 | 2 | 3 | NA | NA |
| Cheville 2011^77^ | 1 | 1 | 4 | 2 | 2 | 2 | 3 | 1 | 3 |
| Cooper 2013^47^ | 1 | NA | 4 | 1 | 1 | 1 | 3 | 2 | 2 |
| Cox-Martin 2020^40^ | 2 | 1 | 4 | NA | 2 | 2 | 3 | NA | NA |
| Dahl 2015, 2016^78,79^ | 2 | 1 | 4 | 1 | 1 | 1 | 3 | 1 | 4 |
| Dahl 2019^18^ | 2 | NA | 2 | NA | 1 | 1 | 3 | NA | NA |
| Dahl 2020^36^ | 2 | NA | 4 | NA | 2 | 1 | 3 | NA | NA |
| Dieluweit 2011^55^ | 1 | 1 | 4 | NA | 2 | 1 | 3 | NA | NA |
| Dorland 2018^17^ | 2 | NA | 2 | 1 | 1 | 1 | 3 | 1 | 3 |
| Duijts 2017^20^ | 1 | NA | 4 | 1 | 1 | 1 | 3 | 1 | 2 |
| Dumas 2020^37^ | 1 | 1 | 2 | 1 | 1 | 1 | 3 | 1 | 1 |
| Dunberger 2013^80^ | 2 | 1 | 4 | NA | 2 | 2 | 3 | NA | NA |
| Ekenga 2019^22^ | 2 | 1 | 4 | 1 | 1 | 1 | 3 | 1 | 1 |
| Fantoni 2010^38^ | 1 | NA | 4 | 1 | 1 | 1 | 3 | 1 | 1 |
| Gudbergsson 2008^81^ | 1 | NA | 2 | NA | 1 | 2 | 3 | NA | NA |
| Hansen 2008^82^ | 3 | NA | 2 | NA | 2 | 1 | 3 | NA | NA |
| Ho 2018^16^ | 2 | NA | 2 | NA | 2 | 1 | 3 | NA | NA |
| Kenzik 2015^42^ | 1 | 1 | 4 | 1 | 1 | 1 | 3 | 2 | 3 |
| Kerns 2020^43^ | 2 | 1 | 4 | NA | 2 | 1 | 3 | NA | NA |
| Kim 2014^48^ | 2 | 1 | 4 | NA | 1 | 1 | 3 | NA | NA |
| Kiserud 2015^83^ | 3 | NA | 2 | NA | 2 | 1 | 3 | NA | NA |
| Kiserud 2016^27^ | 1 | NA | 4 | NA | 2 | 1 | 3 | NA | NA |
| Koch 2015^33^ | 3 | NA | 2 | NA | 2 | 2 | 3 | NA | NA |
| Landeiro 2018^32^ | 1 | 1 | 4 | 1 | 1 | 1 | 3 | 1 | 2 |
| Lavigne 2008^84^ | 1 | 1 | 2 | NA | 2 | 1 | 3 | NA | NA |
| Lee 2017^50^ | 3 | NA | 4 | 2 | 1 | 1 | 3 | 1 | 3 |
| Lindbohm 2014^29^ | 1 | 1 | 4 | NA | 1 | 1 | 3 | NA | NA |
| Magyari 2017^35^ | 1 | NA | 2 | NA | 2 | 2 | 3 | NA | NA |
| Mehnert 2017^41^ | 2 | NA | 2 | 1 | 1 | 1 | 3 | 1 | 3 |
| Murray 2019^25^ | 3 | 1 | 4 | 1 | 1 | 1 | 2 | 1 | 1 |
| Musti 2018^44^ | 1 | 1 | 4 | NA | 1 | 1 | 3 | NA | NA |
| Nakamura 2017^85^ | 2 | 1 | 4 | NA | 1 | 2 | 3 | NA | NA |
| Ng 2020^86^ | 3 | 1 | 4 | 1 | 1 | 2 | 3 | 2 | 1 |
| Nieuwenhuijsen 2009^53^ | 2 | 1 | 2 | 1 | 1 | 2 | 3 | 2 | 4 |
| Pryce 2007^23^ | 3 | 1 | 4 | NA | 2 | 2 | 3 | NA | NA |
| Quinlan 2009, 2011^87,88^ | 2 | 1 | 4 | 1 | 1 | 1 | 3 | 1 | 3 |
| Schmidt 2019^28^ | 3 | 1 | 2 | 1 | 1 | 1 | 3 | 1 | 2 |
| Smoot 2010^89^ | 2 | 1 | 4 | NA | 2 | 2 | 3 | NA | NA |
| So 2020^90^ | 2 | NA | 2 | NA | 1 | 2 | 3 | NA | NA |
| Spelten 2003^34^ | 1 | NA | 4 | 1 | 1 | 1 | 3 | 1 | 2 |
| Steiner 2008^46^ | 2 | 1 | 4 | NA | 1 | 2 | 3 | NA | NA |
| Syrjala 2004^91^ | 1 | 1 | 2 | 1 | 1 | 1 | 3 | 1 | 4 |
| Tamminga 2016^21^ | 2 | NA | 2 | NA | 2 | 1 | 3 | NA | NA |
| Tevaarwerk 2013^24^ | 4 | 1 | 4 | NA | 1 | 1 | 3 | NA | NA |
| Van Muijen 2014, 2017, 2019^92–94^ | 1 | 1 | 2 | 1 | 1 | 1 | 3 | 1 | 3 |
| Vartanian 2006^95^ | 2 | 1 | 4 | NA | 1 | 1 | 3 | NA | NA |
| Verdonck-de Leeuw 2010^49^ | 2 | NA | 4 | NA | 2 | 2 | 3 | NA | NA |
| Von Ah 2017, 2018^56,57^ | 2 | NA | 2 | NA | 2 | 1 | 3 | NA | NA |
| Wolvers 2019^19^ | 3 | NA | 2 | 1 | 1 | 1 | 3 | 1 | 3 |
| Yoo 2013^52^ | 2 | NA | 4 | NA | 1 | 1 | 3 | NA | NA |
| Zanville 2016^96^ | 3 | NA | 4 | 2 | 2 | 2 | 3 | 1 | 1 |
| Zeng 2017^58^ | 2 | NA | 2 | NA | 2 | 1 | 3 | NA | NA |
| Zomkowski 2020^30^ | 2 | NA | 4 | NA | 2 | 2 | 3 | NA | NA |

Note: Shaded cells indicate low risk of bias for Newcastle-Ottawa checklist item evaluated

**Supplementary Material 4: Proportion of studies reviewed evaluating (A), (B) specific symptoms and (C) work outcomes**


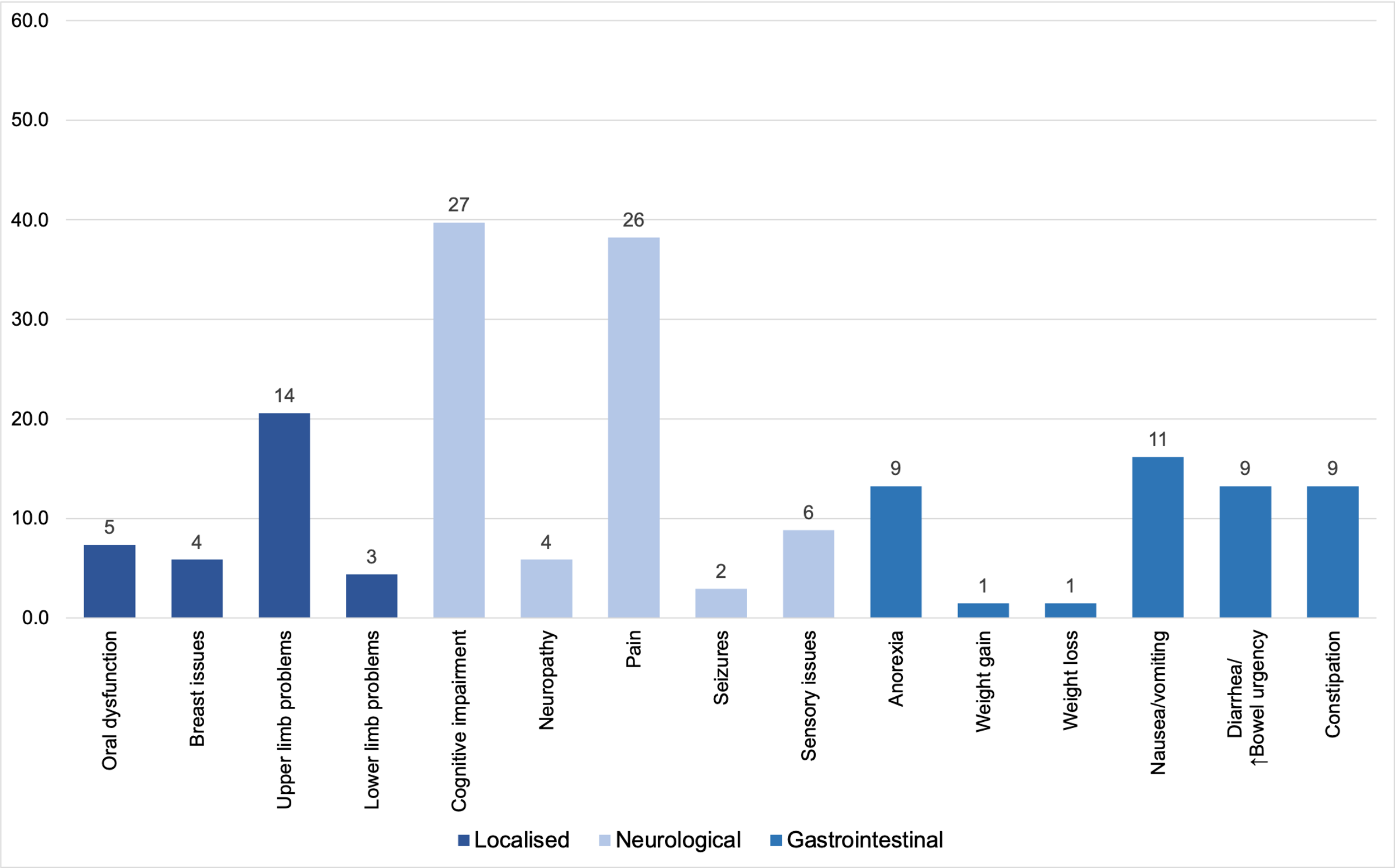

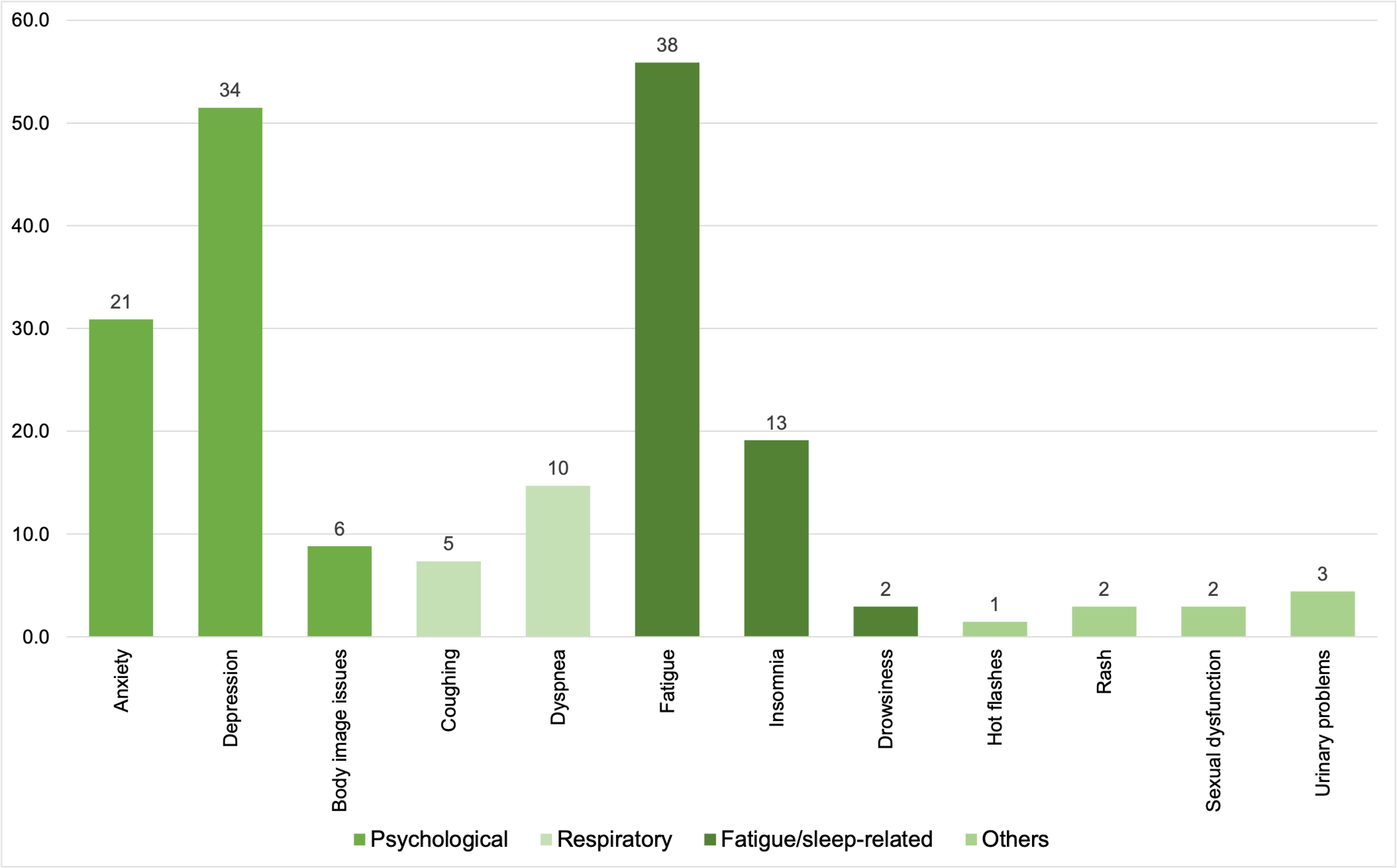


B

A


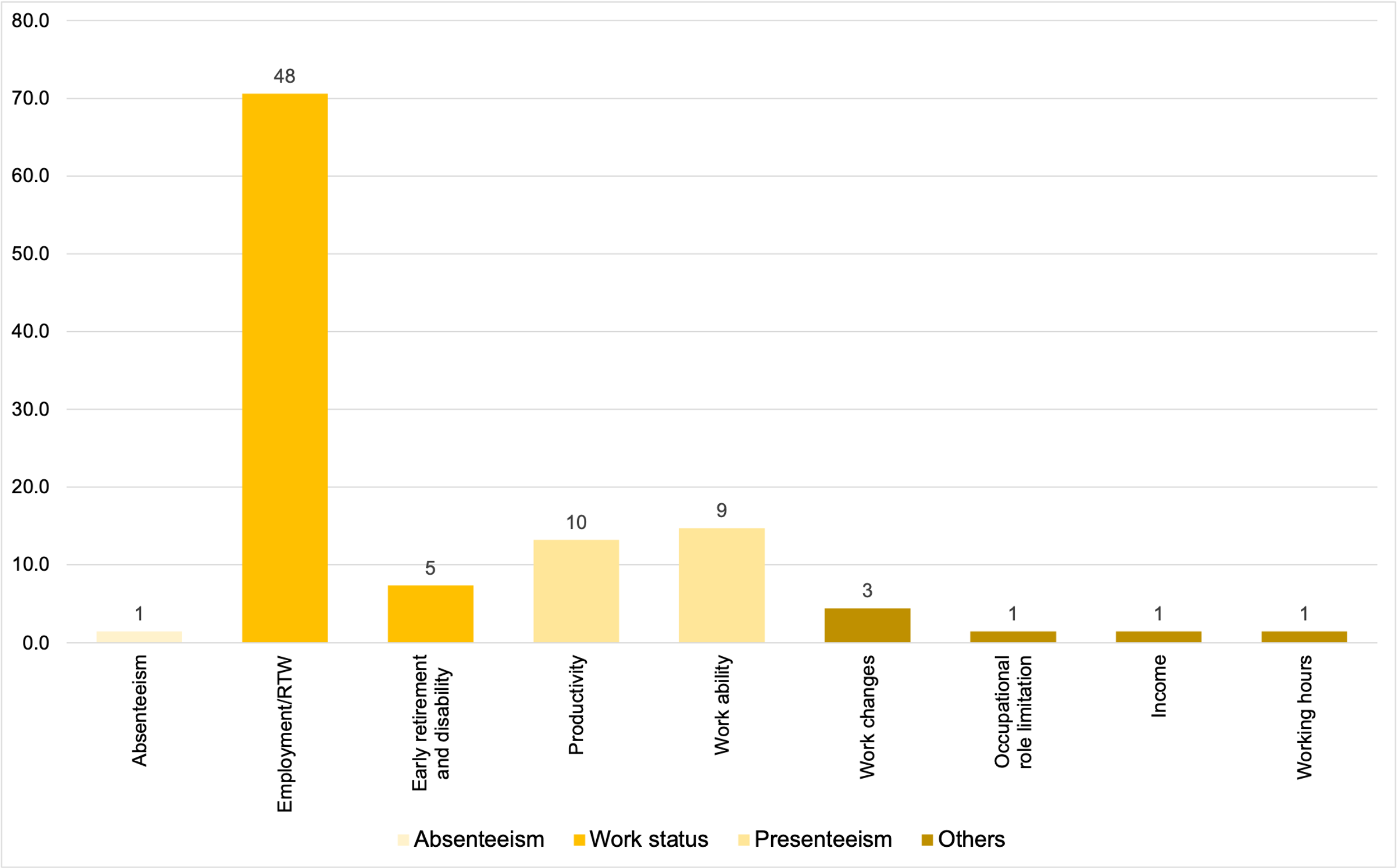


C

**Supplementary Material 5A: Summary of symptoms evaluated by studies reviewed**

| Study | Localised | | | | Neurological | | | | |
| --- | --- | --- | --- | --- | --- | --- | --- | --- | --- |
|  | Oral dysfunction | Breast issues | Upper limb problems | Lower limb problems | Cognitive impairment | Neuropathy | Pain | Seizures | Sensory issues |
| Agarwal 2017 | √ |  |  |  | √ |  | √ |  | √ |
| Alleaume 2018 |  |  |  |  |  |  | √ |  |  |
| Azarkish 2015 |  |  | √ |  |  |  | √ |  |  |
| Back 2019 |  |  |  |  | √ |  |  | √ |  |
| Balak 2008 |  |  | √ |  |  |  |  |  |  |
| Behringer 2016 |  |  |  |  |  |  |  |  |  |
| Bennett 2018 |  |  |  |  |  |  |  |  |  |
| Boscher 2020 |  |  |  |  | √ |  |  |  |  |
| Boyages 2016 |  |  | √ |  |  |  |  |  |  |
| Brick 2019 |  |  |  |  | √ |  |  |  |  |
| Calvio 2010 |  |  |  |  | √ |  | √ |  |  |
| Check 2019 | √ |  |  |  | √ | √ | √ |  | √ |
| Chen 2012 |  |  |  |  |  |  |  |  |  |
| Chen 2019 | √ |  | √ |  |  |  | √ |  | √ |
| Cheng 2016 |  |  |  |  | √ |  |  |  |  |
| Cheng 2018 |  |  |  |  | √ |  |  |  |  |
| Cheville 2011 |  |  |  |  |  |  |  |  |  |
| Cooper 2013 |  |  |  |  | √ |  | √ |  |  |
| Cox-Martin 2020 |  |  |  |  |  |  | √ |  |  |
| Dahl 2015, 2016 |  |  |  |  |  |  |  |  |  |
| Dahl 2019 |  |  |  |  |  |  |  |  |  |
| Dahl 2020 |  |  |  | √ |  |  | √ |  |  |
| Dieluweit 2011 |  |  |  |  | √ |  |  |  | √ |
| Dorland 2018 |  |  |  |  | √ |  |  |  |  |
| Duijts 2017 |  |  |  |  | √ |  | √ |  |  |
| Dumas 2020 |  | √ | √ |  | √ |  |  |  |  |
| Dunberger 2013 |  |  |  | √ |  |  |  |  |  |
| Ekenga 2019 |  |  |  |  |  |  |  |  |  |
| Fantoni 2010 |  |  | √ |  |  |  | √ |  |  |
| Gudbergsson 2008 |  |  |  |  |  |  |  |  |  |
| Hansen 2008 |  |  |  |  | √ |  |  |  |  |
| Ho 2018 |  | √ | √ |  | √ |  |  |  |  |
| Kenzik 2015 |  |  |  |  |  |  | √ |  |  |
| Kerns 2020 |  |  |  |  |  | √ | √ |  | √ |
| Kim 2014 |  |  |  |  | √ |  | √ |  |  |
| Kiserud 2015 |  |  |  |  |  |  |  |  |  |
| Kiserud 2016 |  |  |  |  | √ |  |  |  |  |
| Koch 2015 |  |  |  |  |  |  |  |  |  |
| Landeiro 2018 |  |  | √ |  |  |  | √ |  |  |
| Lavigne 2008 |  |  |  |  |  |  |  |  |  |
| Lee 2017 |  | √ | √ |  | √ |  | √ |  |  |
| Lindbohm 2014 |  |  |  |  |  |  | √ |  |  |
| Magyari 2017 |  |  |  |  |  |  |  |  |  |
| Mehnert 2017 |  |  |  |  |  |  | √ |  |  |
| Murray 2019 |  |  |  |  |  |  |  |  |  |
| Musti 2018 |  |  | √ |  |  |  | √ |  |  |
| Nakamura 2017 |  |  |  | √ |  |  |  |  |  |
| Ng 2020 |  |  |  |  |  |  |  | √ |  |
| Nieuwenhuijsen 2009 |  |  |  |  | √ |  |  |  |  |
| Pryce 2007 |  |  |  |  | √ |  | √ |  |  |
| Quinlan 2009, 2011 |  |  | √ |  |  |  |  |  |  |
| Schmidt 2019 |  |  | √ |  |  |  |  |  |  |
| Smoot 2010 |  |  | √ |  |  |  |  |  |  |
| So 2020 |  |  |  |  |  |  |  |  |  |
| Spelten 2003 |  |  |  |  | √ |  |  |  |  |
| Steiner 2008 |  |  |  |  |  |  | √ |  |  |
| Syrjala 2004 |  |  |  |  |  |  |  |  |  |
| Tamminga 2016 |  |  |  |  |  |  |  |  |  |
| Tevaarwerk 2013 | √ |  |  |  | √ | √ | √ |  |  |
| Van Muijen 2014, 2017, 2019 |  |  |  |  |  |  |  |  |  |
| Vartanian 2006 |  |  |  |  |  |  | √ |  |  |
| Verdonck-de Leeuw 2010 | √ |  |  |  | √ |  | √ |  | √ |
| Von Ah 2017, 2018 |  |  |  |  | √ |  |  |  |  |
| Wolvers 2019 |  |  |  |  |  |  |  |  |  |
| Yoo 2013 |  |  |  |  | √ |  | √ |  |  |
| Zanville 2016 |  |  |  |  |  | √ |  |  |  |
| Zeng 2017 |  |  |  |  | √ |  |  |  |  |
| Zomkowski 2020 |  | √ | √ |  | √ |  | √ |  |  |

| Study | Gastrointestinal | | | | | | Psychological | | |
| --- | --- | --- | --- | --- | --- | --- | --- | --- | --- |
|  | Anorexia | Weight gain | Weight loss | Nausea/ Vomiting | Diarrhea/ ↑Bowel urgency | Constipation | Anxiety | Depression | Body image issues |
| Agarwal 2017 | √ | √ | √ | √ | √ | √ |  |  |  |
| Alleaume 2018 |  |  |  |  |  |  |  |  |  |
| Azarkish 2015 |  |  |  |  |  |  |  |  |  |
| Back 2019 |  |  |  |  |  |  |  |  |  |
| Balak 2008 |  |  |  |  |  |  |  |  |  |
| Behringer 2016 |  |  |  |  |  |  |  |  |  |
| Bennett 2018 |  |  |  |  | √ |  |  |  |  |
| Boscher 2020 |  |  |  |  |  |  |  |  |  |
| Boyages 2016 |  |  |  |  |  |  |  |  |  |
| Brick 2019 |  |  |  |  |  |  | √ | √ |  |
| Calvio 2010 |  |  |  |  |  |  | √ | √ |  |
| Check 2019 | √ |  |  | √ |  | √ |  | √ |  |
| Chen 2012 |  |  |  |  |  |  |  |  | √ |
| Chen 2019 |  |  |  |  |  |  | √ | √ | √ |
| Cheng 2016 |  |  |  |  |  |  | √ | √ |  |
| Cheng 2018 |  |  |  |  |  |  |  |  |  |
| Cheville 2011 |  |  |  |  |  |  |  |  |  |
| Cooper 2013 | √ |  |  | √ | √ | √ | √ | √ |  |
| Cox-Martin 2020 |  |  |  |  |  |  |  |  |  |
| Dahl 2015, 2016 |  |  |  |  |  |  |  |  |  |
| Dahl 2019 |  |  |  |  |  |  | √ | √ |  |
| Dahl 2020 |  |  |  |  |  |  |  | √ |  |
| Dieluweit 2011 |  |  |  |  |  |  |  |  |  |
| Dorland 2018 |  |  |  |  |  |  |  | √ |  |
| Duijts 2017 | √ |  |  | √ | √ | √ |  | √ |  |
| Dumas 2020 |  |  |  |  |  |  | √ | √ |  |
| Dunberger 2013 |  |  |  |  |  |  |  |  |  |
| Ekenga 2019 |  |  |  |  |  |  |  | √ |  |
| Fantoni 2010 |  |  |  |  |  |  | √ |  |  |
| Gudbergsson 2008 |  |  |  |  |  |  | √ | √ |  |
| Hansen 2008 |  |  |  |  |  |  | √ | √ |  |
| Ho 2018 |  |  |  |  |  |  | √ | √ |  |
| Kenzik 2015 |  |  |  |  |  |  |  |  |  |
| Kerns 2020 |  |  |  |  |  |  |  |  |  |
| Kim 2014 | √ |  |  | √ | √ | √ |  |  |  |
| Kiserud 2015 |  |  |  |  |  |  |  |  |  |
| Kiserud 2016 |  |  |  |  |  |  | √ | √ |  |
| Koch 2015 |  |  |  |  |  |  |  | √ |  |
| Landeiro 2018 |  |  |  |  |  |  |  | √ |  |
| Lavigne 2008 |  |  |  |  |  |  |  | √ |  |
| Lee 2017 | √ |  |  | √ | √ | √ |  |  | √ |
| Lindbohm 2014 |  |  |  |  |  |  | √ | √ |  |
| Magyari 2017 |  |  |  |  |  |  | √ | √ |  |
| Mehnert 2017 |  |  |  |  |  |  |  | √ |  |
| Murray 2019 |  |  |  |  |  |  |  |  |  |
| Musti 2018 |  |  |  |  |  |  |  |  |  |
| Nakamura 2017 |  |  |  |  |  |  |  |  |  |
| Ng 2020 |  |  |  |  |  |  |  |  |  |
| Nieuwenhuijsen 2009 |  |  |  |  |  |  |  | √ |  |
| Pryce 2007 |  |  |  | √ |  |  | √ | √ | √ |
| Quinlan 2009, 2011 |  |  |  |  |  |  |  |  |  |
| Schmidt 2019 |  |  |  |  |  |  |  | √ |  |
| Smoot 2010 |  |  |  |  |  |  |  |  |  |
| So 2020 |  |  |  |  |  |  | √ | √ |  |
| Spelten 2003 |  |  |  |  |  |  |  | √ |  |
| Steiner 2008 |  |  |  | √ |  |  | √ | √ |  |
| Syrjala 2004 |  |  |  |  |  |  |  | √ |  |
| Tamminga 2016 |  |  |  |  |  |  | √ | √ |  |
| Tevaarwerk 2013 | √ |  |  | √ | √ | √ |  | √ | √ |
| Van Muijen 2014, 2017, 2019 |  |  |  |  |  |  |  | √ |  |
| Vartanian 2006 |  |  |  |  |  |  |  |  |  |
| Verdonck-de Leeuw 2010 | √ |  |  | √ | √ | √ | √ | √ |  |
| Von Ah 2017, 2018 |  |  |  |  |  |  |  |  |  |
| Wolvers 2019 |  |  |  |  |  |  |  |  |  |
| Yoo 2013 | √ |  |  | √ | √ | √ | √ | √ |  |
| Zanville 2016 |  |  |  |  |  |  |  |  |  |
| Zeng 2017 |  |  |  |  |  |  | √ | √ |  |
| Zomkowski 2020 |  |  |  |  |  |  |  |  | √ |

| Study | Respiratory | | Fatigue/Sleep-related | | | Others | | | |
| --- | --- | --- | --- | --- | --- | --- | --- | --- | --- |
|  | Coughing | Dyspnea | Fatigue | Insomnia | Drowsiness | Hot flashes | Rash | Sexual dysfunction | Urinary problems |
| Agarwal 2017 | √ | √ | √ | √ |  |  |  |  |  |
| Alleaume 2018 |  |  | √ |  |  |  |  |  |  |
| Azarkish 2015 |  |  |  |  |  |  |  |  |  |
| Back 2019 |  |  |  |  |  |  |  |  |  |
| Balak 2008 |  |  | √ |  |  |  |  |  |  |
| Behringer 2016 |  |  | √ |  |  |  |  |  |  |
| Bennett 2018 |  |  |  |  |  |  |  |  | √ |
| Boscher 2020 |  |  |  |  |  |  |  |  |  |
| Boyages 2016 |  |  |  |  |  |  |  |  |  |
| Brick 2019 |  |  | √ |  |  |  |  |  |  |
| Calvio 2010 |  |  | √ |  |  |  |  |  |  |
| Check 2019 | √ | √ | √ | √ | √ |  | √ |  |  |
| Chen 2012 |  |  |  |  |  |  |  |  |  |
| Chen 2019 |  |  |  |  |  |  |  |  |  |
| Cheng 2016 |  |  |  |  |  |  |  |  |  |
| Cheng 2018 |  |  |  |  |  |  |  |  |  |
| Cheville 2011 | √ | √ | √ |  |  |  |  |  |  |
| Cooper 2013 |  | √ | √ | √ |  |  |  |  |  |
| Cox-Martin 2020 |  |  |  |  |  |  |  |  |  |
| Dahl 2015, 2016 |  |  | √ |  |  |  |  |  | √ |
| Dahl 2019 |  |  | √ |  |  |  |  |  |  |
| Dahl 2020 |  |  | √ | √ |  |  |  |  |  |
| Dieluweit 2011 |  |  |  |  |  |  |  |  |  |
| Dorland 2018 |  |  | √ |  |  |  |  |  |  |
| Duijts 2017 |  | √ | √ | √ |  |  |  |  |  |
| Dumas 2020 |  |  | √ |  |  |  |  |  |  |
| Dunberger 2013 |  |  |  |  |  |  |  |  |  |
| Ekenga 2019 |  |  | √ |  |  |  |  |  |  |
| Fantoni 2010 |  |  | √ |  |  |  |  |  |  |
| Gudbergsson 2008 |  |  |  |  |  |  |  |  |  |
| Hansen 2008 |  |  | √ | √ |  |  |  |  |  |
| Ho 2018 |  |  | √ |  |  |  |  |  |  |
| Kenzik 2015 |  |  |  |  |  |  |  |  |  |
| Kerns 2020 |  |  |  |  |  |  |  |  |  |
| Kim 2014 |  | √ | √ | √ |  |  |  |  |  |
| Kiserud 2015 |  |  | √ |  |  |  |  |  |  |
| Kiserud 2016 |  |  | √ |  |  |  |  |  |  |
| Koch 2015 |  |  |  |  |  |  |  |  |  |
| Landeiro 2018 |  |  |  |  |  |  |  |  |  |
| Lavigne 2008 |  |  | √ |  |  | √ |  |  |  |
| Lee 2017 |  | √ | √ | √ |  |  |  | √ |  |
| Lindbohm 2014 |  |  | √ |  |  |  |  |  |  |
| Magyari 2017 |  |  |  |  |  |  |  |  |  |
| Mehnert 2017 |  |  |  |  |  |  |  |  |  |
| Murray 2019 |  |  | √ |  |  |  |  |  |  |
| Musti 2018 |  |  |  |  |  |  |  |  |  |
| Nakamura 2017 |  |  |  |  |  |  |  |  | √ |
| Ng 2020 |  |  |  |  |  |  |  |  |  |
| Nieuwenhuijsen 2009 |  |  | √ |  |  |  |  |  |  |
| Pryce 2007 |  |  | √ |  |  |  |  |  |  |
| Quinlan 2009, 2011 |  |  |  |  |  |  |  |  |  |
| Schmidt 2019 |  |  | √ |  |  |  |  |  |  |
| Smoot 2010 |  |  |  |  |  |  |  |  |  |
| So 2020 |  |  |  |  |  |  |  |  |  |
| Spelten 2003 |  |  | √ | √ |  |  |  |  |  |
| Steiner 2008 |  |  | √ | √ |  |  |  |  |  |
| Syrjala 2004 |  |  |  |  |  |  |  |  |  |
| Tamminga 2016 |  |  | √ |  |  |  |  |  |  |
| Tevaarwerk 2013 | √ | √ | √ | √ | √ |  | √ |  |  |
| Van Muijen 2014, 2017, 2019 |  |  | √ |  |  |  |  |  |  |
| Vartanian 2006 |  |  |  |  |  |  |  |  |  |
| Verdonck-de Leeuw 2010 | √ | √ | √ | √ |  |  |  |  |  |
| Von Ah 2017, 2018 |  |  |  |  |  |  |  |  |  |
| Wolvers 2019 |  |  | √ |  |  |  |  |  |  |
| Yoo 2013 |  | √ | √ | √ |  |  |  |  |  |
| Zanville 2016 |  |  |  |  |  |  |  |  |  |
| Zeng 2017 |  |  |  |  |  |  |  |  |  |
| Zomkowski 2020 |  |  | √ |  |  |  |  | √ |  |

**Supplementary Material 5B: Summary of work outcomes evaluated by studies reviewed**

| Study | Absenteeism | Work status | | Presenteeism | | Others | | | |
| --- | --- | --- | --- | --- | --- | --- | --- | --- | --- |
|  | Absenteeism | Employment/RTW | Early retirement or disability | Productivity | Work ability | Work changes | Occupational roles limitations | Income | Working hours |
| Agarwal 2017 |  | √ |  |  |  |  |  |  |  |
| Alleaume 2018 |  | √ |  |  |  |  |  |  |  |
| Azarkish 2015 |  | √ |  |  |  |  |  |  |  |
| Back 2019 |  | √ |  |  |  |  |  |  |  |
| Balak 2008 |  | √ |  |  |  |  |  |  |  |
| Behringer 2016 |  | √ |  |  |  |  |  |  |  |
| Bennett 2018 |  | √ |  |  |  |  |  |  |  |
| Boscher 2020 |  | √ |  |  |  |  |  |  |  |
| Boyages 2016 |  |  |  |  |  | √ |  |  |  |
| Brick 2019 |  |  |  | √ |  |  |  |  |  |
| Calvio 2010 |  |  |  | √ |  |  |  |  |  |
| Check 2019 |  | √ |  |  |  |  |  |  |  |
| Chen 2012 |  | √ |  |  |  |  |  |  |  |
| Chen 2019 |  | √ |  |  |  |  |  |  |  |
| Cheng 2016 |  |  |  | √ |  |  |  |  |  |
| Cheng 2018 |  |  |  | √ |  |  |  |  |  |
| Cheville 2011 |  | √ |  |  |  |  |  |  |  |
| Cooper 2013 |  | √ |  |  |  |  |  |  |  |
| Cox-Martin 2020 |  | √ |  |  |  |  |  |  |  |
| Dahl 2015, 2016 |  | √ |  |  | √ |  |  |  |  |
| Dahl 2019 |  | √ |  |  | √ |  |  |  |  |
| Dahl 2020 |  |  | √ |  |  |  |  |  |  |
| Dieluweit 2011 |  | √ |  |  |  |  |  |  |  |
| Dorland 2018 |  |  |  |  | √ |  |  |  |  |
| Duijts 2017 |  | √ |  |  |  |  |  |  |  |
| Dumas 2020 |  | √ |  |  |  |  |  |  |  |
| Dunberger 2013 |  | √ |  |  |  |  |  |  |  |
| Ekenga 2019 |  | √ |  |  |  |  |  |  |  |
| Fantoni 2010 |  | √ |  |  |  |  |  |  |  |
| Gudbergsson 2008 |  |  |  |  |  | √ |  |  |  |
| Hansen 2008 |  |  |  | √ |  |  |  |  |  |
| Ho 2018 |  |  |  |  | √ |  |  |  |  |
| Kenzik 2015 |  | √ |  |  |  |  |  |  |  |
| Kerns 2020 |  | √ | √ |  |  |  |  |  |  |
| Kim 2014 |  | √ |  |  |  |  |  |  |  |
| Kiserud 2015 |  | √ |  |  |  |  |  |  |  |
| Kiserud 2016 |  | √ |  |  |  |  |  |  |  |
| Koch 2015 |  | √ |  |  |  |  |  | √ | √ |
| Landeiro 2018 |  | √ |  |  |  |  |  |  |  |
| Lavigne 2008 | √ |  |  | √ |  |  |  |  |  |
| Lee 2017 |  | √ |  |  |  |  |  |  |  |
| Lindbohm 2014 |  | √ |  |  |  |  |  |  |  |
| Magyari 2017 |  | √ |  |  |  |  |  |  |  |
| Mehnert 2017 |  |  | √ |  |  |  |  |  |  |
| Murray 2019 |  | √ |  |  |  |  |  |  |  |
| Musti 2018 |  |  |  |  | √ |  |  |  |  |
| Nakamura 2017 |  | √ |  |  |  |  |  |  |  |
| Ng 2020 |  | √ |  |  |  |  |  |  |  |
| Nieuwenhuijsen 2009 |  | √ |  |  | √ |  |  |  |  |
| Pryce 2007 |  | √ |  |  |  |  |  |  |  |
| Quinlan 2009, 2011 |  |  |  | √ |  |  |  |  |  |
| Schmidt 2019 |  | √ |  |  |  |  |  |  |  |
| Smoot 2010 |  | √ |  |  |  |  |  |  |  |
| So 2020 |  | √ |  |  |  |  |  |  |  |
| Spelten 2003 |  | √ |  |  |  |  |  |  |  |
| Steiner 2008 |  | √ |  |  |  | √ | √ |  |  |
| Syrjala 2004 |  | √ |  |  |  |  |  |  |  |
| Tamminga 2016 |  | √ |  |  |  |  |  |  |  |
| Tevaarwerk 2013 |  | √ |  |  |  |  |  |  |  |
| Van Muijen 2014, 2017, 2019 |  |  | √ |  | √ |  |  |  |  |
| Vartanian 2006 |  |  | √ |  |  |  |  |  |  |
| Verdonck-de Leeuw 2010 |  | √ |  |  |  |  |  |  |  |
| Von Ah 2017, 2018 |  |  |  | √ | √ |  |  |  |  |
| Wolvers 2019 |  |  |  |  | √ |  |  |  |  |
| Yoo 2013 |  | √ |  |  |  |  |  |  |  |
| Zanville 2016 |  |  |  |  | √ |  |  |  |  |
| Zeng 2017 |  |  |  | √ |  |  |  |  |  |
| Zomkowski 2020 |  | √ |  |  |  |  |  |  |  |

**Supplementary Material 6: Summary of findings from studies reviewed**

**Localised: Oral dysfunction (Chewing)**

| Study | Cancer | Time since dx | Time since tx | Symptom | | Work outcome | | Effect measure | Estimate | Spread | Notes |
| --- | --- | --- | --- | --- | --- | --- | --- | --- | --- | --- | --- |
|  |  |  |  | Measurement tool | Variable | Measurement tool | Variable |  |  |  |  |
| **Work status (Employment/RTW)** | | | | | | | | | | | |
| Check 2019 | H&N (Oropharyngeal squamous cell carcinoma) | 1 to 22 yr (Median = 6 yr) | >1 yr | MD Anderson Symptom Inventory-Head and Neck (MDASI-HN) module | Cont | Single question on employment | Cat | Mean scores | 2.3 (emp) vs 3.4 (unemp) | SD = 2.6 (emp) vs 3 (unemp) | p < 0.001. MDASI: higher scores indicate higher level of sx burden. |
| Chen 2019 | H&N (Oral cavity cancer) | NR | >6 m (Mean = 33 m) | University of Washington Quality of Life Questionnaire (UW-QoL) item | Cont | Single question on employment | Cat | Mean scores | 53.54 (RTW) vs 28.59 (no RTW) | SD = 28.02 (RTW) vs 26.21 (no RTW) | p = 0.001. UWQOL: higher scores indicate lower levels of sx burden. |

**Localised: Oral dysfunction (Dry mouth)**

| Study | Cancer | Time since dx | Time since tx | Symptom | | Work outcome | | Effect measure | Estimate | Spread | Notes |
| --- | --- | --- | --- | --- | --- | --- | --- | --- | --- | --- | --- |
|  |  |  |  | Measurement tool | Variable | Measurement tool | Variable |  |  |  |  |
| **Work status (Employment/RTW)** | | | | | | | | | | | |
| Agarwal 2017 | H&N | NR | >6 m (Mean = 19 m) | EORTC-QLQ-H&N 35 single item | Cont | Single question on employment | Cat | Mean scores | 30.8 (RTW) vs 25.5 (non-RTW) | SD = 28.5 (RTW) vs 31.2 (non-RTW) | p = 0.091. EORTC: higher scores indicate higher level of sx burden. |
| Check 2019 | H&N (Oropharyngeal squamous cell carcinoma) | 1 to 22 yr (Median = 6 yr) | >1 yr | MD Anderson Symptom Inventory-Head and Neck (MDASI-HN) module | Cont | Single question on employment | Cat | Mean scores | 3.5 (emp) vs 4.3 (unemp) | SD = 2.7 (emp) vs 2.9 (unemp) | p = 0.001. MDASI: higher scores indicate higher level of sx burden. |
| Chen 2019 | H&N (Oral cavity cancer) | NR | >6 m (Mean = 33 m) | University of Washington Quality of Life Questionnaire (UW-QoL) item | Cont | Single question on employment | Cat | Mean scores | 80.63 (RTW) vs 82.82 (no RTW) | SD = 31.68 (RTW) vs 30.83 (no RTW) | p = 0.646. UWQOL: higher scores indicate lower levels of sx burden. |
| Tevaarwerk 2013 | Various (Breast 74%) | >6 m (6 to 12 m: 13%; 12 to 24 m: 24%; >24 m: 63%) | NR | MD Anderson Symptom Inventory-Modified (MDASI-ECOG) | Cat (<4, >=5) | Two questions on current employment status and if changes occurred due to illness | Cat (Unemployment/Non-RTW) | OR | 2.6 | 95% CI: 1.1 to 6.2 | MDASI: threshold based on MDASI recommendations with >=5 indicating severe level. |
| Verdonck-de Leeuw 2010 | H&N | NR | >2 yr | EORTC-QLQ-H&N 35 single item | Cont | Single question on employment change | Cat (Return to same work, changed work, no RTW) | Mean scores | 32.14 (same) vs 35.42 (changed) vs 70.83 (no RTW) | SD = 30.74 (same) vs 28.46 (changed) vs 37.53 (no RTW) | p < 0.05. EORTC: higher scores indicate higher level of sx burden. |

**Localised: Oral dysfunction (Opening)**

| Study | Cancer | Time since dx | Time since tx | Symptom | | Work outcome | | Effect measure | Estimate | Spread | Notes |
| --- | --- | --- | --- | --- | --- | --- | --- | --- | --- | --- | --- |
|  |  |  |  | Measurement tool | Variable | Measurement tool | Variable |  |  |  |  |
| **Work status (Employment/RTW)** | | | | | | | | | | | |
| Agarwal 2017 | H&N | NR | >6 m (Mean = 19 m) | EORTC-QLQ-H&N 35 single item | Cont | Single question on employment | Cat | Mean scores | 29 (RTW) vs 29 (non-RTW) | SD = 28 (RTW) vs 27.5 (non-RTW) | p = 0.341. EORTC: higher scores indicate higher level of sx burden. |
| Verdonck-de Leeuw 2010 | H&N | NR | >2 yr | EORTC-QLQ-H&N 35 single item | Cont | Single question on employment change | Cat (Return to same work, changed work, no RTW) | Mean scores | 15.48 (same) vs 35.12 (changed) vs 58.33 (no RTW) | SD = 26.42 (same) vs 39.38 (changed) vs 34.5 (no RTW) | p < 0.05. EORTC: higher scores indicate higher level of sx burden. |

**Localised: Oral dysfunction (Local pain)**

| Study | Cancer | Time since dx | Time since tx | Symptom | | Work outcome | | Effect measure | Estimate | Spread | Notes |
| --- | --- | --- | --- | --- | --- | --- | --- | --- | --- | --- | --- |
|  |  |  |  | Measurement tool | Variable | Measurement tool | Variable |  |  |  |  |
| **Work status (Employment/RTW)** | | | | | | | | | | | |
| Agarwal 2017 | H&N | NR | >6 m (Mean = 19 m) | EORTC-QLQ-H&N 35 subscale | Cont | Single question on employment | Cat | Mean scores | 12.9 (RTW) vs 9.2 (non-RTW) | SD = 19.6 (RTW) vs 17.1 (non-RTW) | p = 0.242. EORTC: higher scores indicate higher level of sx burden. |
| Check 2019 | H&N (Oropharyngeal squamous cell carcinoma) | 1 to 22 yr (Median = 6 yr) | >1 yr | MD Anderson Symptom Inventory-Head and Neck (MDASI-HN) module | Cont | Single question on employment | Cat | Mean scores | 0.5 (emp) vs 0.5 (unemp) | SD = 1.3 (emp) vs 1.3 (unemp) | p = 0.6. MDASI: higher scores indicate higher level of sx burden. |
| Tevaarwerk 2013 | Various (Breast 74%) | >6 m (6 to 12 m: 13%; 12 to 24 m: 24%; >24 m: 63%) | NR | MD Anderson Symptom Inventory-Modified (MDASI-ECOG) | Cat (<4, >=5) | Two questions on current employment status and if changes occurred due to illness | Cat (Unemployment/Non-RTW) | OR | - | - | Not included in analysis as less than 1-% reported severe level. MDASI: threshold based on MDASI recommendations with >=5 indicating severe level. |
| Verdonck-de Leeuw 2010 | H&N | NR | >2 yr | EORTC-QLQ-H&N 35 subscale | Cont | Single question on employment change | Cat (Return to same work, changed work, no RTW) | Mean scores | 14.88 (same) vs 19.27 (changed) vs 32.39 (no RTW) | SD = 19.29 (same) vs 22.71 (changed) vs 22.02 (no RTW) | EORTC: higher scores indicate higher level of sx burden. |

**Localised: Oral dysfunction (Speaking)**

| Study | Cancer | Time since dx | Time since tx | Symptom | | Work outcome | | Effect measure | Estimate | Spread | Notes |
| --- | --- | --- | --- | --- | --- | --- | --- | --- | --- | --- | --- |
|  |  |  |  | Measurement tool | Variable | Measurement tool | Variable |  |  |  |  |
| **Work status (Employment/RTW)** | | | | | | | | | | | |
| Agarwal 2017 | H&N | NR | >6 m (Mean = 19 m) | EORTC-QLQ-H&N 35 subscale | Cont | Single question on employment | Cat | Mean scores | 19.9 (RTW) vs 19.4 (non-RTW) | SD = 19.8 (RTW) vs 23.2 (non-RTW) | p = 0.662. EORTC: higher scores indicate higher level of sx burden. |
| Check 2019 | H&N (Oropharyngeal squamous cell carcinoma) | 1 to 22 yr (Median = 6 yr) | >1 yr | MD Anderson Symptom Inventory-Head and Neck (MDASI-HN) module | Cont | Single question on employment | Cat | Mean scores | 1.2 (emp) vs 2.1 (unemp) | SD = 2.2 (emp) vs 2.7 (unemp) | p < 0.001. MDASI: higher scores indicate higher level of sx burden. |
| Chen 2019 | H&N (Oral cavity cancer) | NR | >6 m (Mean = 33 m) | University of Washington Quality of Life Questionnaire (UW-QoL) item | Cont | Single question on employment | Cat | Mean scores | 71.35 (RTW) vs 68.08 (no RTW) | SD = 19.76 (RTW) vs 27.1 (no RTW) | p = 0.374. UWQOL: higher scores indicate lower levels of sx burden. |
| Verdonck-de Leeuw 2010 | H&N | NR | >2 yr | EORTC-QLQ-H&N 35 subscale | Cont | Single question on employment change | Cat (Return to same work, changed work, no RTW) | Mean scores | 6.74 (same) vs 17.36 (changed) vs 19.44 (no RTW) | SD = 11.84 (same) vs 18.13 (changed) vs 22.81 (no RTW) | EORTC: higher scores indicate higher level of sx burden. |

**Localised: Oral dysfunction (Sticky saliva)**

| Study | Cancer | Time since dx | Time since tx | Symptom | | Work outcome | | Effect measure | Estimate | Spread | Notes |
| --- | --- | --- | --- | --- | --- | --- | --- | --- | --- | --- | --- |
|  |  |  |  | Measurement tool | Variable | Measurement tool | Variable |  |  |  |  |
| **Work status (Employment/RTW)** | | | | | | | | | | | |
| Agarwal 2017 | H&N | NR | >6 m (Mean = 19 m) | EORTC-QLQ-H&N 35 single item | Cont | Single question on employment | Cat | Mean scores | 27.8 (RTW) vs 16.6 (non-RTW) | SD = 31.1 (RTW) vs 23.9 (non-RTW) | p = 0.004. EORTC: higher scores indicate higher level of sx burden. |
| Check 2019 | H&N (Oropharyngeal squamous cell carcinoma) | 1 to 22 yr (Median = 6 yr) | >1 yr | MD Anderson Symptom Inventory-Head and Neck (MDASI-HN) module | Cont | Single question on employment | Cat | Mean scores | 2 (emp) vs 2.8 (unemp) | SD = 2.6 (emp) vs 2.9 (unemp) | p = 0.001. MDASI: higher scores indicate higher level of sx burden. |
| Verdonck-de Leeuw 2010 | H&N | NR | >2 yr | EORTC-QLQ-H&N 35 single item | Cont | Single question on employment change | Cat (Return to same work, changed work, no RTW) | Mean scores | 17.86 (same) vs 33.33 (changed) vs 58.33 (no RTW) | SD = 21.24 (same) vs 38.49 (changed) vs 38.83 (no RTW) | p < 0.05. EORTC: higher scores indicate higher level of sx burden. |

**Localised: Oral dysfunction (Swallowing)**

| Study | Cancer | Time since dx | Time since tx | Symptom | | Work outcome | | Effect measure | Estimate | Spread | Notes |
| --- | --- | --- | --- | --- | --- | --- | --- | --- | --- | --- | --- |
|  |  |  |  | Measurement tool | Variable | Measurement tool | Variable |  |  |  |  |
| **Work status (Employment/RTW)** | | | | | | | | | | | |
| Agarwal 2017 | H&N | NR | >6 m (Mean = 19 m) | EORTC-QLQ-H&N 35 subscale | Cont | Single question on employment | Cat | Mean scores | 14.8 (RTW) vs 10.2 (non-RTW) | SD = 18.8 (RTW) vs 16.3 (non-RTW) | p = 0.061. EORTC: higher scores indicate higher level of sx burden. |
| Check 2019 | H&N (Oropharyngeal squamous cell carcinoma) | 1 to 22 yr (Median = 6 yr) | >1 yr | MD Anderson Symptom Inventory-Head and Neck (MDASI-HN) module | Cont | Single question on employment | Cat | Mean scores | 2.3 (emp) vs 3.4 (unemp) | SD = 2.6 (emp) vs 3 (unemp) | p < 0.001. MDASI: higher scores indicate higher level of sx burden. |
| Chen 2019 | H&N (Oral cavity cancer) | NR | >6 m (Mean = 33 m) | University of Washington Quality of Life Questionnaire (UW-QoL) item | Cont | Single question on employment | Cat | Mean scores | 71.04 (RTW) vs 55 (no RTW) | SD = 26.62 (RTW) vs 31.4 (no RTW) | p = 0.001. UWQOL: higher scores indicate lower levels of sx burden. |
| Verdonck-de Leeuw 2010 | H&N | NR | >2 yr | EORTC-QLQ-H&N 35 subscale | Cont | Single question on employment change | Cat (Return to same work, changed work, no RTW) | Mean scores | 9.03 (same) vs 19.27 (changed) vs 21.88 (no RTW) | SD = 14.39 (same) vs 25.59 (changed) vs 20.38 (no RTW) | EORTC: higher scores indicate higher level of sx burden. |

**Localised: Oral dysfunction (Teeth)**

| Study | Cancer | Time since dx | Time since tx | Symptom | | Work outcome | | Effect measure | Estimate | Spread | Notes |
| --- | --- | --- | --- | --- | --- | --- | --- | --- | --- | --- | --- |
|  |  |  |  | Measurement tool | Variable | Measurement tool | Variable |  |  |  |  |
| **Work status (Employment/RTW)** | | | | | | | | | | | |
| Agarwal 2017 | H&N | NR | >6 m (Mean = 19 m) | EORTC-QLQ-H&N 35 single item | Cont | Single question on employment | Cat | Mean scores | 22.7 (RTW) vs 18.4 (non-RTW) | SD = 29.1 (RTW) vs 26.7 (non-RTW) | p = 0.06. EORTC: higher scores indicate higher level of sx burden. |
| Check 2019 | H&N (Oropharyngeal squamous cell carcinoma) | 1 to 22 yr (Median = 6 yr) | >1 yr | MD Anderson Symptom Inventory-Head and Neck (MDASI-HN) module | Cont | Single question on employment | Cat | Mean scores | 1.3 (emp) vs 1.9 (unemp) | SD = 2.2 (emp) vs 2.9 (unemp) | p = 0.082. MDASI: higher scores indicate higher level of sx burden. |
| Verdonck-de Leeuw 2010 | H&N | NR | >2 yr | EORTC-QLQ-H&N 35 single item | Cont | Single question on employment change | Cat (Return to same work, changed work, no RTW) | Mean scores | 4.76 (same) vs 12.5 (changed) vs 41.67 (no RTW) | SD = 11.88 (same) vs 26.87 (changed) vs 38.83 (no RTW) | p < 0.05. EORTC: higher scores indicate higher level of sx burden. |

**Localised: Breast issues**

| Study | Cancer | Time since dx | Time since tx | Symptom | | Work outcome | | Effect measure | Estimate | Spread | Notes |
| --- | --- | --- | --- | --- | --- | --- | --- | --- | --- | --- | --- |
|  |  |  |  | Measurement tool | Variable | Measurement tool | Variable |  |  |  |  |
| **Work status (Employment/RTW)** | | | | | | | | | | | |
| Lee 2017 | Breast | Up to 36 m | NR | EORTC-QLQ-Breast 23 subscale | Cont | Single question on employment at baseline, 12m, 24m, 36m post dx | Cat (Employment/RTW) | OR | - | - | Symptom burden assessed at 12 and 24 months. Not included in final model based on univariate analysis, no estimates reported. |
| Dumas 2020 | Breast | 2 yr (Median = 23 m) | NR | EORTC-QLQ-Breast 23 subscale | Cat (Severe, ie. >=40) | Single question on employment | Cat (Unemployment/Non-RTW) | OR | 0.97 | 95% CI: 0.7 to 1.33 | Symptom burden assessed approximately 1 year prior. EORTC: threshold based on previous paper. |
| Zomkowski 2020 | Breast | [Mean time from surgery = 48 m] | >1 m | EORTC-QLQ-Breast 23 subscale | Cont | Single question on employment | Cat | Mean scores | 12.35 (RTW) vs 31.43 (no RTW) | SD = 16.24 (RTW) vs 26.51 (no RTW) | p = 0.001. EORTC: higher scores indicate higher level of sx burden. |
| **Work ability** | | | | | | | | | | | |
| Ho 2018 | Breast | >1 yr (Median = 4 yr) | NR | EORTC-QLQ-Breast 23 subscale | Cont | Work Ability Index (WAI) | Cont | Std beta | -1 | 95% CI:  -1.7 to -0.3 | EORTC scale: higher scores indicate higher level of sx burden; WAI: higher scores indicate greater ability. |

**Localised: Upper limb problems (General)**

| Study | Cancer | Time since dx | Time since tx | Symptom | | Work outcome | | Effect measure | Estimate | Spread | Notes |
| --- | --- | --- | --- | --- | --- | --- | --- | --- | --- | --- | --- |
|  |  |  |  | Measurement tool | Variable | Measurement tool | Variable |  |  |  |  |
| **Work status (Employment/RTW)** | | | | | | | | | | | |
| Balak 2008 | Breast | [2 yr since sick leave] | NR | Clinical diagnosis | Cat | Feedback to occupational health department | Time to event | HR | 0.48 | 95% CI: 0.23 to 0.98 | RTW is defined as partial RTW and time to RTW measured from first day of sick leave. Sx burden measured at time of RTW. Unexplained exclusion criteria of pre-existent chronic diseases that may lead to unemployment can potentially lead to selection bias. Differences in included and excluded pts may cause MNAR. |
| Balak 2008 | Breast | [2 yr since sick leave] | NR | Clinical diagnosis | Cat | Feedback to occupational health department | Time to event | HR | 0.63 | 95% CI: 0.31 to 1.26 | RTW is defined as full RTW and time to RTW measured from first day of sick leave. Sx burden measured at time of RTW. Unexplained exclusion criteria of pre-existent chronic diseases that may lead to unemployment can potentially lead to selection bias. Differences in included and excluded pts may cause MNAR. |
| Chen 2019 | H&N (Oral cavity cancer) | NR | >6 m (Mean = 33 m) | University of Washington Quality of Life Questionnaire (UW-QoL) item | Cont | Single question on employment | Cat | Mean scores | 67.6 (RTW) vs 56.41 (no RTW) | SD = 25.03 (RTW) vs 31.04 (no RTW) | p = 0.011. UWQOL: higher scores indicate lower levels of sx burden. |
| Dumas 2020 | Breast | 2 yr (Median = 23 m) | NR | EORTC-QLQ-Breast 23 subscale | Cat (Severe, ie. >=40) | Single question on employment | Cat (Unemployment/Non-RTW) | OR | 1.59 | 95% CI: 1.19 to 2.13 | Symptom burden assessed approximately 1 year prior. EORTC: threshold based on previous paper. |
| Lee 2017 | Breast | Up to 36 m | NR | EORTC-QLQ-Breast 23 subscale | Cont | Single question on employment at baseline, 12m, 24m, 36m post dx | Cat (Employment/RTW) | OR | - | - | Symptom burden assessed at 12 and 24 months. Not included in final model based on univariate analysis, no estimates reported. |
| Schmidt 2019 | Breast | NR | [>1 yr from surgery] Median = 247 days | EORTC-QLQ-Breast 23 subscale | Cont (Per unit of 25 units) | Several questions on working status and hours | Cat (Reduced hours or discontinuation of work) | OR | 2.4 | 95% CI: 1.2 to 4.9 | Symptom burden assessed at week 13 post surgery. EORTC: higher scores indicate higher level of sx burden. |
| Schmidt 2019 | Breast | NR | [>1 yr from surgery] Median = 247 days | EORTC-QLQ-Breast 23 subscale | Cont (Per unit of 25 units) | Several questions on working status and hours | Cat (Reduced hours or discontinuation of work) | OR | 1 | 95% CI: 0.5 to 1.9 | Symptom burden assessed at month 15 post surgery. EORTC: higher scores indicate higher level of sx burden. |
| Zomkowski 2020 | Breast | [Mean time from surgery = 48 m] | >1 m | EORTC-QLQ-Breast 23 subscale | Cont | Single question on employment | Cat | Mean scores | 36.21 (RTW) vs 40 (no RTW) | SD = 27.15 (RTW) vs 30.87 (no RTW) | p = 0.308. EORTC: higher scores indicate higher level of sx burden. |
| **Work ability** | | | | | | | | | | | |
| Ho 2018 | Breast | >1 yr (Median = 4 yr) | NR | EORTC-QLQ-Breast 23 subscale | Cont | Work Ability Index (WAI) | Cont | Std beta | - | - | Not included in final model based on stepwise backward selection. EORTC scale: higher scores indicate higher level of sx burden; WAI: higher scores indicate greater ability. |
| Musti 2018 | Breast | >2 yr | NR | Single question on therapy | Cat | Single question on work ability | Cat | OR | 1.43 | 95% CI: 0.84 to 2.44 |  |

**Localised: Upper limb problems (Lymphedema)**

| Study | Cancer | Time since dx | Time since tx | Symptom | | Work outcome | | Effect measure | Estimate | Spread | Notes |
| --- | --- | --- | --- | --- | --- | --- | --- | --- | --- | --- | --- |
|  |  |  |  | Measurement tool | Variable | Measurement tool | Variable |  |  |  |  |
| **Work status (Employment/RTW)** | | | | | | | | | | | |
| Azarkish 2015 | Breast | NR | 1 yr | Single question on symptom | Cat (No sx) | Single question on employment | Cat (Employment/RTW) | OR | 3.11 | 95% CI: 1.4 to 3.17 | Not specified if univariate or multivariable regression conducted. Error in estimates reported (ie. beta estimates reported as ORs. Exclusion criteria of not working in a governmental job not justified. |
| Fantoni 2010 | Breast | >2 yr | NR | Single question on symptom (No, yes - a little, yes - a lot) | Cat | Single question on employment | Cat (Employment/RTW) | OR | Yes, a little: 0.58; Yes, a lot: 0.09 | 95% CI: Yes, a little: 0.19 to 1.73 Yes, a lot: 0.02 to 0.30 | Sx burden measured at end of tx. |
| Fantoni 2010 | Breast | >2 yr | NR | Single question on symptom (No, yes - a little, yes - a lot) | Cat | Single question on employment | Time to event | RR | Yes, a little: 0.80; Yes, a lot: 0.33 | 95% CI: Yes, a little: 0.55 to 1.16 Yes, a lot: 0.15 to 0.72 | Time to RTW measured from start of tx. Sx burden measured at end of tx. |
| Landeiro 2018 | Breast | 24 m | NR | Single question on symptom | Cat | Single question on employment | Cat (Employment/RTW) | OR | - | - | Not included in final model based on stepwise forward selection, no estimates reported. |
| Smoot 2010 | Breast | NR | >6 m | Clinical diagnosis | Cat | Single question on paid employment | Cat | Proportion | 60% (Lymphedema) vs 58% (None) | - | p = 0.76 |
| **Work productivity** | | | | | | | | | | | |
| Quinlan 2009, 2011 | Breast | NR | [6 to 12 m from surgery] | Volume of affected arm | Cat (>5%) | Derived from several variables, including work hours, change in jobs | Cat (Productivity loss) | OR | - | - | Definition of presence of productivity loss not clearly described in manuscript or cited paper. Not included in final model based on stepwise forward selection, no estimates reported. |
| Quinlan 2009, 2011 | Breast | NR | [18 to 24 m from surgery] | Volume of affected arm | Cat (>5%) | Derived from several variables, including work hours, change in jobs | Cat (Productivity loss) | OR | - | - | Definition of presence of productivity loss not clearly described in manuscript or cited paper. Regression model not presented due to poor fit, no estimates reported. |
| Quinlan 2009, 2011 | Breast | NR | [30 to 36 m from surgery] | Volume of affected arm | Cat (>5%) | Derived from several variables, including work hours, change in jobs | Cat (Productivity loss) | OR | - | - | Definition of presence of productivity loss not clearly described in manuscript or cited paper. Regression model not presented due to poor fit, no estimates reported. |
| **Others (Changes in work)** | | | | | | | | | | | |
| Boyages 2016 | Breast | NR | >1 yr (<5 yr: 67%; >=5 yr: 33%) | Clinical diagnosis | Cat | Several questions on reduced work hours, stopping working, change in roles, change in employer and transition to self-employment | Cat | Proportion | 19% (Lymphedema) vs 51% (Breast ca) | - | Changes in work defined as reduced work hours, change in roles, change in employer or transition to self-employment. Demographic statistics based on full cohort, which includes subjects not analysed for change in employment conditions due to unemployment at baseline. More analysis conducted specifically within cohort of patients with lymphedema based on self-reported severity but not included in this review. |

**Localised: Upper limb problems (Local pain)**

| Study | Cancer | Time since dx | Time since tx | Symptom | | Work outcome | | Effect measure | Estimate | Spread | Notes |
| --- | --- | --- | --- | --- | --- | --- | --- | --- | --- | --- | --- |
|  |  |  |  | Measurement tool | Variable | Measurement tool | Variable |  |  |  |  |
| **Work productivity** | | | | | | | | | | | |
| Quinlan 2009, 2011 | Breast | NR | [6 to 12 m from surgery] | McGill Pain Questionnaire item | Cat (Presence of pain, ie. >0) | Derived from several variables, including work hours, change in jobs | Cat (Productivity loss) | OR | 2.388 | 95% CI: 1.081 to 5.275 | Definition of presence of productivity loss not clearly described in manuscript or cited paper. |
| Quinlan 2009, 2011 | Breast | NR | [18 to 24 m from surgery] | McGill Pain Questionnaire item | Cat (Presence of pain, ie. >0) | Derived from several variables, including work hours, change in jobs | Cat (Productivity loss) | OR | - | - | Definition of presence of productivity loss not clearly described in manuscript or cited paper. Regression model not presented due to poor fit, no estimates reported. |
| Quinlan 2009, 2011 | Breast | NR | [30 to 36 m from surgery] | McGill Pain Questionnaire item | Cat (Presence of pain, ie. >0) | Derived from several variables, including work hours, change in jobs | Cat (Productivity loss) | OR | 7.928 | 95% CI: 1.824 to 34.457 | Definition of presence of productivity loss not clearly described in manuscript or cited paper. |

**Localised: Upper limb problems (Range of motion)**

| Study | Cancer | Time since dx | Time since tx | Symptom | | Work outcome | | Effect measure | Estimate | Spread | Notes |
| --- | --- | --- | --- | --- | --- | --- | --- | --- | --- | --- | --- |
|  |  |  |  | Measurement tool | Variable | Measurement tool | Variable |  |  |  |  |
| **Work status (Employment/RTW)** | | | | | | | | | | | |
| Fantoni 2010 | Breast | >2 yr | NR | Single question on symptom (No, yes - a little, yes - a lot) | Cat | Single question on employment | Cat (Employment/RTW) | OR | - | - | Sx burden measured at end of tx. Not included in final model based on univariate analysis, no estimates reported. |
| Fantoni 2010 | Breast | >2 yr | NR | Single question on symptom (No, yes - a little, yes - a lot) | Cat | Single question on employment | Time to event | RR | - | - | Time to RTW measured from start of tx. Sx burden measured at end of tx. Not included in final model based on univariate analysis, no estimates reported. |
| **Work productivity** | | | | | | | | | | | |
| Quinlan 2009, 2011 | Breast | NR | [6 to 12 m from surgery] | Shoulder abduction and external rotation | Cat (<170° abduction and <80° rotation) | Derived from several variables, including work hours, change in jobs | Cat (Productivity loss) | OR | 3.119 | 95% CI: 1.454 to 6.689 | Definition of presence of productivity loss not clearly described in manuscript or cited paper. |
| Quinlan 2009, 2011 | Breast | NR | [18 to 24 m from surgery] | Shoulder abduction and external rotation | Cat (<170° abduction and <80° rotation | Derived from several variables, including work hours, change in jobs | Cat (Productivity loss) | OR | - | - | Definition of presence of productivity loss not clearly described in manuscript or cited paper. Regression model not presented due to poor fit, no estimates reported. |
| Quinlan 2009, 2011 | Breast | NR | [30 to 36 m from surgery] | Shoulder abduction and external rotation | Cat (<170° abduction and <80° rotation | Derived from several variables, including work hours, change in jobs | Cat (Productivity loss) | OR | 4.08 | 95% CI: 1.085 to 15.343 | Definition of presence of productivity loss not clearly described in manuscript or cited paper. |

**Localised: Upper limb problems (Strength)**

| Study | Cancer | Time since dx | Time since tx | Symptom | | Work outcome | | Effect measure | Estimate | Spread | Notes |
| --- | --- | --- | --- | --- | --- | --- | --- | --- | --- | --- | --- |
|  |  |  |  | Measurement tool | Variable | Measurement tool | Variable |  |  |  |  |
| **Work status (Employment/RTW)** | | | | | | | | | | | |
| Fantoni 2010 | Breast | >2 yr | NR | Single question on symptom (No, yes - a little, yes - a lot) | Cat | Single question on employment | Cat (Employment/RTW) | OR | - | - | Sx burden measured at end of tx. Not included in final model based on univariate analysis, no estimates reported. |
| Fantoni 2010 | Breast | >2 yr | NR | Single question on symptom (No, yes - a little, yes - a lot) | Cat | Single question on employment | Time to event | RR | - | - | Time to RTW measured from start of tx. Sx burden measured at end of tx. Not included in final model based on univariate analysis, no estimates reported. |

**Localised: Lower limb problems (Lymphedema)**

| Study | Cancer | Time since dx | Time since tx | Symptom | | Work outcome | | Effect measure | Estimate | Spread | Notes |
| --- | --- | --- | --- | --- | --- | --- | --- | --- | --- | --- | --- |
|  |  |  |  | Measurement tool | Variable | Measurement tool | Variable |  |  |  |  |
| **Work status (Employment/RTW)** | | | | | | | | | | | |
| Dahl 2020 | Gynae (Cervical) | Mean = 11 yr | NR | EORTC-QLQ-CX 24 single item | Cont | Single question on disability | Cat (Disability) | OR | 1 | 95% CI: 0.99 to 1.01 | Disability defined as being on disability pension. EORTC: higher scores indicate higher level of sx burden. |
| Dunberger 2013 | Gynae (Endometrial/cervical) | NR | Mean = 83 m | Single question on symptom (No, occasionally, at least once a month, at least once a week, at least three times a week, at least once a day) | Cat (At least occasionally) | Single question on employment | Cat (Student, umemployed, employed, housewife, on sick leave, disability pension, retired, not stated) | Proportion | Unemployed: 0.01 vs 0.03, employed: 0.38 vs 0.30) | - | p = 0.004 for omnibus test. However, no further comparisons between individual categories. |
| Nakamura 2017 | Gynae (Cervical) | NR | >1 yr (Mean = 4.5 yr) | Based on National Lymphedema Network criteria | Cat (Present, ie stage II and above) | Single question on employment | Cat (Non RTW) | Proportion | 57.1% (Fail) vs 17.4% (RTW) | - | Demographic statistics based on full cohort, which includes subjects not analysed for job change. Failure to RTW + RTW was not consistent with total N analysed. |

**Neurological: Cognitive impairment (General)**

| Study | Cancer | Time since dx | Time since tx | Symptom | | Work outcome | | Effect measure | Estimate | Spread | Notes |
| --- | --- | --- | --- | --- | --- | --- | --- | --- | --- | --- | --- |
|  |  |  |  | Measurement tool | Variable | Measurement tool | Variable |  |  |  |  |
| **Work status (Employment/RTW)** | | | | | | | | | | | |
| Agarwal 2017 | H&N | NR | >6 m (Mean = 19 m) | EORTC-QLQ-Core 30 subscale | Cont | Single question on employment | Cat | Mean scores | 77.5 (RTW) vs 72.3 (non-RTW) | SD = 20.5 (RTW) vs 21.6 (non-RTW) | p = 0.273. EORTC: higher scores indicate higher level of sx burden. |
| Back 2019 | Brain (Anaplastic glioma) | NR | [3 yr after RT] | National Cancer Institute Common Toxicity Criteria (CTC) | Cat (Grade 2 and above) | Single question on employment | Cat (Unemployment/Non-RTW) | OR | 1.8 | - | Symptom burden assessed at year 1 post RT. p value and SE not specified but statistically significant. |
| Boscher 2020 | Breast | NR | <=1 yr: 26.8%; 1-3 yr: 24.1%; >=3 yr: 49.1% | Single question on symptom | Cat | Single question on employment | Cat | Proportion | 58.5 (Cog complaints) vs 54.8% (None) | - | p < 0.001. |
| Cooper 2013 | Various (Urology: 32%; breast: 31%) | [Median time since start of sick leave = 4.7 m] | [Median time since start of tx = 3.9 m] | EORTC-QLQ-Core 30 subscale | Cont | Single question on employment at baseline, 6m and 12m | Time to event | HR | - | - | Time to RTW measured from cessaton of work. Sx covariate not included in final model based on stepwise forward selection if not stated. |
| Dieluweit 2011 | Various (Lymphoma 31%; malignant bone tumors 21%) | >5 yr (Mean = 13.7 yr) | NR | Study-specific questionnaire | Cat | Single question on employment | Cat (Employment/RTW) | OR | 0.55 | 95% CI: 0.34 to 0.89 |  |
| Duijts 2017 | Various (Breast 48%) | 2 to 4 yr | NR | EORTC-QLQ-Core 30 subscale | Cont | Single question on employment at 2, 3 and 4 years after dx | Cat (Sustained unemp, +change, -change, sustained emp) | Mean scores | 64.6 (sust unemp) vs 74.1 (+ch) vs 68.7 (-ch) vs 71.3 (sust emp) | - | p = 0.273. EORTC: higher scores indicate lower level of sx burden. |
| Duijts 2017 | Various (Breast 48%) | 2 to 4 yr | NR | EORTC-QLQ-Core 30 subscale | Cont | Single question on employment at 2, 3 and 4 years after dx | Cat (Sustained employment) | OR | - | - | Time lag GEE model used to correlate work continuation one year later with symptom burden. Not included in final model based on univariate analysis, no estimates reported. |
| Dumas 2020 | Breast | 2 yr (Median = 23 m) | NR | EORTC-QLQ-Fatigue 12 subscale | Cat (Severe, ie. >=40) | Single question on employment | Cat (Unemployment/Non-RTW) | OR | 1.02 | 95% CI: 0.7 to 1.49 | Symptom burden assessed approximately 1 year prior. EORTC: threshold based on previous paper. |
| Kim 2014 | Lung | Median = 4.1 yr | NR | EORTC-QLQ-Core 30 subscale | Cat (<33) | Single question on employment | Cat (Employment/RTW) | OR | - | - | Not included in final model based on stepwise selection. Univariate estimates = 2.00 (0.65-6.20). EORTC: threshold based on previous studies with <33 indicating problematic status. Demographic statistics based on full cohort, which includes subjects not analysed for RTW. |
| Kiserud 2016 | Hemato (Lymphoma) | Mean = 12.4 yr | Mean = 9.7 yr | Single question on symptom (None, some, much, very much) | Cat (None/some, much/very much) | Single question on employment | Cat (Unemployment/Non-RTW) | OR | - | - | Not included in final model based on clinical relevance/assessment of multicollinearity. Univariate estimates =1.99 (1.13-3.51). |
| Lee 2017 | Breast | Up to 36 m | NR | EORTC-QLQ-Core 30 subscale | Cont | Single question on employment at baseline, 12m, 24m, 36m post dx | Cat (Employment/RTW) | OR | - | - | Symptom burden assessed at 12 and 24 months. Not included in final model based on univariate analysis, no estimates reported. |
| Nieuwenhuijsen 2009 | Various (GI tract 31%; breast 20%) | [12 m after first day of sick leave] | NR | WMS Visual Reproduction, WAIS Digit Span, WAIS Digit Symbol, RBMT Story Recall, Stroop test, Trailmaking Test A, Trailmaking Test B, PASAT, GIT Word Fluency | Cat (Impaired, ie. z-score <=1.5 on >1 test or <=2 on >=1 test) | Single question on employment | Cat (Employment/RTW) | OR | 0.5 | 95% CI: 0.1 to 2.1 |  |
| Spelten 2003 | Various (Gynae 25%; breast 24%) | [18 m after sick leave] | NR | Cognitive Failures Questionnaire (CFQ) | Not specified | Single question on employment | Time to event | HR | - | - | Time to RTW measured from first day of sick leave. Symptom burden measured at 6 months after sick leave. Not included in final model based on univariate analysis and stepwise forward selection, no estimates reported. |
| Verdonck-de Leeuw 2010 | H&N | NR | >2 yr | EORTC-QLQ-Core 30 subscale | Cont | Single question on employment change | Cat (Return to same work, changed work, no RTW) | Mean scores | 85.71 (same) vs 88.54 (changed) vs 77.08 (no RTW) | SD = 14.13 (same) vs 15.77 (changed) vs 19.79 (no RTW) | EORTC: higher scores indicate lower level of sx burden. |
| Yoo 2013 | Gynae (Cervical) | NR | <5 yr: 29%; >=5 yr: 71% | EORTC-QLQ-Core 30 subscale | Cont | Single question on employment | Cat | Mean scores | 74.90 (emp) vs 72.41 (unemp) | - | GLM used to control for demographic and clinical covariates. EORTC: higher scores indicate lower level of symptom burden. |
| Zomkowski 2020 | Breast | [Mean time from surgery = 48 m] | >1 m | EORTC-QLQ-Core 30 subscale | Cont | Single question on employment | Cat | Mean scores | 75.31 (RTW) vs 61.91 (no RTW) | SD = 27.1 (RTW) vs 33.96 (no RTW) | p = 0.049. EORTC: higher scores indicate lower level of sx burden. |
| **Work ability** | | | | | | | | | | | |
| Dorland 2018 | Various (Breast 46%) | 6, 12 and 18 m | NR | Cognitive Symptom Checklist-Work (CSC-W) | Cont | Work Role Functioning Questionnaire (WRFQ) | Cont | Beta | -0.4 | 95% CI:  -0.5 to -0.31 | GEE used to control for time since RTW. CSC-W: higher scores indicate more impairment; WRFQ: higher scores indicate better ability. |
| Ho 2018 | Breast | >1 yr (Median = 4 yr) | NR | EORTC-QLQ-Core 30 subscale | Cont | Work Ability Index (WAI) | Cont | Std beta | - | - | Not included in final model based on stepwise backward selection, adjusted estimates from non-optimised full model = 0.6 (-0.2 to 1.4). EORTC: higher score indicates lower level of sx burden; WAI: higher scores indicate greater ability. |
| Nieuwenhuijsen 2009 | Various (GI tract 31%; breast 20%) | [12 m after first day of sick leave] | NR | WMS Visual Reproduction, WAIS Digit Span, WAIS Digit Symbol, RBMT Story Recall, Stroop test, Trailmaking Test A, Trailmaking Test B, PASAT, GIT Word Fluency | Cat (Impaired, ie. z-score <=1.5 on >1 test or <=2 on >=1 test) | Work Ability Index (WAI) - 1st qns only | Cont | Beta | -0.15 | 95% CI:  -2.5 to 0.8 | WAI: higher scores indicate greater working ability |
| Von Ah 2017, 2018 | Breast | NR | >12 m (Mean = 59.5 m) | Functional Assessment of Cancer Therapy–Cognitive (FACT-Cog) subscale | Cont | Work Ability Index (WAI) | Cont | Beta | -0.658 | - | PCI: higher scores indicate more impairment; WAI: higher scores indicate greater ability. |
| Von Ah 2017, 2018 | Breast | NR | >12 m (Mean = 59.5 m) | Functional Assessment of Cancer Therapy–Cognitive (FACT-Cog) subscale | Cont | Work Ability Index (WAI) | Cont | Beta | 0.472 | - | PCA: higher scores indicate lower impairment; WAI: higher scores indicate greater ability. |
| Von Ah 2017, 2018 | Breast | NR | >1 yr (Mean = 5 yr) | Attentional Function Index (AFI) | Cont | Work Ability Index (WAI) | Cont | Beta | 0.627 | - | p < 0.001. AFI: higher scores indicate higher level of attention; WAI: higher scores indicate better work ability. |
| **Work productivity** | | | | | | | | | | | |
| Cheng 2016 | Breast | NR | >6 m (Mean = 2.9 yr) | Cognitive Symptom Checklist-Work (CSC-W) | Cont | Work Limitations Questionnaire (WLQ) | Cont | Beta | 0.06 | 95% CI:  -0.22 to 0.33 | CSC: higher scores indicate higher level of sx burden; WLQ: score converted to productivity loss. |
| Cheng 2018 | Breast | NR | >2 yr (2-3 yr: 45%; >=3: 55%) | Cognitive Symptom Checklist-Work (CSC-W) | Cont | Work Limitations Questionnaire (WLQ) | Cont | Beta | -0.68 | - | Productivity defined as work output measured using WLQ. Study included non-cancer controls and analysis tested interaction between CSC-W and history of cancer, which was revealed to be statistically significant. CSC-W: higher scores indicate greater degree of impairment; WLQ: reversed to indicate higher output with higher scores. |
| Hansen 2008 | Breast | Mean = 3.8 yr | NR | Cognitive Symptom Checklist-Work (CSC-W) | Cont | Work Limitations Questionnaire (WLQ) | Cont | Beta | 0.04 | 95% CI:  -0.012 to 0.093 | CSC-W: higher scores indicate higher level of sx burden; WLQ: higher scores indicate lower productivity. Patients with chronic comorbid diseases were excluded but no specification of which diseases. |
| Von Ah 2017, 2018 | Breast | NR | >12 m (Mean = 59.5 m) | Functional Assessment of Cancer Therapy–Cognitive (FACT-Cog) subscale | Cont | Work Limitations Questionnaire (WLQ) subscale | Cont | Beta | 0.731 | - | Productivity defined as work output measured using WLQ. PCI: higher scores indicate higher impairment; WLQ work output: higher scores indicate poorer output. |
| Von Ah 2017, 2018 | Breast | NR | >12 m (Mean = 59.5 m) | Functional Assessment of Cancer Therapy–Cognitive (FACT-Cog) subscale | Cont | Work Limitations Questionnaire (WLQ) subscale | Cont | Beta | -0.494 | - | Productivity defined as work output measured using WLQ. PCA: higher scores indicate lower impairment; WLQ work output: higher scores indicate poorer output. |
| Zeng 2017 | Breast | NR | >2 yr (Mean = 3.8 yr) | Cognitive Symptom Checklist-Work (CSC-W) | Cont | Work Limitations Questionnaire (WLQ) | Cont | Beta | 0.002 | - | p = 0.001. No SE reported. CSC: higher scores indicate higher level of sx burden; WLQ: higher scores indicate lower productivity. |

**Neurological: Cognitive impairment (Attention)**

| Study | Cancer | Time since dx | Time since tx | Symptom | | Work outcome | | Effect measure | Estimate | Spread | Notes |
| --- | --- | --- | --- | --- | --- | --- | --- | --- | --- | --- | --- |
|  |  |  |  | Measurement tool | Variable | Measurement tool | Variable |  |  |  |  |
| **Work status (Employment/RTW)** | | | | | | | | | | | |
| Pryce 2007 | Various (Breast 48%) | NR | NR | Single question on symptom | Cat | Single question on employment | Cat (Employment/RTW) | OR | - | - | Not included in final model based on univariate analysis and stepwise backward selection, no estimates reported. |
| **Work productivity** | | | | | | | | | | | |
| Calvio 2010 | Breast | NR | 1 to 10 yr (Mean = 3 yr) | Cognitive Symptom Checklist (CSC) - Modified subscale | Cont | Work Limitations Questionnaire (WLQ) subscale | Cont | Beta | -0.01 | - | Productivity defined as work output measured using WLQ. p > 0.05. CSC: higher scores indicate poorer functioning; WLQ: higher scores indicate lower output. |
| Calvio 2010 | Breast | NR | 1 to 10 yr (Mean = 3 yr) | Central Nervous System Vital Signs (CNSVS) | Cont | Work Limitations Questionnaire (WLQ) subscale | Cont | Beta | 0.17 | - | Productivity defined as work output measured using WLQ. p > 0.05. CSC: higher scores indicate better functioning - use of raw or standardised scores in model not clarified; WLQ: higher scores indicate lower output. |

**Neurological: Cognitive impairment (Executive function)**

| Study | Cancer | Time since dx | Time since tx | Symptom | | Work outcome | | Effect measure | Estimate | Spread | Notes |
| --- | --- | --- | --- | --- | --- | --- | --- | --- | --- | --- | --- |
|  |  |  |  | Measurement tool | Variable | Measurement tool | Variable |  |  |  |  |
| **Work productivity** | | | | | | | | | | | |
| Brick 2019 | Breast | NR | [6 m after surgery and start of chemotherapy or endocrine therapy] | Patient Assessment of Own Functioning Inventory (PAOFI) subscale | Cont | Work Limitations Questionnaire (WLQ) | Cont | Correlation | -0.131 | - | p = 0.669. PAOFI: higher scores indicated higher level of sx; WLQ: higher scores indicate lower productivity. |
| Calvio 2010 | Breast | NR | 1 to 10 yr (Mean = 3 yr) | Cognitive Symptom Checklist (CSC) - Modified subscale | Cont | Work Limitations Questionnaire (WLQ) subscale | Cont | Beta | 0.26 | - | Productivity defined as work output measured using WLQ. p < 0.05. CSC: higher scores indicate poorer functioning; WLQ: higher scores indicate lower output. |
| Calvio 2010 | Breast | NR | 1 to 10 yr (Mean = 3 yr) | Central Nervous System Vital Signs (CNSVS) | Cont | Work Limitations Questionnaire (WLQ) subscale | Cont | Beta | -0.21 | - | Productivity defined as work output measured using WLQ. p > 0.05. CSC: higher scores indicate better functioning - use of raw or standardised scores in model not clarified; WLQ: higher scores indicate lower output. |

**Neurological: Cognitive impairment (Memory)**

| Study | Cancer | Time since dx | Time since tx | Symptom | | Work outcome | | Effect measure | Estimate | Spread | Notes |
| --- | --- | --- | --- | --- | --- | --- | --- | --- | --- | --- | --- |
|  |  |  |  | Measurement tool | Variable | Measurement tool | Variable |  |  |  |  |
| **Work status (Employment/RTW)** | | | | | | | | | | | |
| Check 2019 | H&N (Oropharyngeal squamous cell carcinoma) | 1 to 22 yr (Median = 6 yr) | >1 yr | MD Anderson Symptom Inventory-Head and Neck (MDASI-HN) module | Cont | Single question on employment | Cat | Mean scores | 1.6 (emp) vs 2.2 (unemp) | SD = 2.1 (emp) vs 2.5 (unemp) | p = 0.01. MDASI: higher scores indicate higher level of sx burden. |
| Tevaarwerk 2013 | Various (Breast 74%) | >6 m (6 to 12 m: 13%; 12 to 24 m: 24%; >24 m: 63%) | NR | MD Anderson Symptom Inventory-Modified (MDASI-ECOG) | Cat (<4, >=5) | Two questions on current employment status and if changes occurred due to illness | Cat (Unemployment/Non-RTW) | OR | - | - | Not included in final model based on stepwise forward selection, no estimates for final model reported. Univariate estimates = 4.6 (3.0-7.1). MDASI: threshold based on MDASI recommendations with >=5 indicating severe level. |
| **Work productivity** | | | | | | | | | | | |
| Brick 2019 | Breast | NR | [6 m after surgery and start of chemotherapy or endocrine therapy] | Patient Assessment of Own Functioning Inventory (PAOFI) subscale | Cont | Work Limitations Questionnaire (WLQ) | Cont | Correlation | - | - | Correlation not statistically significant > 0, no estimates reported. |
| Calvio 2010 | Breast | NR | 1 to 10 yr (Mean = 3 yr) | Cognitive Symptom Checklist (CSC) - Modified subscale | Cont | Work Limitations Questionnaire (WLQ) subscale | Cont | Beta | 0.29 | - | Productivity defined as work output measured using WLQ. p < 0.05. CSC: higher scores indicate poorer functioning; WLQ: higher scores indicate lower output. |
| Calvio 2010 | Breast | NR | 1 to 10 yr (Mean = 3 yr) | Central Nervous System Vital Signs (CNSVS) | Cont | Work Limitations Questionnaire (WLQ) subscale | Cont | Beta | 1.37 | - | Productivity defined as work output measured using WLQ. p > 0.05. CSC: higher scores indicate better functioning - use of raw or standardised scores in model not clarified; WLQ: higher scores indicate lower output. |

**Neurological: Cognitive impairment (Verbal fluency)**

| Study | Cancer | Time since dx | Time since tx | Symptom | | Work outcome | | Effect measure | Estimate | Spread | Notes |
| --- | --- | --- | --- | --- | --- | --- | --- | --- | --- | --- | --- |
|  |  |  |  | Measurement tool | Variable | Measurement tool | Variable |  |  |  |  |
| **Work productivity** | | | | | | | | | | | |
| Brick 2019 | Breast | NR | [6 m after surgery and start of chemotherapy or endocrine therapy] | Patient Assessment of Own Functioning Inventory (PAOFI) subscale | Cont | Work Limitations Questionnaire (WLQ) | Cont | Correlation | - | - | Correlation not statistically significant > 0, no estimates reported. |

**Neurological: Neuropathy**

| Study | Cancer | Time since dx | Time since tx | Symptom | | Work outcome | | Effect measure | Estimate | Spread | Notes |
| --- | --- | --- | --- | --- | --- | --- | --- | --- | --- | --- | --- |
|  |  |  |  | Measurement tool | Variable | Measurement tool | Variable |  |  |  |  |
| **Work status (Employment/RTW)** | | | | | | | | | | | |
| Brick 2019 | Breast | NR | [6 m after surgery and start of chemotherapy or endocrine therapy] | Patient Assessment of Own Functioning Inventory (PAOFI) subscale | Cont | Work Limitations Questionnaire (WLQ) | Cont | Correlation | - | - | Correlation not statistically significant > 0, no estimates reported. |
| Check 2019 | H&N (Oropharyngeal squamous cell carcinoma) | 1 to 22 yr (Median = 6 yr) | >1 yr | MD Anderson Symptom Inventory-Head and Neck (MDASI-HN) module | Cont | Single question on employment | Cat | Mean scores | 1.3 (emp) vs 1.6 (unemp) | SD = 2.2 (emp) vs 2.5 (unemp) | p = 0.2. MDASI: higher scores indicate higher level of sx burden. |
| Kerns 2020 | Testicular | NR | >1 yr (Median = 3.8 yr) | EORTC-QLQ-CIPN 20, Scale for Chemotherapy-Induced Long-Term Neurotoxicity (SCIN) | Cat (Grade 0, 1, 2, 3) | Single question on employment | Cat (Unemployment/Non-RTW) | OR | Grade 1: 1.60; Grade 2: 1.48; Grade 3: 2.44 | 95% CI: Grade 1: 0.95 to 2.70 Grade 2: 0.76 to 2.87 Grade 3: 1.28 to 4.62 | PROs: threshold not justified. |
| Tevaarwerk 2013 | Various (Breast 74%) | >6 m (6 to 12 m: 13%; 12 to 24 m: 24%; >24 m: 63%) | NR | MD Anderson Symptom Inventory-Modified (MDASI-ECOG) | Cat (<4, >=5) | Two questions on current employment status and if changes occurred due to illness | Cat (Unemployment/Non-RTW) | OR | - | - | Not included in final model based on stepwise forward selection, no estimates for final model reported. Univariate estimates = 4.7 (2.7-8.2). MDASI: threshold based on MDASI recommendations with >=5 indicating severe level. |
| **Work status (Early retirement/disability)** | | | | | | | | | | | |
| Kerns 2020 | Testicular | NR | >1 yr (Median = 3.8 yr) | EORTC-QLQ-CIPN 20, Scale for Chemotherapy-Induced Long-Term Neurotoxicity (SCIN) | Cat (Grade 0, 1, 2, 3) | Single question on disability | Cat (Disability) | OR | Grade 1: 1.23; Grade 2: 0.83; Grade 3: 2.89 | 95% CI: Grade 1: 0.43 to 3.54 Grade 2: 0.22 to 3.09 Grade 3: 1.01 to 8.26 | Disability defined as being on disability leave. PROs: threshold based on clinical grading. |
| **Work ability** | | | | | | | | | | | |
| Zanville 2016 | Breast | NR | >1 yr | Functional Assessment of Cancer Therapy Gynaecological Oncology Group (FACT/GOG) subscale | Cont | Functional Assessment of Cancer Therapy Gynaecological Oncology Group (FACT/GOG) subscale | Cont | Correlation | - | - | Different combinations of neuropathy sx found to be not significantly correlated to perceived work ability, no estimates reported. |

**Neurological: Pain**

| Study | Cancer | Time since dx | Time since tx | Symptom | | Work outcome | | Effect measure | Estimate | Spread | Notes |
| --- | --- | --- | --- | --- | --- | --- | --- | --- | --- | --- | --- |
|  |  |  |  | Measurement tool | Variable | Measurement tool | Variable |  |  |  |  |
| **Work status (Employment/RTW)** | | | | | | | | | | | |
| Agarwal 2017 | H&N | NR | >6 m (Mean = 19 m) | EORTC-QLQ-Core 30 subscale | Cont | Single question on employment | Cat | Mean scores | 15.1 (RTW) vs 12.4 (non-RTW) | SD = 24.3 (RTW) vs 18.5 (non-RTW) | p = 0.613. EORTC: higher scores indicate higher level of sx burden. |
| Alleaume 2018 | Various (Breast 58%) | 5 yr | NR | DN4 questionnaire | Cat (>=3) | Several questions on working status and hours | Cat (Employed without working time reduction, unemployed, employed with working time reduction) | OR | Unemployed: 2.57; with time reduction: 1.89 | 95% CI: Unemp: 1.70 to 3.88 With time reduction: 1.32 to 2.71 | CN4: threshold used based on previous studies, with >=3 indicating presence if chronic neuropathic pain. |
| Azarkish 2015 | Breast | NR | 1 yr | Single question on symptom | Cat (No sx) | Single question on employment | Cat (Employment/RTW) | OR | 3.14 | 95% CI: 1.51 to 4.76 | Not specified if univariate or multivariable regression conducted. Error in estimates reported (ie. beta estimates reported as ORs. Exclusion criteria of not working in a governmental job not justified. |
| Check 2019 | H&N (Oropharyngeal squamous cell carcinoma) | 1 to 22 yr (Median = 6 yr) | >1 yr | MD Anderson Symptom Inventory-Head and Neck (MDASI-HN) module | Cont | Single question on employment | Cat | Mean scores | 0.9 (emp) vs 1.2 (unemp) | SD = 1.9 (emp) vs 2.3 (unemp) | p = 0.4. MDASI: higher scores indicate higher level of sx burden. |
| Chen 2019 | H&N (Oral cavity cancer) | NR | >6 m (Mean = 33 m) | University of Washington Quality of Life Questionnaire (UW-QoL) item | Cont | Single question on employment | Cat | Mean scores | 90.1 (RTW) vs 85.13 (no RTW) | SD = 14.27 (RTW) vs 14.88 (no RTW) | p = 0.026. UWQOL: higher scores indicate lower levels of sx burden. |
| Cooper 2013 | Various (Urology: 32%; breast: 31%) | [Median time since start of sick leave = 4.7 m] | [Median time since start of tx = 3.9 m] | EORTC-QLQ-Core 30 subscale | Cont | Single question on employment at baseline, 6m and 12m | Time to event | HR | - | - | Time to RTW measured from cessaton of work. Sx covariate not included in final model based on stepwise forward selection if not stated. |
| Cox-Martin 2020 | Various (% NR) | NR | NR | Single question on symptom | Cat | Single question on employment | Cat (Employment/RTW) | OR | 0.78 (Male); 0.34 (Female) | 95% CI: 0.37 to 1.64 (Male)  0.22 to 0.54 (Female) |  |
| Duijts 2017 | Various (Breast 48%) | 2 to 4 yr | NR | EORTC-QLQ-Core 30 subscale | Cont | Single question on employment at 2, 3 and 4 years after dx | Cat (Sustained unemployment, positive change, negative change, sustained employment) | Mean scores | 40.5 (sust unemp) vs 22.7 (+ change) vs 32.7 (- change) vs 22.4 (sust emp) | - | p < 0.001. EORTC: higher scores indicate higher level of sx burden. |
| Duijts 2017 | Various (Breast 48%) | 2 to 4 yr | NR | EORTC-QLQ-Core 30 subscale | Cont | Single question on employment at 2, 3 and 4 years after dx | Cat (Sustained employment) | OR | - | - | Time lag GEE model used to correlate work continuation one year later with symptom burden. Not included in final model based on univariate analysis, no estimates reported. |
| Fantoni 2010 | Breast | >2 yr | NR | Single question on symptom (No, yes - a little, yes - a lot) | Cat | Single question on employment | Cat (Employment/RTW) | OR | - | - | Sx burden measured at end of tx. Not included in final model based on univariate analysis, no estimates reported. |
| Fantoni 2010 | Breast | >2 yr | NR | Single question on symptom (No, yes - a little, yes - a lot) | Cat | Single question on employment | Time to event | RR | Yes, a little: 0.96; Yes, a lot: 0.64 | 95% CI: Yes, a little: 0.69 to 1.34 Yes, a lot: 0.43 to 0.96 | Time to RTW measured from start of tx. Sx burden measured at end of tx. |
| Kenzik 2015 | Colorectal | >1 yr | NR | Medical Outcomes Study Short Form-12 (MOS SF-12) | Cat (Not at all/a little bit, moderately/quite a bit/extremely) | Single question on employment at baseline and follow-up | Cat (Unemployment/Non-RTW) | OR | 3.35 | 95% CI: 2.31 to 4.48 | Demographic statistics based on full cohort, which includes subjects not analysed for job change. |
| Kerns 2020 | Testicular | NR | >1 yr (Median = 3.8 yr) | SF-36 item | Cat (Grade 0, 1, 2/3) | Single question on employment | Cat (Unemployment/Non-RTW) | OR | Grade 1: 1.78; Grade 2/3: 3.75 | 95% CI: Grade 1: 0.33 to 3.15 Grade 2/3: 2.06 to 6.81 | SF-36: threshold not justified. |
| Kim 2014 | Lung | Median = 4.1 yr | NR | EORTC-QLQ-Core 30 subscale | Cat (>66) | Single question on employment | Cat (Employment/RTW) | OR | - | - | Not included in final model based on univariate analysis. Univariate estimates = 1.42 (0.71-2.83). EORTC: threshold based on previous studies with >66 indicating problematic status. Demographic statistics based on full cohort, which includes subjects not analysed for RTW. |
| Landeiro 2018 | Breast | 24 m | NR | Single question on symptom | Cat | Single question on employment | Cat (Employment/RTW) | OR | - | - | Not included in final model based on stepwise forward selection, no estimates reported. |
| Lee 2017 | Breast | Up to 36 m | NR | EORTC-QLQ-Core 30 subscale | Cont | Single question on employment at baseline, 12m, 24m, 36m post dx | Cat (Employment/RTW) | OR | - | - | Symptom burden assessed at 12 and 24 months. Not included in final model based on univariate analysis, no estimates reported. |
| Lindbohm 2014 | Breast | 1 to 8 yr | NR | Short Form-12 General Health Survey (SF-12) question | Cat (None/a little, moderate/a lot) | Single question on employment | Cat (Employed, early retirement, other non-employment) | OR | Retire: 9.08; Other: 1.96 | 95% CI: Retire: 4.17 to 19.74 Other: 1.03 to 3.71 |  |
| Pryce 2007 | Various (Breast 48%) | NR | NR | Single question on symptom | Cat | Single question on employment | Cat (Employment/RTW) | OR | - | - | Not included in final model based on univariate analysis and stepwise backward selection, no estimates reported. |
| Steiner 2008 | Various (Breast 20%; GI tract 13%) | Mean = 23 m | NR | Supportive Care Needs Survey (Bonevski 2000) item | Cat | Adapted from Health and Retirement Survey | Cat | Proportion | - | - | No estimates reported, assumed to be p > 0.05. |
| Tevaarwerk 2013 | Various (Breast 74%) | >6 m (6 to 12 m: 13%; 12 to 24 m: 24%; >24 m: 63%) | NR | MD Anderson Symptom Inventory-Modified (MDASI-ECOG) | Cat (<4, >=5) | Two questions on current employment status and if changes occurred due to illness | Cat (Unemployment/Non-RTW) | OR | - | - | Not included in final model based on stepwise forward selection, no estimates for final model reported. Univariate estimates = 7.2 (3.8-13.6). MDASI: threshold based on MDASI recommendations with >=5 indicating severe level. |
| Verdonck-de Leeuw 2010 | H&N | NR | >2 yr | EORTC-QLQ-Core 30 subscale | Cont | Single question on employment change | Cat (Return to same work, changed work, no RTW) | Mean scores | 13.69 (same) vs 20.83 (changed) vs 22.92 (no RTW) | SD = 22.24 (same) vs 2545 (changed) vs 23.47 (no RTW) | EORTC: higher scores indicate higher level of sx burden. |
| Yoo 2013 | Gynae (Cervical) | NR | <5 yr: 29%; >=5 yr: 71% | EORTC-QLQ-Core 30 subscale | Cont | Single question on employment | Cat | Mean scores | 21.59 (emp) vs 24.32 (unemp) | - | GLM used to control for demographic and clinical covariates. EORTC: higher scores indicate higher level of symptom burden. |
| Zomkowski 2020 | Breast | [Mean time from surgery = 48 m] | >1 m | EORTC-QLQ-Core 30 single item | Cont | Single question on employment | Cat | Mean scores | 34.67 (RTW) vs 48.57 (no RTW) | SD = 30.64 (RTW) vs 33.27 (no RTW) | p = 0.048. EORTC: higher scores indicate higher level of sx burden. |
| **Work status (Early retirement/disability)** | | | | | | | | | | | |
| Dahl 2020 | Gynae (Cervical) | Mean = 11 yr | NR | EORTC-QLQ-Core 30 single item | Cont | Single question on disability | Cat (Disability) | OR | 1.02 | 95% CI: 1.01 to 1.03 | Disability defined as being on disability pension. EORTC: higher scores indicate higher level of sx burden. |
| Kerns 2020 | Testicular | NR | >1 yr (Median = 3.8 yr) | SF-36 item | Cat (Grade 0, 1, 2/3) | Single question on disability | Cat (Disability) | OR | Grade 1: 1.28; Grade 2/3: 10.6 | 95% CI: Grade 1: 0.33 to 5.03 Grade 2/3: 4.4 to 25.4 | Disability defined as being on disability leave. SF-36: threshold not justified. |
| Mehnert 2017 | Various (Breast 59.5%) | NR | [1 yr from rehab] | Brief Pain Scale (BPI) | Cont | Single question on employment | Cat (Early retirement) | OR | 1.19 | 95% CI: 1.02 to 1.38 | BPI: higher scores indicate more pain. |
| Vartanian 2006 | H&N | NR | [>2 yr from disease remission] | University of Washington Quality of Life Questionnaire (UW-QoL) item | Cat (Yes, ie. <100) | Single question on retirement | Cat (Early retirement) | OR | - | - | Not included in final model based on univariate analysis, no estimates reported. UW-QoL: threshold not justified but represents presence of sx. |
| **Work ability** | | | | | | | | | | | |
| Musti 2018 | Breast | >2 yr | NR | Single question on therapy | Cat | Single question on work ability | Cat | OR | 1.24 | 95% CI: 0.6 to 2.56 |  |
| **Work productivity** | | | | | | | | | | | |
| Calvio 2010 | Breast | NR | 1 to 10 yr (Mean = 3 yr) | VAS of 10cm | Cont | Work Limitations Questionnaire (WLQ) subscale | Cont | Beta | - | - | Productivity defined as work output measured using WLQ. Not included in final model based on multistep univariate analysis, no estimates for final model reported. Univariate analysis with demographic confounders = 0.14. WLQ: higher scores indicate lower output. |
| **Others (Changes in work)** | | | | | | | | | | | |
| Steiner 2008 | Various (Breast 20%; GI tract 13%) | Mean = 23 m | NR | Supportive Care Needs Survey (Bonevski 2000) item | Cat | Adapted from Health and Retirement Survey | Cat | Proportion | 54% (With changes in work) vs 38% (None) | - | Change in work defined as changes in work characteristics or reduction in work hours. p = 0.12. |
| **Others (Occupational role limitations)** | | | | | | | | | | | |
| Steiner 2008 | Various (Breast 20%; GI tract 13%) | Mean = 23 m | NR | Supportive Care Needs Survey (Bonevski 2000) item | Cat | Occupational Role Questionnaire items | Cat | Proportion | 61% (With limitations) vs 30% (None) | - | p = 0.004. |

**Neurological: Seizures**

| Study | Cancer | Time since dx | Time since tx | Symptom | | Work outcome | | Effect measure | Estimate | Spread | Notes |
| --- | --- | --- | --- | --- | --- | --- | --- | --- | --- | --- | --- |
|  |  |  |  | Measurement tool | Variable | Measurement tool | Variable |  |  |  |  |
| **Work status (Employment/RTW)** | | | | | | | | | | | |
| Back 2019 | Brain (Anaplastic glioma) | NR | [3 yr after RT] | National Cancer Institute Common Toxicity Criteria (CTC) | Cat (Grade 1 and above) | Single question on employment | Cat (Unemployment/Non-RTW) | OR | 1.1 | - | Symptom burden assessed at year 1 post RT. p value and SE not specified but statistically not significant. |
| Ng 2020 | Brain (Glioma) | [Mean time from diagnosis to treatment = 34.4 m] | Up to 12 m | Clinical diagnosis | Cat | Single question on employment | Time to event | HR | - | - | Seizures were assessed postoperatively. Results state that seizures delayed RTW with p < 0.02 for log rank test but no HR reported. |

**Neurological: Sensory issues (Taste)**

| Study | Cancer | Time since dx | Time since tx | Symptom | | Work outcome | | Effect measure | Estimate | Spread | Notes |
| --- | --- | --- | --- | --- | --- | --- | --- | --- | --- | --- | --- |
|  |  |  |  | Measurement tool | Variable | Measurement tool | Variable |  |  |  |  |
| **Work status (Employment/RTW)** | | | | | | | | | | | |
| Agarwal 2017 | H&N | NR | >6 m (Mean = 19 m) | EORTC-QLQ-H&N 35 subscale | Cont | Single question on employment | Cat | Mean scores | 16.9 (RTW) vs 12.1 (non-RTW) | SD = 21 (RTW) vs 16.6 (non-RTW) | p = 0.172. EORTC: higher scores indicate higher level of sx burden. |
| Check 2019 | H&N (Oropharyngeal squamous cell carcinoma) | 1 to 22 yr (Median = 6 yr) | >1 yr | MD Anderson Symptom Inventory-Head and Neck (MDASI-HN) module | Cont | Single question on employment | Cat | Mean scores | 1.5 (emp) vs 2.4 (unemp) | SD = 2.2 (emp) vs 2.8 (unemp) | p < 0.001. MDASI: higher scores indicate higher level of sx burden. |
| Chen 2019 | H&N (Oral cavity cancer) | NR | >6 m (Mean = 33 m) | University of Washington Quality of Life Questionnaire (UW-QoL) item | Cont | Single question on employment | Cat | Mean scores | 85.21 (RTW) vs 68.72 (no RTW) | SD = 22.85 (RTW) vs 36.76 (no RTW) | p = 0.001. UWQOL: higher scores indicate lower levels of sx burden. |
| Verdonck-de Leeuw 2010 | H&N | NR | >2 yr | EORTC-QLQ-H&N 35 subscale | Cont | Single question on employment change | Cat (Return to same work, changed work, no RTW) | Mean scores | 12.5 (same) vs 13.54 (changed) vs 27.08 (no RTW) | SD = 17.35 (same) vs 20.38 (changed) vs 30.78 (no RTW) | EORTC: higher scores indicate higher level of sx burden. |

**Neurological: Sensory issues (Hearing)**

| Study | Cancer | Time since dx | Time since tx | Symptom | | Work outcome | | Effect measure | Estimate | Spread | Notes |
| --- | --- | --- | --- | --- | --- | --- | --- | --- | --- | --- | --- |
|  |  |  |  | Measurement tool | Variable | Measurement tool | Variable |  |  |  |  |
| **Work status (Employment/RTW)** | | | | | | | | | | | |
| Dieluweit 2011 | Various (Lymphoma 31%; malignant bone tumors 21%) | >5 yr (Mean = 13.7 yr) | NR | Study-specific questionnaire | Cat | Single question on employment | Cat (Employment/RTW) | OR | - | - | Not included in final model based on stepwise backward selection, no estimates reported. |
| Kerns 2020 | Testicular | NR | >1 yr (Median = 3.8 yr) | Hearing Handicap Inventory | Cat (Grade 0, 1, 2/3) | Single question on employment | Cat (Unemployment/Non-RTW) | OR | Grade 1: 0.70; Grade 2/3: 1.82 | 95% CI: Grade 1: 0.39 to 1.25 Grade 2/3: 1.04 to 3.17 | PROs: threshold not justified. |
| Kerns 2020 | Testicular | NR | >1 yr (Median = 3.8 yr) | Audiometry | Cat (Norma, mild, mod, mod severe, severe/profound) | Single question on employment | Cat (Unemployment/Non-RTW) | OR | Mild: 0.74; Mod: 0.80; Mod sev: 0.65; Sev: 0.77 | 95% CI: Mild:  0.38 to 1.44 Mod:  0.34 to 1.85 Mod sev: 0.27 to 1.55 Sev:  0.27 to 2.16 | PROs: threshold based on clinical grading. |
| **Work status (Early retirement/disability)** | | | | | | | | | | | |
| Kerns 2020 | Testicular | NR | >1 yr (Median = 3.8 yr) | Hearing Handicap Inventory | Cat (Grade 0, 1, 2/3) | Single question on disability | Cat (Disability) | OR | Grade 1: 0.68; Grade 2/3: 1.33 | 95% CI: Grade 1: 0.25 to 1.80 Grade 2/3: 0.52 to 3.45 | Disability defined as being on disability leave. PROs: threshold not justified. |
| Kerns 2020 | Testicular | NR | >1 yr (Median = 3.8 yr) | Audiometry | Cat (Norma, mild, mod, mod severe, severe/profound) | Single question on disability | Cat (Disability) | OR | Mild: 1.34; Mod: 1.75; Mod sev: 1.75; Sev: 1.05 | 95% CI: Mild:  0.22 to 8.09 Mod:  0.26 to 11.7  Mod sev:  0.16 to 6.82 Sev:  0.47 to 21.9 | Disability defined as being on disability leave. PROs: threshold based on clinical grading. |

**Neurological: Sensory issues (Smell)**

| Study | Cancer | Time since dx | Time since tx | Symptom | | Work outcome | | Effect measure | Estimate | Spread | Notes |
| --- | --- | --- | --- | --- | --- | --- | --- | --- | --- | --- | --- |
|  |  |  |  | Measurement tool | Variable | Measurement tool | Variable |  |  |  |  |
| **Work status (Employment/RTW)** | | | | | | | | | | | |
| Agarwal 2017 | H&N | NR | >6 m (Mean = 19 m) | EORTC-QLQ-H&N 35 subscale | Cont | Single question on employment | Cat | Mean scores | 16.9 (RTW) vs 12.1 (non-RTW) | SD = 21 (RTW) vs 16.6 (non-RTW) | p = 0.172. EORTC: higher scores indicate higher level of sx burden. |
| Verdonck-de Leeuw 2010 | H&N | NR | >2 yr | EORTC-QLQ-H&N 35 subscale | Cont | Single question on employment change | Cat (Return to same work, changed work, no RTW) | Mean scores | 12.5 (same) vs 13.54 (changed) vs 27.08 (no RTW) | SD = 17.35 (same) vs 20.38 (changed) vs 30.78 (no RTW) | EORTC: higher scores indicate higher level of sx burden. |

**Neurological: Sensory issues (Sight)**

| Study | Cancer | Time since dx | Time since tx | Symptom | | Work outcome | | Effect measure | Estimate | Spread | Notes |
| --- | --- | --- | --- | --- | --- | --- | --- | --- | --- | --- | --- |
|  |  |  |  | Measurement tool | Variable | Measurement tool | Variable |  |  |  |  |
| **Work status (Employment/RTW)** | | | | | | | | | | | |
| Dieluweit 2011 | Various (Lymphoma 31%; malignant bone tumors 21%) | >5 yr (Mean = 13.7 yr) | NR | Study-specific questionnaire | Cat | Single question on employment | Cat (Employment/RTW) | OR | - | - | Not included in final model based on stepwise backward selection, no estimates reported. |

**Gastrointestinal: Anorexia**

| Study | Cancer | Time since dx | Time since tx | Symptom | | Work outcome | | Effect measure | Estimate | Spread | Notes |
| --- | --- | --- | --- | --- | --- | --- | --- | --- | --- | --- | --- |
|  |  |  |  | Measurement tool | Variable | Measurement tool | Variable |  |  |  |  |
| **Work status (Employment/RTW)** | | | | | | | | | | | |
| Agarwal 2017 | H&N | NR | >6 m (Mean = 19 m) | EORTC-QLQ-Core 30 single item | Cont | Single question on employment | Cat | Mean scores | 14.4 (RTW) vs 16.3 (non-RTW) | SD = 24.8 (RTW) vs 16.8 (non-RTW) | p = 0.364. EORTC: higher scores indicate higher level of sx burden. |
| Check 2019 | H&N (Oropharyngeal squamous cell carcinoma) | 1 to 22 yr (Median = 6 yr) | >1 yr | MD Anderson Symptom Inventory-Head and Neck (MDASI-HN) module | Cont | Single question on employment | Cat | Mean scores | 0.7 (emp) vs 1.3 (unemp) | SD = 1.7 (emp) vs 2.4 (unemp) | p < 0.001. MDASI: higher scores indicate higher level of sx burden. |
| Cooper 2013 | Various (Urology: 32%; breast: 31%) | [Median time since start of sick leave = 4.7 m] | [Median time since start of tx = 3.9 m] | EORTC-QLQ-Core 30 single item | Cont | Single question on employment at baseline, 6m and 12m | Time to event | HR | - | - | Time to RTW measured from cessaton of work. Sx covariate not included in final model based on stepwise forward selection if not stated. |
| Duijts 2017 | Various (Breast 48%) | 2 to 4 yr | NR | EORTC-QLQ-Core 30 single item | Cont | Single question on employment at 2, 3 and 4 years after dx | Cat (Sustained unemp, +change, -change, sustained emp) | Mean scores | 12 (sust unemp) vs -5.5 (+ch) vs 14.6 (-ch) vs 7 (sust emp) | - | p = 0.003. EORTC: higher scores indicate higher level of sx burden. |
| Duijts 2017 | Various (Breast 48%) | 2 to 4 yr | NR | EORTC-QLQ-Core 30 single item | Cont | Single question on employment at 2, 3 and 4 years after dx | Cat (Sustained employment) | OR | - | - | Time lag GEE model used to correlate work continuation one year later with symptom burden. Not included in final model based on univariate analysis, no estimates reported. |
| Kim 2014 | Lung | Median = 4.1 yr | NR | EORTC-QLQ-Core 30 single item | Cat (>66) | Single question on employment | Cat (Employment/RTW) | OR | - | - | Not included in final model based on univariate analysis. Univariate estimates = 1.41 (0.82-2.43). EORTC: threshold based on previous studies with >66 indicating problematic status. Demographic statistics based on full cohort, which includes subjects not analysed for RTW. |
| Lee 2017 | Breast | Up to 36 m | NR | EORTC-QLQ-Core 30 single item | Cont | Single question on employment at baseline, 12m, 24m, 36m post dx | Cat (Employment/RTW) | OR | - | - | Symptom burden assessed at 12 and 24 months. Not included in final model based on univariate analysis, no estimates reported. |
| Tevaarwerk 2013 | Various (Breast 74%) | >6 m (6 to 12 m: 13%; 12 to 24 m: 24%; >24 m: 63%) | NR | MD Anderson Symptom Inventory-Modified (MDASI-ECOG) | Cat (<4, >=5) | Two questions on current employment status and if changes occurred due to illness | Cat (Unemployment/Non-RTW) | OR | - | - | Not included in final model based on univariate analysis and stepwise forward selection, no estimates reported as non-significant in univariate analysis. MDASI: threshold based on MDASI recommendations with >=5 indicating severe level. |
| Verdonck-de Leeuw 2010 | H&N | NR | >2 yr | EORTC-QLQ-Core 30 single item | Cont | Single question on employment change | Cat (Return to same work, changed work, no RTW) | Mean scores | 2.38 (same) vs 22.92 (changed) vs 16.67 (no RTW) | SD = 8.74 (same) vs 31.55 (changed) vs 25.19 (no RTW) | p < 0.05. EORTC: higher scores indicate higher level of sx burden. |
| Yoo 2013 | Gynae (Cervical) | NR | <5 yr: 29%; >=5 yr: 71% | EORTC-QLQ-Core 30 single item | Cont | Single question on employment | Cat | Mean scores | 23.57 (emp) vs 20.22 (unemp) | - | GLM used to control for demographic and clinical covariates. EORTC: higher scores indicate higher level of symptom burden. |

**Gastrointestinal: Weight gain**

| Study | Cancer | Time since dx | Time since tx | Symptom | | Work outcome | | Effect measure | Estimate | Spread | Notes |
| --- | --- | --- | --- | --- | --- | --- | --- | --- | --- | --- | --- |
|  |  |  |  | Measurement tool | Variable | Measurement tool | Variable |  |  |  |  |
| **Work status (Employment/RTW)** | | | | | | | | | | | |
| Agarwal 2017 | H&N | NR | >6 m (Mean = 19 m) | EORTC-QLQ-H&N 35 single item | Cont | Single question on employment | Cat | Mean scores | 40.9 (RTW) vs 29.8 (non-RTW) | SD = 49.3 (RTW) vs 46.2 (non-RTW) | p = 0.864. EORTC: higher scores indicate higher level of sx burden. |

**Gastrointestinal: Weight gain**

| Study | Cancer | Time since dx | Time since tx | Symptom | | Work outcome | | Effect measure | Estimate | Spread | Notes |
| --- | --- | --- | --- | --- | --- | --- | --- | --- | --- | --- | --- |
|  |  |  |  | Measurement tool | Variable | Measurement tool | Variable |  |  |  |  |
| **Work status (Employment/RTW)** | | | | | | | | | | | |
| Agarwal 2017 | H&N | NR | >6 m (Mean = 19 m) | EORTC-QLQ-H&N 35 single item | Cont | Single question on employment | Cat | Mean scores | 37.4 (RTW) vs 38.3 (non-RTW) | SD = 48.5 (RTW) vs 49.1 (non-RTW) | p = 0.466. EORTC: higher scores indicate higher level of sx burden. |

**Gastrointestinal: Nausea and vomiting**

| Study | Cancer | Time since dx | Time since tx | Symptom | | Work outcome | | Effect measure | Estimate | Spread | Notes |
| --- | --- | --- | --- | --- | --- | --- | --- | --- | --- | --- | --- |
|  |  |  |  | Measurement tool | Variable | Measurement tool | Variable |  |  |  |  |
| **Work status (Employment/RTW)** | | | | | | | | | | | |
| Agarwal 2017 | H&N | NR | >6 m (Mean = 19 m) | EORTC-QLQ-H&N 35 single item | Cont | Single question on employment | Cat | Mean scores | 37.4 (RTW) vs 38.3 (non-RTW) | SD = 48.5 (RTW) vs 49.1 (non-RTW) | p = 0.466. EORTC: higher scores indicate higher level of sx burden. |
| Agarwal 2017 | H&N | NR | >6 m (Mean = 19 m) | EORTC-QLQ-Core 30 subscale | Cont | Single question on employment | Cat | Mean scores | 7.1 (RTW) vs 3.2 (non-RTW) | SD = 17.7 (RTW) vs 8.2 (non-RTW) | p = 0.104. EORTC: higher scores indicate higher level of sx burden. |
| Check 2019 | H&N (Oropharyngeal squamous cell carcinoma) | 1 to 22 yr (Median = 6 yr) | >1 yr | MD Anderson Symptom Inventory-Head and Neck (MDASI-HN) module | Cont | Single question on employment | Cat | Mean scores | 0.1 (emp) vs 0.5 (unemp) | SD = 0.5 (emp) vs 1.6 (unemp) | p = 0.02. MDASI: higher scores indicate higher level of sx burden. |
| Cooper 2013 | Various (Urology: 32%; breast: 31%) | [Median time since start of sick leave = 4.7 m] | [Median time since start of tx = 3.9 m] | EORTC-QLQ-Core 30 subscale | Cont | Single question on employment at baseline, 6m and 12m | Time to event | HR | Uro: .98 | 95% CI: Uro: 0.97 to 1.00 | Time to RTW measured from cessation of work. Sx covariate not included in final model based on stepwise forward selection if not stated. EORTC: higher scores indicate higher level of sx burden. |
| Duijts 2017 | Various (Breast 48%) | 2 to 4 yr | NR | EORTC-QLQ-Core 30 subscale | Cont | Single question on employment at 2, 3 and 4 years after dx | Cat (Sustained unemp, +change, -change, sustained emp) | Mean scores | 11.6 (sust unemp) vs -0.2 (+ch) vs 7.9 (-ch) vs 4.5 (sust emp) | - | p = 0.022. EORTC: higher scores indicate higher level of sx burden. |
| Duijts 2017 | Various (Breast 48%) | 2 to 4 yr | NR | EORTC-QLQ-Core 30 subscale | Cont | Single question on employment at 2, 3 and 4 years after dx | Cat (Sustained employment) | OR | - | - | Time lag GEE model used to correlate work continuation one year later with symptom burden. Not included in final model based on univariate analysis, no estimates reported. |
| Kim 2014 | Lung | Median = 4.1 yr | NR | EORTC-QLQ-Core 30 subscale | Cat (>66) | Single question on employment | Cat (Employment/RTW) | OR | - | - | Not included in final model based on univariate analysis. Univariate estimates = 0.77 (0.25-2.37). EORTC: threshold based on previous studies with >66 indicating problematic status. Demographic statistics based on full cohort, which includes subjects not analysed for RTW. |
| Lee 2017 | Breast | Up to 36 m | NR | EORTC-QLQ-Core 30 subscale | Cont | Single question on employment at baseline, 12m, 24m, 36m post dx | Cat (Employment/RTW) | OR | - | - | Symptom burden assessed at 12 and 24 months. Not included in final model based on univariate analysis, no estimates reported. |
| Pryce 2007 | Various (Breast 48%) | NR | NR | Single question on symptom | Cat | Single question on employment | Cat (Employment/RTW) | OR | - | - | Not included in final model based on univariate analysis and stepwise backward selection, no estimates reported. |
| Steiner 2008 | Various (Breast 20%; GI tract 13%) | Mean = 23 m | NR | Supportive Care Needs Survey (Bonevski 2000) item | Cat | Adapted from Health and Retirement Survey | Cat | Proportion | - | - | No estimates reported, assumed to be p > 0.05. |
| Tevaarwerk 2013 | Various (Breast 74%) | >6 m (6 to 12 m: 13%; 12 to 24 m: 24%; >24 m: 63%) | NR | MD Anderson Symptom Inventory-Modified (MDASI-ECOG) | Cat (<4, >=5) | Two questions on current employment status and if changes occurred due to illness | Cat (Unemployment/Non-RTW) | OR | - | - | Not included in final model based on univariate analysis and stepwise forward selection, no estimates reported as non-significant in univariate analysis. MDASI: threshold based on MDASI recommendations ie. >=5 indicating severe level. |
| Verdonck-de Leeuw 2010 | H&N | NR | >2 yr | EORTC-QLQ-Core 30 subscale | Cont | Single question on employment change | Cat (Return to same work, changed work, no RTW) | Mean scores | 0.59 (same) vs 6.25 (changed) vs 6.25 (no RTW) | SD = 3.15 (same) vs 14.75 (changed) vs 12.4 (no RTW) | EORTC: higher scores indicate higher level of sx burden. |
| Yoo 2013 | Gynae (Cervical) | NR | <5 yr: 29%; >=5 yr: 71% | EORTC-QLQ-Core 30 subscale | Cont | Single question on employment | Cat | Mean scores | 14.41 (emp) vs 12.12 (unemp) | - | GLM used to control for demographic and clinical covariates. EORTC: higher scores indicate higher level of symptom burden. |
| **Others (Changes in work)** | | | | | | | | | | | |
| Steiner 2008 | Various (Breast 20%; GI tract 13%) | Mean = 23 m | NR | Supportive Care Needs Survey (Bonevski 2000) item | Cat | Adapted from Health and Retirement Survey | Cat | Proportion | 46% (With changes in work) vs 20% (None) | - | Change in work defined as changes in work characteristics or reduction in work hours. p = 0.009. |
| **Others (Occupational role limitations)** | | | | | | | | | | | |
| Steiner 2008 | Various (Breast 20%; GI tract 13%) | Mean = 23 m | NR | Supportive Care Needs Survey (Bonevski 2000) item | Cat | Occupational Role Questionnaire items | Cat | Proportion | 55% (With limitations) vs 23% (None) | - | p = 0.03. |

**Gastrointestinal: Diarrhea/Increased bowel urgency**

| Study | Cancer | Time since dx | Time since tx | Symptom | | Work outcome | | Effect measure | Estimate | Spread | Notes |
| --- | --- | --- | --- | --- | --- | --- | --- | --- | --- | --- | --- |
|  |  |  |  | Measurement tool | Variable | Measurement tool | Variable |  |  |  |  |
| **Work status (Employment/RTW)** | | | | | | | | | | | |
| Agarwal 2017 | H&N | NR | >6 m (Mean = 19 m) | EORTC-QLQ-Core 30 single item | Cont | Single question on employment | Cat | Mean scores | 4.1 (RTW) vs 2.8 (non-RTW) | SD = 14.8 (RTW) vs 11.7 (non-RTW) | p = 0.356. EORTC: higher scores indicate higher level of sx burden. |
| Bennett 2018 | Prostate | 18 to 42 m | NR | Expanded Prostate Cancer Index Composite (EPIC) item | Cat (No/very small/small, moderate/big) | Single question on employment change | Cat (Employment loss) | OR | 2.54 | 95% CI: 1.64-3.94 |  |
| Cooper 2013 | Various (Urology: 32%; breast: 31%) | [Median time since start of sick leave = 4.7 m] | [Median time since start of tx = 3.9 m] | EORTC-QLQ-Core 30 single item | Cont | Single question on employment at baseline, 6m and 12m | Time to event | HR | - | - | Time to RTW measured from cessaton of work. Sx covariate not included in final model based on stepwise forward selection if not stated. |
| Duijts 2017 | Various (Breast 48%) | 2 to 4 yr | NR | EORTC-QLQ-Core 30 single item | Cont | Single question on employment at 2, 3 and 4 years after dx | Cat (Sustained unemployment, positive change, negative change, sustained employment) | Mean scores | 10.3 (sust unemp) vs 15 (+ change) vs 14.4 (- change) vs 6.5 (sust emp) | - | p = 0.106. EORTC: higher scores indicate higher level of sx burden. |
| Duijts 2017 | Various (Breast 48%) | 2 to 4 yr | NR | EORTC-QLQ-Core 30 single item | Cont | Single question on employment at 2, 3 and 4 years after dx | Cat (Sustained employment) | OR | - | - | Time lag GEE model used to correlate work continuation one year later with symptom burden. Not included in final model based on univariate analysis, no estimates reported. |
| Kim 2014 | Lung | Median = 4.1 yr | NR | EORTC-QLQ-Core 30 single item | Cat (>66) | Single question on employment | Cat (Employment/RTW) | OR | - | - | Not included in final model based on univariate analysis. Univariate estimates = 1.03 (0.44-2.42). EORTC: threshold based on previous studies with >66 indicating problematic status. Demographic statistics based on full cohort, which includes subjects not analysed for RTW. |
| Lee 2017 | Breast | Up to 36 m | NR | EORTC-QLQ-Core 30 single item | Cont | Single question on employment at baseline, 12m, 24m, 36m post dx | Cat (Employment/RTW) | OR | - | - | Symptom burden assessed at 12 and 24 months. Not included in final model based on univariate analysis, no estimates reported. |
| Tevaarwerk 2013 | Various (Breast 74%) | >6 m (6 to 12 m: 13%; 12 to 24 m: 24%; >24 m: 63%) | NR | MD Anderson Symptom Inventory-Modified (MDASI-ECOG) | Cat (<4, >=5) | Two questions on current employment status and if changes occurred due to illness | Cat (Unemployment/Non-RTW) | OR | - | - | Not included in analysis as less than 1-% reported severe level. MDASI: threshold based on MDASI recommendations with >=5 indicating severe level. |
| Verdonck-de Leeuw 2010 | H&N | NR | >2 yr | EORTC-QLQ-Core 30 single item | Cont | Single question on employment change | Cat (Return to same work, changed work, no RTW) | Mean scores | 7.14 (same) vs 4.17 (changed) vs 12.5 (no RTW) | SD = 16.62 (same) vs 16.67 (changed) vs 17.25 (no RTW) | EORTC: higher scores indicate higher level of sx burden. |
| Yoo 2013 | Gynae (Cervical) | NR | <5 yr: 29%; >=5 yr: 71% | EORTC-QLQ-Core 30 single item | Cont | Single question on employment | Cat | Mean scores | 15.66 (emp) vs 15.53 (unemp) | - | GLM used to control for demographic and clinical covariates. EORTC: higher scores indicate higher level of symptom burden. |

**Gastrointestinal: Constipation**

| Study | Cancer | Time since dx | Time since tx | Symptom | | Work outcome | | Effect measure | Estimate | Spread | Notes |
| --- | --- | --- | --- | --- | --- | --- | --- | --- | --- | --- | --- |
|  |  |  |  | Measurement tool | Variable | Measurement tool | Variable |  |  |  |  |
| **Work status (Employment/RTW)** | | | | | | | | | | | |
| Agarwal 2017 | H&N | NR | >6 m (Mean = 19 m) | EORTC-QLQ-Core 30 single item | Cont | Single question on employment | Cat | Mean scores | 11 (RTW) vs 6.4 (non-RTW) | SD = 21.8 (RTW) vs 19.2 (non-RTW) | p = 0.058. EORTC: higher scores indicate higher level of sx burden. |
| Check 2019 | H&N (Oropharyngeal squamous cell carcinoma) | 1 to 22 yr (Median = 6 yr) | >1 yr | MD Anderson Symptom Inventory-Head and Neck (MDASI-HN) module | Cont | Single question on employment | Cat | Mean scores | 0.6 (emp) vs 1.3 (unemp) | SD = 1.4 (emp) vs 2.3 (unemp) | p < 0.001. MDASI: higher scores indicate higher level of sx burden. |
| Cooper 2013 | Various (Urology: 32%; breast: 31%) | [Median time since start of sick leave = 4.7 m] | [Median time since start of tx = 3.9 m] | EORTC-QLQ-Core 30 single item | Cont | Single question on employment at baseline, 6m and 12m | Time to event | HR | Uro: .98 | 95% CI: Uro: 0.97 to 1.00 | Time to RTW measured from cessation of work. Sx covariate not included in final model based on stepwise forward selection if not stated. EORTC: higher scores indicate higher level of sx burden. |
| Verdonck-de Leeuw 2010 | H&N | NR | >2 yr | EORTC-QLQ-Core 30 single item | Cont | Single question on employment change | Cat (Return to same work, changed work, no RTW) | Mean scores | 4.67 (same) vs 8.33 (changed) vs 12.5 (no RTW) | SD = 14.95 (same) vs 19.25 (changed) vs 24.8 (no RTW) | EORTC: higher scores indicate higher level of sx burden. |
| Duijts 2017 | Various (Breast 48%) | 2 to 4 yr | NR | EORTC-QLQ-Core 30 single item | Cont | Single question on employment at 2, 3 and 4 years after dx | Cat (Sustained unemp, +change, -change, sustained emp) | Mean scores | 18.5 (sust unemp) vs 7.5 (+ch) vs 15.4 (-ch) vs 8.3 (sust emp) | - | p = 0.016. EORTC: higher scores indicate higher level of sx burden. |
| Duijts 2017 | Various (Breast 48%) | 2 to 4 yr | NR | EORTC-QLQ-Core 30 single item | Cont | Single question on employment at 2, 3 and 4 years after dx | Cat (Sustained employment) | OR | - | - | Time lag GEE model used to correlate work continuation one year later with symptom burden. Not included in final model based on univariate analysis, no estimates reported. |
| Kim 2014 | Lung | Median = 4.1 yr | NR | EORTC-QLQ-Core 30 single item | Cat (>66) | Single question on employment | Cat (Employment/RTW) | OR | - | - | Not included in final model based on univariate analysis. Univariate estimates = 1.73 (0.85-3.51). EORTC: threshold based on previous studies with >66 indicating problematic status. Demographic statistics based on full cohort, which includes subjects not analysed for RTW. |
| Lee 2017 | Breast | Up to 36 m | NR | EORTC-QLQ-Core 30 single item | Cont | Single question on employment at baseline, 12m, 24m, 36m post dx | Cat (Employment/RTW) | OR | - | - | Symptom burden assessed at 12 and 24 months. Not included in final model based on univariate analysis, no estimates reported. |
| Tevaarwerk 2013 | Various (Breast 74%) | >6 m (6 to 12 m: 13%; 12 to 24 m: 24%; >24 m: 63%) | NR | MD Anderson Symptom Inventory-Modified (MDASI-ECOG) | Cat (<4, >=5) | Two questions on current employment status and if changes occurred due to illness | Cat (Unemployment/Non-RTW) | OR | - | - | Not included in final model based on univariate analysis and stepwise forward selection, no estimates reported as non-significant in univariate analysis. MDASI: threshold based on MDASI recommendations with >=5 indicating severe level. |
| Yoo 2013 | Gynae (Cervical) | NR | <5 yr: 29%; >=5 yr: 71% | EORTC-QLQ-Core 30 single item | Cont | Single question on employment | Cat | Mean scores | 34.82 (emp) vs 37.61 (unemp) | - | GLM used to control for demographic and clinical covariates. EORTC: higher scores indicate higher level of symptom burden. |

**Psychological: Anxiety**

| Study | Cancer | Time since dx | Time since tx | Symptom | | Work outcome | | Effect measure | Estimate | Spread | Notes |
| --- | --- | --- | --- | --- | --- | --- | --- | --- | --- | --- | --- |
|  |  |  |  | Measurement tool | Variable | Measurement tool | Variable |  |  |  |  |
| **Work status (Employment/RTW)** | | | | | | | | | | | |
| Chen 2019 | H&N (Oral cavity cancer) | NR | >6 m (Mean = 33 m) | University of Washington Quality of Life Questionnaire (UW-QoL) item | Cont | Single question on employment | Cat | Mean scores | 58.33 (RTW) vs 58.97 (no RTW) | SD = 26.22 (RTW) vs 28.54 (no RTW) | p = 0.878. UWQOL: higher scores indicate lower levels of sx burden. |
| Cooper 2013 | Various (Urology: 32%; breast: 31%) | [Median time since start of sick leave = 4.7 m] | [Median time since start of tx = 3.9 m] | Hospital Anxiety and Depression Scale (HADS) subscale | Cont | Single question on employment at baseline, 6m and 12m | Time to event | HR | - | - | Time to RTW measured from cessaton of work. Sx covariate not included in final model based on stepwise forward selection if not stated. |
| Dahl 2019 | Various (Breast 41%) | >6 yr (Mean = 17 yr) | NR | Hospital Anxiety and Depression Scale (HADS) subscale | Cont | Single question on employment | Cat (Unemployment/Non-RTW) | OR | - | - | Not included in final model due to multicollinearity. HADS: higher scores indicate higher level of sx burden. |
| Dumas 2020 | Breast | 2 yr (Median = 23 m) | NR | Hospital Anxiety and Depression Scale (HADS) subscale | Cat (Noncase, doubtful, case) | Single question on employment | Cat (Unemployment/Non-RTW) | OR | Doubtful: 1.71; Case: 1.47 | 95% CI: Doubtful: 1.26 to 2.32 Case: 1.02 to 2.11 | Symptom burden assessed approximately 1 year prior. HADS: threshold based on questionnaire developer. |
| Fantoni 2010 | Breast | >2 yr | NR | VAS of 0 to 10 | Cat (divided into tertiles) | Single question on employment | Cat (Employment/RTW) | OR | - | - | Sx burden measured at end of tx. Not included in final model based on univariate analysis, no estimates reported. |
| Fantoni 2010 | Breast | >2 yr | NR | VAS of 0 to 10 | Cat (divided into tertiles) | Single question on employment | Time to event | RR | - | - | Time to RTW measured from start of tx. Sx burden measured at end of tx. Not included in final model based on univariate analysis, no estimates reported. |
| Kiserud 2016 | Hemato (Lymphoma) | Mean = 12.4 yr | Mean = 9.7 yr | Hospital Anxiety and Depression Scale (HADS) subscale | Cont | Single question on employment | Cat (Unemployment/Non-RTW) | OR | - | - | Not included in final model based on clinical relevance/multicollinearity. Univariate estimates =2.23 (1.22-4.06). HADS: higher scores indicate higher level of sx burden. |
| Lindbohm 2014 | Breast | 1 to 8 yr | NR | Hospital Anxiety and Depression Scale (HADS) subscale | Cat (No, possible, probable) | Single question on employment | Cat (Employed, early retirement, other non-employment) | OR | Retire: Prob-0.68, Poss-0.58; Other: Prob-0.81, Poss-1.73 | 95% CI: Retire: Prob-0.34 to 1.35; Poss-0.19 to 1.74 Other: Prob-0.40 to 1.63; Poss-0.76 to 3.94 |  |
| Magyari 2017 | Hemato (Hodgkin lymphoma) | NR | NR | Hospital Anxiety and Depression Scale (HADS) subscale | Cont | Single question on employment | Cat | Mean scores | 4.87 (Emp) vs 6.69 (Unemp) | SD = 3.35 vs 3.64 | p = 0.004. Univariate analysis only. |
| Pryce 2007 | Various (Breast 48%) | NR | NR | Single question on symptom | Cat | Single question on employment | Cat (Employment/RTW) | OR | - | - | Not included in final model based on univariate analysis and stepwise backward selection, no estimates reported. |
| So 2020 | H&N (NPC) | NR | [>=4 yr disease free] Median = 7.3 yr | Hospital Anxiety and Depression Scale (HADS) subscale | Cont | Single question on employment | Cat | Mean scores | 5.7 (Emp) vs 7.4 (Unemp) | SD = 3.8 (Emp) vs 3.3 (Unemp) | HADS: higher scores indicate higher level of sx burden. |
| Steiner 2008 | Various (Breast 20%; GI tract 13%) | Mean = 23 m | NR | Supportive Care Needs Survey (Bonevski 2000) item | Cat | Adapted from Health and Retirement Survey | Cat | Proportion | - | - | No estimates reported, assumed to be p > 0.05. |
| Tamminga 2016 | Thyroid | Mean = 9.9 yr | NR | Hospital Anxiety and Depression Scale (HADS) subscale | Cont | Single question on employment | Cat (Unemployment/Non-RTW) | OR | 0.93 | 95% CI: 0.79 to 1.1 | HADS: higher score indicates higher level of sx burden. |
| Verdonck-de Leeuw 2010 | H&N | NR | >2 yr | Hospital Anxiety and Depression Scale (HADS) subscale | Cont | Single question on employment change | Cat (Return to same work, changed work, no RTW) | Mean scores | 4.67 (same) vs 5.87 (changed) vs 6.37 (no RTW) | SD = 3.16 (same) vs 4.06 (changed) vs 5.18 (no RTW) | HADS: higher scores indicate higher level of sx burden. |
| Yoo 2013 | Gynae (Cervical) | NR | <5 yr: 29%; >=5 yr: 71% | Hospital Anxiety and Depression Scale (HADS) subscale | Cont | Single question on employment | Cat | Mean scores | 7.14 (emp) vs 7.02 (unemp) | - | GLM used to control for demographic and clinical covariates. EORTC: higher scores indicate higher level of symptom burden. |
| **Work ability** | | | | | | | | | | | |
| Dahl 2019 | Various (Breast 41%) | >6 yr (Mean = 17 yr) | NR | Hospital Anxiety and Depression Scale (HADS) subscale | Cont | Work Ability Index (WAI) - 1st qns only | Cat (Low work ability, ie. <=7) | OR | - | - | Not included in final model due to multicollinearity. HADS: higher scores indicate higher level of sx burden; WAI: threshold of 7 previously validated. |
| Ho 2018 | Breast | >1 yr (Median = 4 yr) | NR | Hospital Anxiety and Depression Scale (HADS) subscale | Cont | Work Ability Index (WAI) | Cont | Std beta | - | - | Not included in final model based on stepwise backward selection, adjusted estimates from non-optimised full model = -0.2 (CI: -1.1 to 0.6). HADS: higher score indicates higher level of sx burden; WAI: higher scores indicate greater ability. |
| **Work productivity** | | | | | | | | | | | |
| Brick 2019 | Breast | NR | [6 m after surgery and start of chemotherapy or endocrine therapy] | Profile of Mood States (POMS) subscale | Cont | Work Limitations Questionnaire (WLQ) | Cont | Correlation | 0.331 | - | p = 0.270. No SE reported. POMS: higher scores indicated higher level of sx; WLQ: higher scores indicate lower productivity. |
| Calvio 2010 | Breast | NR | 1 to 10 yr (Mean = 3 yr) | Hospital Anxiety and Depression Scale (HADS) subscale | Cont | Work Limitations Questionnaire (WLQ) subscale | Cont | Beta | - | - | Productivity defined as work output measured using WLQ. Not included in final model based on multistep univariate analysis, no estimates for final model reported. HADS: higher scores indicate higher level of sx burden; WLQ: higher scores indicate lower output. |
| Cheng 2016 | Breast | NR | >6 m (Mean = 2.9 yr) | Hospital Anxiety and Depression Scale (HADS) subscale | Cont | Work Limitations Questionnaire (WLQ) | Cont | Beta | 0.18 | 95% CI:  -0.13 to 0.50 | HADS: higher scores indicate higher level of sx burden; WLQ: score converted to productivity loss. |
| Hansen 2008 | Breast | Mean = 3.8 yr | NR | Hospital Anxiety and Depression Scale (HADS) subscale | Cont | Work Limitations Questionnaire (WLQ) | Cont | Beta | 0.056 | 95% CI:  -0.096 to 0.208 | HADS: higher scores indicate higher level of sx burden; WLQ: higher scores indicate lower productivity. Patients with chronic comorbid diseases were excluded but no specification of which diseases. |
| Zeng 2017 | Breast | NR | >2 yr (Mean = 2.9 yr) | Hospital Anxiety and Depression Scale (HADS) subscale | Cont | Work Limitations Questionnaire (WLQ) | Cont | Beta | 0.005 | - | p = 0.014. No SE reported. HADS: higher scores indicate higher level of sx burden; WLQ: higher scores indicate lower productivity. |
| **Others (Changes in work)** | | | | | | | | | | | |
| Gudbergsson 2008 | Various (Breast 51%) | NR | 2 to 6 yr | Hospital Anxiety and Depression Scale (HADS) subscale | Cont | Five questions on various aspects of work | Cat (Work changes occured if "Yes" to any question) | Mean scores | 6.9 (With work changes) vs 4.6 (None) | SD = 4.0 (Work changes) vs 3.5 (None) | Not included in final model based on analysis plan. Change in work defined as changes related to employer, occupation, work tasks, unemployment, or pensioning. |
| Steiner 2008 | Various (Breast 20%; GI tract 13%) | Mean = 23 m | NR | Supportive Care Needs Survey (Bonevski 2000) item | Cat | Adapted from Health and Retirement Survey | Cat | Proportion | 81% (With changes in work) vs 63% (None) | - | Change in work defined as changes in work characteristics or reduction in work hours. p = 0.05. |
| **Others (Occupational role limitations)** | | | | | | | | | | | |
| Steiner 2008 | Various (Breast 20%; GI tract 13%) | Mean = 23 m | NR | Supportive Care Needs Survey (Bonevski 2000) item | Cat | Occupational Role Questionnaire items | Cat | Proportion | 80% (With limitations) vs 65% (None) | - | p = 0.1. |

**Psychological: Depression**

| Study | Cancer | Time since dx | Time since tx | Symptom | | Work outcome | | Effect measure | Estimate | Spread | Notes |
| --- | --- | --- | --- | --- | --- | --- | --- | --- | --- | --- | --- |
|  |  |  |  | Measurement tool | Variable | Measurement tool | Variable |  |  |  |  |
| **Work status (Employment/RTW)** | | | | | | | | | | | |
| Check 2019 | H&N (Oropharyngeal squamous cell carcinoma) | 1 to 22 yr (Median = 6 yr) | >1 yr | MD Anderson Symptom Inventory-Head and Neck (MDASI-HN) module | Cont | Single question on employment | Cat | Mean scores | 0.9 (emp) vs 1.3 (unemp) | SD = 1.8 (emp) vs 2.2 (unemp) | p = 0.005. MDASI: higher scores indicate higher level of sx burden. |
| Chen 2019 | H&N (Oral cavity cancer) | NR | >6 m (Mean = 33 m) | University of Washington Quality of Life Questionnaire (UW-QoL) item | Cont | Single question on employment | Cat | Mean scores | 61.04 (RTW) vs 52.31 (no RTW) | SD = 29.25 (RTW) vs 31.62 (no RTW) | p = 0.061. UWQOL: higher scores indicate lower levels of sx burden. |
| Cooper 2013 | Various (Urology: 32%; breast: 31%) | [Median time since start of sick leave = 4.7 m] | [Median time since start of tx = 3.9 m] | Hospital Anxiety and Depression Scale (HADS) subscale | Cont | Single question on employment at baseline, 6m and 12m | Time to event | HR | Gynae: 0.89 | 95% CI: Gynae: 0.81 to 1.00 | Time to RTW measured from cessation of work. Sx covariate not included in final model based on stepwise forward selection if not stated. EORTC: higher scores indicate higher level of sx burden. |
| Dahl 2019 | Various (Breast 41%) | >6 yr (Mean = 17 yr) | NR | Patient Health Questionnaire-9 (PHQ-9) | Cont | Single question on employment | Cat (Unemployment/Non-RTW) | OR | 1.06 | 95% CI: 1.01 to 1.11 | PHQ-9: higher scores indicate higher level of sx burden. |
| Duijts 2017 | Various (Breast 48%) | 2 to 4 yr | NR | Center for Epidemiologic Studies Depression Scale (CES-D) | Cont | Single question on employment at 2, 3 and 4 years after dx | Cat (Sustained unemployment, positive change, negative change, sustained employment) | Mean scores | 42.4 (sust unemp) vs 18.6 (+ change) vs 15.3 (- change) vs 13.6 (sust emp) | - | p < 0.001. CES-D: higher scores indicate more severe depression. |
| Duijts 2017 | Various (Breast 48%) | 2 to 4 yr | NR | Center for Epidemiologic Studies Depression Scale (CES-D) | Cont | Single question on employment at 2, 3 and 4 years after dx | Cat (Sustained employment) | OR | - | - | Time lag GEE model used to correlate work continuation one year later with symptom burden. Not included in final model based on univariate analysis, no estimates reported. |
| Dumas 2020 | Breast | 2 yr (Median = 23 m) | NR | Hospital Anxiety and Depression Scale (HADS) subscale | Cat (Noncase, doubtful, case) | Single question on employment | Cat (Unemployment/Non-RTW) | OR | Doubtful: 1.05; Case: 2.29 | 95% CI: Doubtful: 1.07 to 1.59 Case: 1.34 to 3.91 | Symptom burden assessed approximately 1 year prior. HADS: threshold based on questionnaire developer. |
| Ekenga 2019 | Breast | NR | [>2 yr after surgery] | Clinical diagnosis/ therapy | Cat | Single question on employment change | Cat (Diminished employment) | OR | - | - | Not included in final model based on univariate analysis, no estimates reported. |
| Kiserud 2016 | Hemato (Lymphoma) | Mean = 12.4 yr | Mean = 9.7 yr | Hospital Anxiety and Depression Scale (HADS) subscale | Cont | Single question on employment | Cat (Unemployment/Non-RTW) | OR | 0.9 | 95% CI: 0.76 to 1.06 | HADS: higher scores indicate higher level of sx burden. |
| Koch 2015 | H&N | >2 yr (Mean = 5.6 yr) | >2 yr | Patient Health Questionnaire-9 (PHQ-9) | Cont | Single question on employment | Cat | Mean scores | 5.7 (emp) vs 8.3 (unemp) | SD = 4.0 (emp) vs 4.7 (unemp) | p = 0.042. PHQ-9: higher scores indicate higher sx burden. |
| Landeiro 2018 | Breast | 24 m | NR | Single question on symptom | Cat | Single question on employment | Cat (Employment/RTW) | OR | 0.07 | 95% CI: 0.01 to 0.63 |  |
| Lindbohm 2014 | Breast | 1 to 8 yr | NR | Hospital Anxiety and Depression Scale (HADS) subscale | Cat (No, possible/probable) | Single question on employment | Cat (Employed, early retirement, other non-employment) | OR | Retire: 2.32; Other: 1.07 | 95% CI: Retire: 0.96 to 5.62 Other: 0.45 to 2.59 |  |
| Magyari 2017 | Hemato (Hodgkin lymphoma) | NR | NR | Hospital Anxiety and Depression Scale (HADS) subscale | Cont | Single question on employment | Cat | Mean scores | 2.17 (Emp) vs 4.87 (Unemp) | SD = 2.38 vs 3.84 | P < 0.001. Univariate analysis only. |
| Nieuwenhuijsen 2009 | Various (GI tract 31%; breast 20%) | [12 m after first day of sick leave] | NR | Center for Epidemiologic Studies Depression Scale (CES-D) | Cont | Single question on employment | Cat (Employment/RTW) | OR | - | - | No estimates reported but not statistically signficant. |
| Pryce 2007 | Various (Breast 48%) | NR | NR | Single question on symptom | Cat | Single question on employment | Cat (Employment/RTW) | OR | - | - | Not included in final model based on univariate analysis and stepwise backward selection, no estimates reported. |
| Schmidt 2019 | Breast | NR | [>1 yr from surgery] Median = 247 days | Center for Epidemiologic Studies Depression Scale (CES-D) | Cont (Per unit of 25 units) | Several questions on working status and hours | Cat (Reduced hours or discontinuation of work) | OR | 2.9 | 95% CI: 1.1 to 8 | Symptom burden assessed at week 13 post surgery. CES-D: higher scores indicate higher level of sx burden. |
| Schmidt 2019 | Breast | NR | [>1 yr from surgery] Median = 247 days | Center for Epidemiologic Studies Depression Scale (CES-D) | Cont (Per unit of 25 units) | Several questions on working status and hours | Cat (Reduced hours or discontinuation of work) | OR | 1.3 | 95% CI: 0.3 to 3.9 | Symptom burden assessed at month 15 post surgery. CES-D: higher scores indicate higher level of sx burden. |
| So 2020 | H&N (NPC) | NR | [>=4 yr disease free] Median = 7.3 yr | Hospital Anxiety and Depression Scale (HADS) subscale | Cont | Single question on employment | Cat | Mean scores | 4.5(Emp) vs 6.4 (Unemp) | SD = 3.7 (Emp) vs 4.4 (Unemp) | HADS: higher scores indicate higher level of sx burden. |
| Spelten 2003 | Various (Gynae 25%; breast 24%) | [18 m after sick leave] | NR | Center for Epidemiologic Studies Depression Scale (CES-D) | Cat (Quartiles) | Single question on employment | Time to event | HR | 0.81 | 95% CI: 0.66 to 0.99 | Time to RTW measured from first day of sick leave. Symptom burden measured at 6 months after sick leave. CES-D: higher scores indicate higher level of sx burden. Depression modelled as quartiles but only one estimate reported. |
| Steiner 2008 | Various (Breast 20%; GI tract 13%) | Mean = 23 m | NR | Supportive Care Needs Survey (Bonevski 2000) item | Cat | Adapted from Health and Retirement Survey | Cat | Proportion | - | - | No estimates reported, assumed to be p > 0.05. |
| Syrjala 2004 | Hemato | NR | Up to 5 yr from transplant | Beck Depression Inventory (BDI) | Cat (No depression, mild, moderate, severe) | Single question on employment | Time to event | HR | - | - | RTW is defined as full RTW and time to RTW measured from transplant date. Not included in final model based on stepwise forward selection, no estimates reported. BDI: thresholds based on developed recommendations guided by general population norms. Demographic statistics based on full cohort, which includes subjects not analysed for RTW. |
| Tamminga 2016 | Thyroid | Mean = 9.9 yr | NR | Hospital Anxiety and Depression Scale (HADS) subscale | Cont | Single question on employment | Cat (Unemployment/Non-RTW) | OR | 1.02 | 95% CI: 0.91 to 1.15 | HADS: higher score indicates higher level of sx burden. |
| Tevaarwerk 2013 | Various (Breast 74%) | >6 m (6 to 12 m: 13%; 12 to 24 m: 24%; >24 m: 63%) | NR | MD Anderson Symptom Inventory-Modified (MDASI-ECOG) | Cat (<4, >=5) | Two questions on current employment status and if changes occurred due to illness | Cat (Unemployment/Non-RTW) | OR | - | - | Not included in final model based on stepwise forward selection, no estimates for final model reported. Univariate estimates = 5.9 (3.1-11.1). MDASI: threshold based on MDASI recommendations with >=5 indicating severe level. |
| Verdonck-de Leeuw 2010 | H&N | NR | >2 yr | Hospital Anxiety and Depression Scale (HADS) subscale | Cont | Single question on employment change | Cat (Return to same work, changed work, no RTW) | Mean scores | 2.89 (same) vs 4.29 (changed) vs 5 (no RTW) | SD = 3.12 (same) vs 3.36 (changed) vs 3.7 (no RTW) | HADS: higher scores indicate higher level of sx burden. |
| Yoo 2013 | Gynae (Cervical) | NR | <5 yr: 29%; >=5 yr: 71% | Hospital Anxiety and Depression Scale (HADS) subscale | Cont | Single question on employment | Cat | Mean scores | 6.31 (emp) vs 7.01 (unemp) | - | GLM used to control for demographic and clinical covariates. EORTC: higher scores indicate higher level of symptom burden. |
| **Work status (Early retirement/Disability)** | | | | | | | | | | | |
| Dahl 2020 | Gynae (Cervical) | Mean = 11 yr | NR | Patient Health Questionnaire-9 (PHQ-9) | Cont | Single question on disability | Cat (Disability) | OR | 1.17 | 95% CI: 1.07 to 1.29 | Disability defined as being on disability pension. PHQ-9: higher scores indicate higher level of sx burden. |
| Mehnert 2017 | Various (Breast 59.5%) | NR | [1 yr from rehab] | Hospital Anxiety and Depression Scale (HADS) subscale | Cat (Low, moderate, severe) | Single question on employment | Cat (Early retirement) | OR | - | - | Not included in final model based on stepwise backward selection, no estimates reported. |
| Van Muijen 2014, 2017, 2019 | Various (Breast 40%) | >24 m | NR | Center for Epidemiologic Studies Depression Scale (CES-D) | Cat (>16) | Institution-specified criteria | Cat (Disability) | OR | - | - | Work disability defined as >80% wage loss. Not included in final model based on stepwise backward selection, no estimates reported. CES-D: threshold of 16 used to indicate likely clinical depression. |
| **Work status (Income)** | | | | | | | | | | | |
| Koch 2015 | H&N | >2 yr (Mean = 5.6 yr) | >2 yr | Patient Health Questionnaire-9 (PHQ-9) | Cont | Single question on income | Cat (<500 Euro, 500-900 Euro, >900 Euro) | Mean scores | 8.8 (<500) vs 7.3 (500-900) vs 7.3 (>900) | SD = 1.9 (<500) vs 4.7 (500-900) vs 5.2 (>900) | p = 0.486. PHQ-9: higher scores indicate higher sx burden. |
| **Work status (Working hours)** | | | | | | | | | | | |
| Koch 2015 | H&N | >2 yr (Mean = 5.6 yr) | >2 yr | Patient Health Questionnaire-9 (PHQ-9) | Cont | Single question on working hours | Cat (>35h/wk, 15-35hr/wk, <15hr/wk, 0hr/wk) | Mean scores | 5.7 (>35) vs 3.0 (15-35) vs 8.1 (<15) vs 8.3 (0) | SD = 4.2 (>35) vs 0 (15-35) vs 1.8 (<15) vs 5.1 (0) | p = 0.133. PHQ-9: higher scores indicate higher sx burden. |
| **Work ability** | | | | | | | | | | | |
| Dahl 2019 | Various (Breast 41%) | >6 yr (Mean = 17 yr) | NR | Patient Health Questionnaire-9 (PHQ-9) | Cont | Work Ability Index (WAI) - 1st qns only | Cat (Low work ability, ie. <=7) | OR | 1.1 | 95% CI: 1.04 to 1.15 | PHQ-9: higher scores indicate higher level of sx burden; WAI: threshold of 7 previously validated. |
| Dorland 2018 | Various (Breast 46%) | 6, 12 and 18 m | NR | Patient Health Questionnaire-9 (PHQ-9) | Cont | Work Role Functioning Questionnaire (WRFQ) | Cont | Beta | -1.13 | 95% CI:  -1.74 to -0.77 | GEE used to control for time since RTW. PHQ-9: higher scores indicate higher level of sx burden; WRFQ: higher scores indicate better ability. |
| Ho 2018 | Breast | >1 yr (Median = 4 yr) | NR | Hospital Anxiety and Depression Scale (HADS) subscale | Cont | Work Ability Index (WAI) | Cont | Std beta | -1.6 | 95% CI:  -2.3 to -0.9 | HADS: higher score indicates higher level of sx burden; WAI: higher scores indicate greater ability. |
| Nieuwenhuijsen 2009 | Various (GI tract 31%; breast 20%) | [12 m after first day of sick leave] | NR | Center for Epidemiologic Studies Depression Scale (CES-D) | Cont | Work Ability Index (WAI) - 1st qns only | Cont | Beta | - | - | No estimates reported but not statistically signficant. |
| Van Muijen 2014, 2017, 2019 | Various (Breast 46%) | [36 m after sick leave] | NR | Center for Epidemiologic Studies Depression Scale (CES-D) | Cont | Work Ability Index (WAI) - 1st qns only | Cont | Std beta | - | - | Not included in final model based on stepwise forward selection, no estimates reported. |
| Van Muijen 2014, 2017, 2019 | Various (Breast 47%) | >24 m | NR | Center for Epidemiologic Studies Depression Scale (CES-D) | Cont | Work Ability Index (WAI) - 1st qns only | Cont | Std beta | -0.38 | - | CES-D: higher scores indicate higher level of sx burden; WAI: higher scores indicate greater ability. |
| **Work productivity** | | | | | | | | | | | |
| Brick 2019 | Breast | NR | [6 m after surgery and start of chemotherapy or endocrine tx] | Beck Depression Inventory (BDI) | Cont | Work Limitations Questionnaire (WLQ) | Cont | Correlation | 0.335 | - | p = 0.118. No SE reported. BDI: higher scores indicated higher level of sx; WLQ: higher scores indicate lower productivity. |
| Calvio 2010 | Breast | NR | 1 to 10 yr (Mean = 3 yr) | Hospital Anxiety and Depression Scale (HADS) subscale | Cont | Work Limitations Questionnaire (WLQ) subscale | Cont | Beta | - | - | Productivity defined as work output measured using WLQ. Not included in final model based on multistep univariate analysis, no estimates for final model reported. Univariate analysis with demographic confounders = 0.14. HADS: higher scores indicate higher level of sx burden; WLQ: higher scores indicate lower output. |
| Cheng 2016 | Breast | NR | >6 m (Mean = 2.9 yr) | Hospital Anxiety and Depression Scale (HADS) subscale | Cont | Work Limitations Questionnaire (WLQ) | Cont | Beta | 0.19 | 95% CI:  -0.09 to 0.48 | HADS: higher scores indicate higher level of sx burden; WLQ: score converted to productivity loss. |
| Hansen 2008 | Breast | Mean = 3.8 yr | NR | Hospital Anxiety and Depression Scale (HADS) subscale | Cont | Work Limitations Questionnaire (WLQ) | Cont | Beta | 0.045 | 95% CI:  -0.158 to 0.247 | HADS: higher scores indicate higher level of sx burden; WLQ: higher scores indicate lower productivity. Patients with chronic comorbid diseases were excluded but no specification of which diseases. |
| Zeng 2017 | Breast | NR | >2 yr (Mean = 2.9 yr) | Hospital Anxiety and Depression Scale (HADS) subscale | Cont | Work Limitations Questionnaire (WLQ) | Cont | Beta | 0.001 | - | p = 0.634. No SE reported. HADS: higher scores indicate higher level of sx burden; WLQ: higher scores indicate lower productivity. |
| **Others (Changes in work)** | | | | | | | | | | | |
| Gudbergsson 2008 | Various (Breast 51%) | NR | 2 to 6 yr | Hospital Anxiety and Depression Scale (HADS) subscale | Cont | Five questions on various aspects of work | Cat (Work changes occured if "Yes" to any question) | Mean scores | 4.3 (With work changes) vs 2.3 (None) | SD = 3.6 (Work changes) vs 2.5 (None) | Not included in final model based on analysis plan. Change in work defined as changes related to employer, occupation, work tasks, unemployment, or pensioning. |
| Steiner 2008 | Various (Breast 20%; GI tract 13%) | Mean = 23 m | NR | Supportive Care Needs Survey (Bonevski 2000) item | Cat | Adapted from Health and Retirement Survey | Cat | Proportion | 85% (With changes in work) vs 62% (None) | - | Change in work defined as changes in work characteristics or reduction in work hours. p = 0.01. |
| **Others (Occupational role limitations)** | | | | | | | | | | | |
| Steiner 2008 | Various (Breast 20%; GI tract 13%) | Mean = 23 m | NR | Supportive Care Needs Survey (Bonevski 2000) item | Cat | Occupational Role Questionnaire items | Cat | Proportion | 84% (With limitations) vs 63% (None) | - | p = 0.02. |

**Psychological: Body image issues (General)**

| Study | Cancer | Time since dx | Time since tx | Symptom | | Work outcome | | Effect measure | Estimate | Spread | Notes |
| --- | --- | --- | --- | --- | --- | --- | --- | --- | --- | --- | --- |
|  |  |  |  | Measurement tool | Variable | Measurement tool | Variable |  |  |  |  |
| **Work status (Employment/RTW)** | | | | | | | | | | | |
| Chen 2019 | H&N (Oral cavity cancer) | NR | >6 m (Mean = 33 m) | University of Washington Quality of Life Questionnaire (UW-QoL) item | Cont | Single question on employment | Cat | Mean scores | 67.71 (RTW) vs 55.13 (no RTW) | SD = 20.49 (RTW) vs 25.6 (no RTW) | p = 0.001. UWQOL: higher scores indicate lower levels of sx burden. |
| Lee 2017 | Breast | Up to 36 m | NR | EORTC-QLQ-Breast 23 subscale | Cont | Single question on employment at baseline, 12m, 24m, 36m post dx | Cat (Employment/RTW) | OR | 1.04 | 95% CI: 1.01 to 1.07 | Association significant for symptom burden assessed at 12 months; symptom burden assessed at 24 months not included in final model based on univariate analysis, no estimates reported. |
| Pryce 2007 | Various (Breast 48%) | NR | NR | Single question on symptom | Cat | Single question on employment | Cat (Employment/RTW) | OR | - | - | Not included in final model based on univariate analysis and stepwise backward selection, no estimates reported. |
| Zomkowski 2020 | Breast | [Mean time from surgery = 48 m] | >1 m | EORTC-QLQ-Breast 23 subscale | Cont | Single question on employment | Cat | Mean scores | 83.95 (RTW) vs 70.48 (no RTW) | SD = 26.75 (RTW) vs 31.14 (no RTW) | p = 0.039. EORTC: higher scores indicate lower level of sx burden. |

**Psychological: Body image issues (Alopecia)**

| Study | Cancer | Time since dx | Time since tx | Symptom | | Work outcome | | Effect measure | Estimate | Spread | Notes |
| --- | --- | --- | --- | --- | --- | --- | --- | --- | --- | --- | --- |
|  |  |  |  | Measurement tool | Variable | Measurement tool | Variable |  |  |  |  |
| **Work status (Employment/RTW)** | | | | | | | | | | | |
| Chen 2012 | Hemato (Hodgkin lymphoma) | >5 yr | NR | Adapted from the Childhood Cancer Survivor Study (CCSS) questionnaire | Cat | Single question on employment | Cat (Employment/RTW) | OR | - | - | Not included in final model based on stepwise backward selection, no estimates reported. |
| Tevaarwerk 2013 | Various (Breast 74%) | >6 m (6 to 12 m: 13%; 12 to 24 m: 24%; >24 m: 63%) | NR | MD Anderson Symptom Inventory-Modified (MDASI-ECOG) | Cat (<4, >=5) | Two questions on current employment status and if changes occurred due to illness | Cat (Unemployment/Non-RTW) | OR | - | - | Not included in final model based on univariate analysis and stepwise forward selection, no estimates reported as non-significant in univariate analysis. MDASI: threshold based on MDASI recommendations with >=5 indicating severe level. |

**Psychological: Body image issues (Scarring)**

| Study | Cancer | Time since dx | Time since tx | Symptom | | Work outcome | | Effect measure | Estimate | Spread | Notes |
| --- | --- | --- | --- | --- | --- | --- | --- | --- | --- | --- | --- |
|  |  |  |  | Measurement tool | Variable | Measurement tool | Variable |  |  |  |  |
| **Work status (Employment/RTW)** | | | | | | | | | | | |
| Chen 2012 | Hemato (Hodgkin lymphoma) | >5 yr | NR | Adapted from the Childhood Cancer Survivor Study (CCSS) questionnaire | Cat | Single question on employment | Cat (Denied job) | OR | 3 | 95% CI: 1.3 to 7 |  |

**Respiratory: Coughing**

| Study | Cancer | Time since dx | Time since tx | Symptom | | Work outcome | | Effect measure | Estimate | Spread | Notes |
| --- | --- | --- | --- | --- | --- | --- | --- | --- | --- | --- | --- |
|  |  |  |  | Measurement tool | Variable | Measurement tool | Variable |  |  |  |  |
| **Work status (Employment/RTW)** | | | | | | | | | | | |
| Agarwal 2017 | H&N | NR | >6 m (Mean = 19 m) | EORTC-QLQ-H&N 35 single item | Cont | Single question on employment | Cat | Mean scores | 12.2 (RTW) vs 20.6 (non-RTW) | SD = 20 (RTW) vs 23.6 (non-RTW) | p = 0.001. EORTC: higher scores indicate higher level of sx burden. |
| Check 2019 | H&N (Oropharyngeal squamous cell carcinoma) | 1 to 22 yr (Median = 6 yr) | >1 yr | MD Anderson Symptom Inventory-Head and Neck (MDASI-HN) module | Cont | Single question on employment | Cat | Mean scores | 1.9 (emp) vs 2.5 (unemp) | SD = 2.5 (emp) vs 2.8 (unemp) | p = 0.005. MDASI: higher scores indicate higher level of sx burden. |
| Cheville 2011 | Lung | 1 to 5 yr | NR | Lung Cancer Symptom Scale (LCSS) single item | Cat (<5) | Baecke Physical Activity Questionnaire single item | Cat (Employment/RTW) | OR | 0.97 | 95% CI: 0.62 to 1.5 | Mixed model analysis to control for time from dx. LCSS: threshold used for sx empirically based on previous work - <5 indicates presence of sx. |
| Verdonck-de Leeuw 2010 | H&N | NR | >2 yr | EORTC-QLQ-H&N 35 single item | Cont | Single question on employment change | Cat (Return to same work, changed work, no RTW) | Mean scores | 20.23 (same) vs 20.83 (changed) vs 25 (no RTW) | SD = 27.72 (same) vs 31.91 (changed) vs 34.5 (no RTW) | EORTC: higher scores indicate higher level of sx burden. |
| Tevaarwerk 2013 | Various (Breast 74%) | >6 m (6 to 12 m: 13%; 12 to 24 m: 24%; >24 m: 63%) | NR | MD Anderson Symptom Inventory-Modified (MDASI-ECOG) | Cat (<4, >=5) | Two questions on current employment status and if changes occurred due to illness | Cat (Unemployment/Non-RTW) | OR | - | - | Not included in final model based on stepwise forward selection, no estimates for final model reported. Univariate estimates = 6.5 (2.7-16.0). MDASI: threshold based on MDASI recommendations with >=5 indicating severe level. |

**Respiratory: Dyspnea**

| Study | Cancer | Time since dx | Time since tx | Symptom | | Work outcome | | Effect measure | Estimate | Spread | Notes |
| --- | --- | --- | --- | --- | --- | --- | --- | --- | --- | --- | --- |
|  |  |  |  | Measurement tool | Variable | Measurement tool | Variable |  |  |  |  |
| **Work status (Employment/RTW)** | | | | | | | | | | | |
| Agarwal 2017 | H&N | NR | >6 m (Mean = 19 m) | EORTC-QLQ-Core 30 single item | Cont | Single question on employment | Cat | Mean scores | 7.2 (RTW) vs 11.3 (non-RTW) | SD = 15.9 (RTW) vs 18.7 (non-RTW) | p = 0.864. EORTC: higher scores indicate higher level of sx burden. |
| Check 2019 | H&N (Oropharyngeal squamous cell carcinoma) | 1 to 22 yr (Median = 6 yr) | >1 yr | MD Anderson Symptom Inventory-Head and Neck (MDASI-HN) module | Cont | Single question on employment | Cat | Mean scores | 0.7 (emp) vs 1.1 (unemp) | SD = 1.5 (emp) vs 2.2 (unemp) | p = 0.12. MDASI: higher scores indicate higher level of sx burden. |
| Cheville 2011 | Lung | 1 to 5 yr | NR | Lung Cancer Symptom Scale (LCSS) single item | Cat (<5) | Baecke Physical Activity Questionnaire single item | Cat (Employment/RTW) | OR | 0.58 | 95% CI: 0.41 to 0.84 | Mixed model analysis to control for time from dx. LCSS: threshold used for sx empirically based on previous work - <5 indicates presence of sx. |
| Cooper 2013 | Various (Urology: 32%; breast: 31%) | [Median time since start of sick leave = 4.7 m] | [Median time since start of tx = 3.9 m] | EORTC-QLQ-Core 30 single item | Cont | Single question on employment at baseline, 6m and 12m | Time to event | HR | - | - | Time to RTW measured from cessaton of work. Sx covariate not included in final model based on stepwise forward selection if not stated. |
| Duijts 2017 | Various (Breast 48%) | 2 to 4 yr | NR | EORTC-QLQ-Core 30 single item | Cont | Single question on employment at 2, 3 and 4 years after dx | Cat (Sustained unemployment, positive change, negative change, sustained employment) | Mean scores | 23 (sust unemp) vs 23.6 (+ change) vs 27.3 (- change) vs 18 (sust emp) | - | p = 0.153. EORTC: higher scores indicate higher level of sx burden. |
| Duijts 2017 | Various (Breast 48%) | 2 to 4 yr | NR | EORTC-QLQ-Core 30 single item | Cont | Single question on employment at 2, 3 and 4 years after dx | Cat (Sustained employment) | OR | - | - | Time lag GEE model used to correlate work continuation one year later with symptom burden. Not included in final model based on univariate analysis, no estimates reported. |
| Kim 2014 | Lung | Median = 4.1 yr | NR | EORTC-QLQ-Core 30 single item | Cat (>66) | Single question on employment | Cat (Employment/RTW) | OR | - | - | Not included in final model based on stepwise selection. Univariate estimates = 1.75 (1.20-2.54). EORTC: threshold based on previous studies with >66 indicating problematic status. Demographic statistics based on full cohort, which includes subjects not analysed for RTW. |
| Lee 2017 | Breast | Up to 36 m | NR | EORTC-QLQ-Core 30 single item | Cont | Single question on employment at baseline, 12m, 24m, 36m post dx | Cat (Employment/RTW) | OR | - | - | Symptom burden assessed at 12 and 24 months. Not included in final model based on univariate analysis, no estimates reported. |
| Tevaarwerk 2013 | Various (Breast 74%) | >6 m (6 to 12 m: 13%; 12 to 24 m: 24%; >24 m: 63%) | NR | MD Anderson Symptom Inventory-Modified (MDASI-ECOG) | Cat (<4, >=5) | Two questions on current employment status and if changes occurred due to illness | Cat (Unemployment/Non-RTW) | OR | - | - | Not included in final model based on stepwise forward selection, no estimates for final model reported. Univariate estimates = 9.2 (4.1-20.3). MDASI: threshold based on MDASI recommendations with >=5 indicating severe level. |
| Verdonck-de Leeuw 2010 | H&N | NR | >2 yr | EORTC-QLQ-Core 30 single item | Cont | Single question on employment change | Cat (Return to same work, changed work, no RTW) | Mean scores | 9.52 (same) vs 14.58 (changed) vs 4.17 (no RTW) | SD = 21.96 (same) vs 29.74 (changed) vs 11.78 (no RTW) | EORTC: higher scores indicate higher level of sx burden. |
| Yoo 2013 | Gynae (Cervical) | NR | <5 yr: 29%; >=5 yr: 71% | EORTC-QLQ-Core 30 single item | Cont | Single question on employment | Cat | Mean scores | 24.12 (emp) vs 24.23 (unemp) | - | GLM used to control for demographic and clinical covariates. EORTC: higher scores indicate higher level of symptom burden. |

**Fatigue/sleep-related: Fatigue**

| Study | Cancer | Time since dx | Time since tx | Symptom | | Work outcome | | Effect measure | Estimate | Spread | Notes |
| --- | --- | --- | --- | --- | --- | --- | --- | --- | --- | --- | --- |
|  |  |  |  | Measurement tool | Variable | Measurement tool | Variable |  |  |  |  |
| **Work status (Employment/RTW)** | | | | | | | | | | | |
| Agarwal 2017 | H&N | NR | >6 m (Mean = 19 m) | EORTC-QLQ-Core 30 subscale | Cont | Single question on employment | Cat | Mean scores | 21.8 (RTW) vs 22.9 (non-RTW) | SD = 22.9 (RTW) vs 22.8 (non-RTW) | p = 0.669. EORTC: higher scores indicate higher level of sx burden. |
| Alleaume 2018 | Various (Breast 58%) | 5 yr | NR | EORTC-QLQ-Core 30 single item | Cat (Severe fatigue ie. >= 40) | Several questions on working status and hours | Cat (Employed without working time reduction, unemployed, employed with working time reduction) | OR | - | - | Not included in final model based on stepwise selection, no estimates reported. EORTC: threshold used not justified. |
| Balak 2008 | Breast | [2 yr since sick leave] | NR | Clinical diagnosis | Cat | Feedback to occupational health department | Time to event | HR | 1.67 | 95% CI: 0.76 to 3.64 | RTW is defined as partial RTW and time to RTW measured from first day of sick leave. Sx burden measured at time of RTW. Unexplained exclusion criteria of pre-existent chronic diseases that may lead to unemployment can potentially lead to selection bias. Differences in included and excluded pts may cause MNAR. |
| Balak 2008 | Breast | [2 yr since sick leave] | NR | Clinical diagnosis | Cat | Feedback to occupational health department | Time to event | HR | 0.79 | 95% CI: 0.37 to 1.7 | RTW is defined as full RTW and time to RTW measured from first day of sick leave. Sx burden measured at time of RTW. Unexplained exclusion criteria of pre-existent chronic diseases that may lead to unemployment can potentially lead to selection bias. Differences in included and excluded pts may cause MNAR. |
| Behringer 2016 | Hemato (Hodgkin lymphoma) | NR | 1 yr | EORTC-QLQ-Core 30 single item | Cat (Severe fatigue ie. >= 50) | Single question on employment | Cat (Employment/RTW) | OR | 0.33 | 95% CI: 0.26 to 0.41 | EORTC: threshold based on previous studies. |
| Behringer 2016 | Hemato (Hodgkin lymphoma) | NR | 2 yr | EORTC-QLQ-Core 30 single item | Cat (Severe fatigue ie. >= 50) | Single question on employment | Cat (Employment/RTW) | OR | 0.32 | 95% CI: 0.25 to 0.42 | EORTC: threshold based on previous studies. |
| Behringer 2016 | Hemato (Hodgkin lymphoma) | NR | 5 yr | EORTC-QLQ-Core 30 single item | Cat (Severe fatigue ie. >= 50) | Single question on employment | Cat (Employment/RTW) | OR | 0.29 | 95% CI: 0.21 to 0.41 | EORTC: threshold based on previous studies. |
| Check 2019 | H&N (Oropharyngeal squamous cell carcinoma) | 1 to 22 yr (Median = 6 yr) | >1 yr | MD Anderson Symptom Inventory-Head and Neck (MDASI-HN) module | Cont | Single question on employment | Cat | Mean scores | 1.8 (emp) vs 2.5 (unemp) | SD = 2.2 (emp) vs 2.8 (unemp) | p = 0.06. MDASI: higher scores indicate higher level of sx burden. |
| Cheville 2011 | Lung | 1 to 5 yr | NR | Lung Cancer Symptom Scale (LCSS) single item | Cat (<5) | Baecke Physical Activity Questionnaire single item | Cat (Employment/RTW) | OR | 0.7 | 95% CI: 0.49 to 1 | Mixed model analysis to control for time from dx. LCSS: threshold used for sx empirically based on previous work - <5 indicates presence of sx. |
| Cooper 2013 | Various (Urology: 32%; breast: 31%) | [Median time since start of sick leave = 4.7 m] | [Median time since start of tx = 3.9 m] | EORTC-QLQ-Core 30 subscale | Cont | Single question on employment at baseline, 6m and 12m | Time to event | HR | Breast: 0.99 | 95% CI: Breast: 0.98 to 1.00 | Time to RTW measured from cessation of work. Sx covariate not included in final model based on stepwise forward selection if not stated. EORTC: higher scores indicate higher level of sx burden. |
| Dahl 2015, 2016 | Prostate | NR | >3 yr | Fatigue Questionnaire (FQ) | Cat (Chronic fatigue, ie. TF>=4 for 6 months) | Single question on employment | Cat (Diminished employment) | OR | - | - | Not included in final model based on univariate analysis, no estimates for final model reported. Univariate estimates = 1.80 (0.89-3.75). |
| Dahl 2019 | Various (Breast 41%) | >6 yr (Mean = 17 yr) | NR | Fatigue Questionnaire (FQ) | Cont | Single question on employment | Cat (Unemployment/Non-RTW) | OR | 1.04 | 95% CI: 0.99 to 1.08 | FQ: higher scores indicate more fatigue. |
| Duijts 2017 | Various (Breast 48%) | 2 to 4 yr | NR | EORTC-QLQ-Core 30 subscale | Cont | Single question on employment at 2, 3 and 4 years after dx | Cat (Sustained unemployment, positive change, negative change, sustained employment) | Mean scores | 48.4 (sust unemp) vs 43.4 (+ change) vs 46.7 (- change) vs 38 (sust emp) | - | p = 0.007. EORTC: higher scores indicate higher level of sx burden. |
| Duijts 2017 | Various (Breast 48%) | 2 to 4 yr | NR | Functional Assessment of Chronic Illness-Fatigue scale (FACIT-F) | Cont | Single question on employment at 2, 3 and 4 years after dx | Cat (Sustained unemployment, positive change, negative change, sustained employment) | Mean scores | 20.6 (sust unemp) vs 23.6 (+ change) vs 27.6 (- change) vs 31.4 (sust emp) | - | p < 0.001. FACIT-F: higher scores indicate less fatigue. |
| Duijts 2017 | Various (Breast 48%) | 2 to 4 yr | NR | EORTC-QLQ-Core 30 subscale | Cont | Single question on employment at 2, 3 and 4 years after dx | Cat (Sustained employment) | OR | - | - | Time lag GEE model used to correlate work continuation one year later with symptom burden. Not included in final model based on univariate analysis, no estimates reported. |
| Duijts 2017 | Various (Breast 48%) | 2 to 4 yr | NR | Functional Assessment of Chronic Illness-Fatigue scale (FACIT-F) | Cont | Single question on employment at 2, 3 and 4 years after dx | Cat (Sustained employment) | OR | - | - | Time lag GEE model used to correlate work continuation one year later with symptom burden. Not included in final model based on univariate analysis, no estimates reported. |
| Dumas 2020 | Breast | 2 yr (Median = 23 m) | NR | EORTC-QLQ-Fatigue 12 subscale | Cat (Severe, ie. >=40) | Single question on employment | Cat (Unemployment/Non-RTW) | OR | 1.31 | 95% CI: 0.94 to 1.83 | Symptom burden assessed approximately 1 year prior. EORTC: threshold based on previous paper. |
| Ekenga 2019 | Breast | NR | [>2 yr after surgery] | RAND-36 vitality subscale | Cat (Below median) | Single question on employment change | Cat (Diminished employment) | OR | 5.71 | 95% CI: 2.48 to 13.15 | RAND-36: lower scores indicate more fatigue. Threshold not explained. |
| Fantoni 2010 | Breast | >2 yr | NR | VAS of 0 to 10 | Cat (divided into tertiles) | Single question on employment | Cat (Employment/RTW) | OR | - | - | Sx burden measured at end of tx. Not included in final model based on univariate analysis, no estimates reported. |
| Fantoni 2010 | Breast | >2 yr | NR | VAS of 0 to 10 | Cat (divided into tertiles) | Single question on employment | Time to event | RR | - | - | Time to RTW measured from start of tx. Sx burden measured at end of tx. Not included in final model based on univariate analysis, no estimates reported. |
| Kim 2014 | Lung | Median = 4.1 yr | NR | EORTC-QLQ-Core 30 subscale | Cat (>66) | Single question on employment | Cat (Employment/RTW) | OR | - | - | Not included in final model based on stepwise selection. Univariate estimates = 1.93 (1.08-3.45). EORTC: threshold based on previous studies with >66 indicating problematic status. Demographic statistics based on full cohort, which includes subjects not analysed for RTW. |
| Kiserud 2015 | Hemato (Lymphoma) | >5 yr | NR | Fatigue Questionnaire (FQ) | Cont | Single question on employment | Cat (Unemployment/Non-RTW) | OR | 3.61 | 95% CI: 1.67 to 7.84 | FQ: higher scores indicate more fatigue. |
| Kiserud 2016 | Hemato (Lymphoma) | Mean = 12.4 yr | Mean = 9.7 yr | Fatigue Questionnaire (FQ) | Cont | Single question on employment | Cat (Unemployment/Non-RTW) | OR | 0.97 | 95% CI: 0.9 to 1.06 | FQ: higher scores indicate more fatigue. |
| Lee 2017 | Breast | Up to 36 m | NR | Brief Fatigue Inventory (BFI) | Cont | Single question on employment at baseline, 12m, 24m, 36m post dx | Cat (Employment/RTW) | OR | - | - | Not included in final model based on univariate analysis, no estimates reported. |
| Lee 2017 | Breast | Up to 36 m | NR | EORTC-QLQ-Core 30 subscale | Cont | Single question on employment at baseline, 12m, 24m, 36m post dx | Cat (Employment/RTW) | OR | - | - | Symptom burden assessed at 12 and 24 months. Not included in final model based on univariate analysis, no estimates reported. |
| Lindbohm 2014 | Breast | 1 to 8 yr | NR | Single question on symptom (Never, seldom, often, all the time) | Cat (Never/seldom, often/all the time) | Single question on employment | Cat (Employed, early retirement, other non-employment) | OR | Retire: 0.90; Other: 1.97 | 95% CI: Retire: 0.45 to 1.79 Other: 1.03 to 3.78 |  |
| Murray 2019 | Various (Testicular 19%; Hemato 17%) | 18 m | NR | Clinical diagnosis | Cat | Institution-specified criteria to lift work restrictions | Time to event | HR | 0.76 | 95% CI: 0.35 to 1.65 | RTW defined as lifting of work restrictions based on physician assessment. Symptom burden assessed at 6 months. |
| Murray 2019 | Various (Testicular 19%; Hemato 17%) | 18 m | NR | Clinical diagnosis | Cat | Institution-specified criteria to lift work restrictions | Cat (Employment/RTW) | OR | 0.16 | 95% CI: 0.05 to 0.56 | RTW defined as lifting of work restrictions based on physician assessment. Symptom burden assessed at 6 months. |
| Nieuwenhuijsen 2009 | Various (GI tract 31%; breast 20%) | [12 m after first day of sick leave] | NR | Multidimensional Fatigue Inventory (MFI) | Cont | Single question on employment | Cat (Employment/RTW) | OR | - | - | No estimates reported but not statistically signficant. |
| Pryce 2007 | Various (Breast 48%) | NR | NR | Single question on symptom | Cat | Single question on employment | Cat (Employment/RTW) | OR | 1.49 | 95% CI: 1.42 to 6.18 |  |
| Schmidt 2019 | Breast | NR | [>1 yr from surgery] Median = 247 days | Fatigue Assessment Questionnaire (FAQ) subscale | Cat (Higher than median age norm values) | Several questions on working status and hours | Cat (Reduced hours or discontinuation of work) | OR | 0.7 | 95% CI: 0.2 to 2.5 | Symptom burden assessed at week 13 post surgery. |
| Schmidt 2019 | Breast | NR | [>1 yr from surgery] Median = 247 days | Fatigue Assessment Questionnaire (FAQ) subscale | Cat (Higher than median age norm values) | Several questions on working status and hours | Cat (Reduced hours or discontinuation of work) | OR | 6.3 | 95% CI: 1.7 to 23.4 | Symptom burden assessed at month 15 post surgery. |
| Spelten 2003 | Various (Gynae 25%; breast 24%) | [18 m after sick leave] | NR | Multidimensional Fatigue Inventory (MFI) subscale | Not specified | Single question on employment | Time to event | HR | - | - | Time to RTW measured from first day of sick leave. Symptom burden measured at 6 months after sick leave. Not included in final model due to multicollinearity. Univariate estimates = 0.71 (0.59-0.85). |
| Steiner 2008 | Various (Breast 20%; GI tract 13%) | Mean = 23 m | NR | Supportive Care Needs Survey (Bonevski 2000) item | Cat | Adapted from Health and Retirement Survey | Cat | Proportion | - | - | No estimates reported, assumed to be p > 0.05. |
| Tamminga 2016 | Thyroid | Mean = 9.9 yr | NR | Fatigue Assessment Scale (FAS) | Cont | Single question on employment | Cat (Unemployment/Non-RTW) | OR | 1.07 | 95% CI: 1.01 to 1.14 | FAS: higher scores indicate more fatigue. |
| Tevaarwerk 2013 | Various (Breast 74%) | >6 m (6 to 12 m: 13%; 12 to 24 m: 24%; >24 m: 63%) | NR | MD Anderson Symptom Inventory-Modified (MDASI-ECOG) | Cat (<4, >=5) | Two questions on current employment status and if changes occurred due to illness | Cat (Unemployment/Non-RTW) | OR | 2.3 | 95% CI: 1.1 to 4.7 | MDASI: threshold based on MDASI recommendations with >=5 indicating severe level. |
| Verdonck-de Leeuw 2010 | H&N | NR | >2 yr | EORTC-QLQ-Core 30 subscale | Cont | Single question on employment change | Cat (Return to same work, changed work, no RTW) | Mean scores | 21.42 (same) vs 26.39 (changed) vs 41.67 (no RTW) | SD = 20.49 (same) vs 21.42 (changed) vs 38.38 (no RTW) | EORTC: higher scores indicate higher level of sx burden. |
| Yoo 2013 | Gynae (Cervical) | NR | <5 yr: 29%; >=5 yr: 71% | EORTC-QLQ-Core 30 subscale | Cont | Single question on employment | Cat | Mean scores | 43.2 (emp) vs 45.39 (unemp) | - | GLM used to control for demographic and clinical covariates. EORTC: higher scores indicate higher level of symptom burden. |
| Zomkowski 2020 | Breast | [Mean time from surgery = 48 m] | >1 m | EORTC-QLQ-Core 30 single item | Cont | Single question on employment | Cat | Mean scores | 25.51 (RTW) vs 38.41 (no RTW) | SD = 25.56 (RTW) vs 32.6 (no RTW) | p = 0.048. EORTC: higher scores indicate higher level of sx burden. |
| **Work status (Early retirement/disability)** | | | | | | | | | | | |
| Dahl 2020 | Gynae (Cervical) | Mean = 11 yr | NR | Fatigue Questionnaire (FQ) | Cont | Single question on disability | Cat (Disability) | OR | 1.09 | 95% CI: 0.48 to 2.47 | Disability defined as being on disability pension. FQ: higher scores indicate more fatigue. |
| Van Muijen 2014, 2017, 2019 | Various (Breast 40%) | >24 m | NR | Functional Assessment of Chronic Illness-Fatigue scale (FACIT-F) | Cat (>27) | Institution-specified criteria | Cat (Disability) | OR | - | - | Work disability defined as >80% wage loss. Not included in final model based on stepwise backward selection, no estimates reported. FACIT-F: threshold of 27 used but unjustified. |
| **Work ability** | | | | | | | | | | | |
| Dahl 2019 | Various (Breast 41%) | >6 yr (Mean = 17 yr) | NR | Fatigue Questionnaire (FQ) | Cont | Work Ability Index (WAI) - 1st qns only | Cat (Low work ability, ie. <=7) | OR | 1.13 | 95% CI: 1.08 to 1.18 | FQ: higher scores indicate more fatigue.; WAI: threshold of 7 previously validated. |
| Dorland 2018 | Various (Breast 46%) | 6, 12 and 18 m | NR | Checklist for Individual Strength (CIS) subscale | Cont | Work Role Functioning Questionnaire (WRFQ) | Cont | Beta | -0.16 | 95% CI:  -0.08 to  -0.16 | GEE used to control for time since RTW. CIS: higher scores indicate more fatigue; WRFQ: higher scores indicate better ability. |
| Ho 2018 | Breast | >1 yr (Median = 4 yr) | NR | Multidimensional Fatigue Inventory (MFI) subscale | Cont | Work Ability Index (WAI) | Cont | Std beta | -1.7 | 95% CI:  -2.5 to -0.9 | MFI: higher scores indicate more fatigue; WAI: higher scores indicate greater ability. Subscales on mental fatigue, reduced activity and reduced motivation were not included in SR summary. |
| Nieuwenhuijsen 2009 | Various (GI tract 31%; breast 20%) | [12 m after first day of sick leave] | NR | Multidimensional Fatigue Inventory (MFI) | Cont | Work Ability Index (WAI) - 1st qns only | Cont | Beta | - | - | No estimates reported but not statistically signficant. |
| Van Muijen 2014, 2017, 2019 | Various (Breast 46%) | [36 m after sick leave] | NR | Functional Assessment of Chronic Illness-Fatigue scale (FACIT-F) | Cont | Work Ability Index (WAI) - 1st qns only | Cont | Std beta | - | - | Not included in final model based on stepwise forward selection, no estimates reported. |
| Van Muijen 2014, 2017, 2019 | Various (Breast 47%) | >24 m | NR | Functional Assessment of Chronic Illness-Fatigue scale (FACIT-F) | Cont | Work Ability Index (WAI) - 1st qns only | Cont | Std beta | 0.52 | - | FACIT-F: higher scores indicate lower level of fatigue; WAI: higher scores indicate greater ability. |
| Wolvers 2019 | Various (Breast 84%) | 12 m | NR | Multidimensional Fatigue Inventory (MFI) | Cont (Change from last assessment) | Work Ability Index (WAI) - 1st qns only | Cont (Change from last assessment) | Beta | -0.19 | - | p < 0.001, SE not reported. MFI: higher scores indicate higher level of sx burden; WAI: higher scores indicate greater working ability. Part of interventional trial cohort. |
| Wolvers 2019 | Various (Breast 84%) | 18 m | NR | Multidimensional Fatigue Inventory (MFI) | Cont (Change from last assessment) | Work Ability Index (WAI) - 1st qns only | Cont (Change from last assessment) | Beta | -0.176 | - | p = 0.046, SE not reported. MFI: higher scores indicate higher level of sx burden; WAI: higher scores indicate greater working ability. Part of interventional trial cohort. |
| **Work productivity** | | | | | | | | | | | |
| Brick 2019 | Breast | NR | [6 m after surgery and start of chemotherapy or endocrine therapy] | Epworth Sleepiness Scale | Cont | Work Limitations Questionnaire (WLQ) | Cont | Correlation | 0.405 | - | p = 0.055. Epsworth Sleepiness Scale: higher scores indicated higher level of sx; WLQ: higher scores indicate lower productivity. |
| Calvio 2010 | Breast | NR | 1 to 10 yr (Mean = 3 yr) | Multidimensional Fatigue Symptom Inventory (MFSI) | Cont | Work Limitations Questionnaire (WLQ) subscale | Cont | Beta | - | - | Productivity defined as work output measured using WLQ. Not included in final model based on multistep univariate analysis, no estimates for final model reported. Univariate analysis with demographic confounders = 0.11. MFSI: higher scores indicate higher level of sx burden; WLQ: higher scores indicate lower output. Multiple measures of fatigue will lead to multicollinearity. |
| Calvio 2010 | Breast | NR | 1 to 10 yr (Mean = 3 yr) | VAS of 10cm | Cont | Work Limitations Questionnaire (WLQ) subscale | Cont | Beta | - | - | Productivity defined as work output measured using WLQ. Not included in final model based on multistep univariate analysis, no estimates for final model reported. Univariate analysis with demographic confounders = 0.18. WLQ: higher scores indicate lower output. Multiple measures of fatigue will lead to multicollinearity. |
| Hansen 2008 | Breast | Mean = 3.8 yr | NR | Multidimensional Fatigue Symptom Inventory-Short Form (MFSI-SF) subscale | Cont | Work Limitations Questionnaire (WLQ) | Cont | Beta | 0.448 | 95% CI: 0.299 to 0.597 | MFSI-SF: higher scores indicate more fatigue; WLQ: higher scores indicate lower productivity. Patients with chronic comorbid diseases were excluded but no specification of which diseases. |
| Lavigne 2008 | Breast | NR | >12 m (Mean = 36 m) | SF-36 vitality scale | Cat (<1 SD below age-sex norm) | Work Limitations Questionnaire (WLQ) | Cont | Beta | 1.55 | 95% CI: 0.02 to 3.08 | SF-36 threshold based on previous reported methods. WLQ: higher scores indicate lower productivity. |
| **Absenteeism** | | | | | | | | | | | |
| Lavigne 2008 | Breast | NR | >12 m (Mean = 36 m) | SF-36 vitality scale | Cat (<1 SD below age-sex norm) | Work Limitations Questionnaire (WLQ) | Cont | Beta | 1.55 | - | SF-36 threshold based on previous reported methods. WLQ: higher scores indicate lower productivity. |
| **Others (Changes in work)** | | | | | | | | | | | |
| Steiner 2008 | Various (Breast 20%; GI tract 13%) | Mean = 23 m | NR | Supportive Care Needs Survey (Bonevski 2000) item | Cat | Adapted from Health and Retirement Survey | Cat | Proportion | 94% (With changes in work) vs 68% (None) | - | Change in work defined as changes in work characteristics or reduction in work hours. p < 0.001. |
| **Others (Occupational role limitations)** | | | | | | | | | | | |
| Steiner 2008 | Various (Breast 20%; GI tract 13%) | Mean = 23 m | NR | Supportive Care Needs Survey (Bonevski 2000) item | Cat | Occupational Role Questionnaire items | Cat | Proportion | 98% (With limitations) vs 63% (None) | - | p < 0.001. |

**Fatigue/sleep-related: Insomnia**

| Study | Cancer | Time since dx | Time since tx | Symptom | | Work outcome | | Effect measure | Estimate | Spread | Notes |
| --- | --- | --- | --- | --- | --- | --- | --- | --- | --- | --- | --- |
|  |  |  |  | Measurement tool | Variable | Measurement tool | Variable |  |  |  |  |
| **Work status (Employment/RTW)** | | | | | | | | | | | |
| Agarwal 2017 | H&N | NR | >6 m (Mean = 19 m) | EORTC-QLQ-Core 30 single item | Cont | Single question on employment | Cat | Mean scores | 11.3 (RTW) vs 12.7 (non-RTW) | SD = 23.6 (RTW) vs 25.6 (non-RTW) | p = 0.864. EORTC: higher scores indicate higher level of sx burden. |
| Check 2019 | H&N (Oropharyngeal squamous cell carcinoma) | 1 to 22 yr (Median = 6 yr) | >1 yr | MD Anderson Symptom Inventory-Head and Neck (MDASI-HN) module | Cont | Single question on employment | Cat | Mean scores | 1.6 (emp) vs 2 (unemp) | SD = 2.2 (emp) vs 2.7 (unemp) | p = 0.18. MDASI: higher scores indicate higher level of sx burden. |
| Cooper 2013 | Various (Urology: 32%; breast: 31%) | [Median time since start of sick leave = 4.7 m] | [Median time since start of tx = 3.9 m] | EORTC-QLQ-Core 30 single item | Cont | Single question on employment at baseline, 6m and 12m | Time to event | HR | - | - | Time to RTW measured from cessaton of work. Sx covariate not included in final model based on stepwise forward selection if not stated. |
| Duijts 2017 | Various (Breast 48%) | 2 to 4 yr | NR | EORTC-QLQ-Core 30 single item | Cont | Single question on employment at 2, 3 and 4 years after dx | Cat (Sustained unemployment, positive change, negative change, sustained employment) | Mean scores | 43.1 (sust unemp) vs 34.8 (+ change) vs 35.5 (- change) vs 30.4 (sust emp) | - | p = 0.068. EORTC: higher scores indicate higher level of sx burden. |
| Duijts 2017 | Various (Breast 48%) | 2 to 4 yr | NR | EORTC-QLQ-Core 30 single item | Cont | Single question on employment at 2, 3 and 4 years after dx | Cat (Sustained employment) | OR | - | - | Time lag GEE model used to correlate work continuation one year later with symptom burden. Not included in final model based on univariate analysis, no estimates reported. |
| Kim 2014 | Lung | Median = 4.1 yr | NR | EORTC-QLQ-Core 30 single item | Cat (>66) | Single question on employment | Cat (Employment/RTW) | OR | - | - | Not included in final model based on stepwise selection. Univariate estimates = 1.66 (1.03-2.68). EORTC: threshold based on previous studies with >66 indicating problematic status. Demographic statistics based on full cohort, which includes subjects not analysed for RTW. |
| Lee 2017 | Breast | Up to 36 m | NR | EORTC-QLQ-Core 30 single item | Cont | Single question on employment at baseline, 12m, 24m, 36m post dx | Cat (Employment/RTW) | OR | - | - | Symptom burden assessed at 12 and 24 months. Not included in final model based on univariate analysis, no estimates reported. |
| Spelten 2003 | Various (Gynae 25%; breast 24%) | [18 m after sick leave] | NR | Pittsburgh Sleep Quality Index | Not specified | Single question on employment | Time to event | HR | - | - | Time to RTW measured from first day of sick leave. Symptom burden measured at 6 months after sick leave. Not included in final model based on univariate analysis and stepwise forward selection, no estimates reported. |
| Steiner 2008 | Various (Breast 20%; GI tract 13%) | Mean = 23 m | NR | Supportive Care Needs Survey (Bonevski 2000) item | Cat | Adapted from Health and Retirement Survey | Cat | Proportion | - | - | No estimates reported, assumed to be p > 0.05. |
| Tevaarwerk 2013 | Various (Breast 74%) | >6 m (6 to 12 m: 13%; 12 to 24 m: 24%; >24 m: 63%) | NR | MD Anderson Symptom Inventory-Modified (MDASI-ECOG) | Cat (<4, >=5) | Two questions on current employment status and if changes occurred due to illness | Cat (Unemployment/Non-RTW) | OR | - | - | Not included in final model based on stepwise forward selection, no estimates for final model reported. Univariate estimates = 4.6 (3.0-7.2). MDASI: threshold based on recommendations with >=5 indicating severe level. |
| Verdonck-de Leeuw 2010 | H&N | NR | >2 yr | EORTC-QLQ-Core 30 single item | Cont | Single question on employment change | Cat (Return to same work, changed work, no RTW) | Mean scores | 16.67 (same) vs 33.33 (changed) vs 33.33 (no RTW) | SD = 21.27 (same) vs 34.42 (changed) vs 30.86 (no RTW) | EORTC: higher scores indicate higher level of sx burden. |
| Yoo 2013 | Gynae (Cervical) | NR | <5 yr: 29%; >=5 yr: 71% | EORTC-QLQ-Core 30 single item | Cont | Single question on employment | Cat | Mean scores | 26.04 (emp) vs 25.84 (unemp) | - | GLM used to control for demographic and clinical covariates. EORTC: higher scores indicate higher level of symptom burden. |
| **Work status (Early retirement/disability)** | | | | | | | | | | | |
| Dahl 2020 | Gynae (Cervical) | Mean = 11 yr | NR | EORTC-QLQ-Core 30 single item | Cont | Single question on disability | Cat (Disability) | OR | 1 | 95% CI: 0.99 to 1.01 | Disability defined as being on disability pension. EORTC: higher scores indicate higher level of sx burden. |
| **Work productivity** | | | | | | | | | | | |
| Hansen 2008 | Breast | Mean = 3.8 yr | NR | Single question on sufficient sleep | Cat | Work Limitations Questionnaire (WLQ) | Cont | Beta | -0.464 | 95% CI: -1.5 to 0.6 | WLQ: higher scores indicate lower productivity. Patients with chronic comorbid diseases were excluded but no specification of which diseases. |
| **Others (Changes in work)** | | | | | | | | | | | |
| Steiner 2008 | Various (Breast 20%; GI tract 13%) | Mean = 23 m | NR | Supportive Care Needs Survey (Bonevski 2000) item | Cat | Adapted from Health and Retirement Survey | Cat | Proportion | 65% (With changes in work) vs 55% (None) | - | Change in work defined as changes in work characteristics or reduction in work hours. p = 0.31. |
| **Others (Occupational role limitations)** | | | | | | | | | | | |
| Steiner 2008 | Various (Breast 20%; GI tract 13%) | Mean = 23 m | NR | Supportive Care Needs Survey (Bonevski 2000) item | Cat | Occupational Role Questionnaire items | Cat | Proportion | 67% (With limitations) vs 53% (None) | - | p = 0.17. |

**Fatigue/sleep-related: Drowsiness**

| Study | Cancer | Time since dx | Time since tx | Symptom | | Work outcome | | Effect measure | Estimate | Spread | Notes |
| --- | --- | --- | --- | --- | --- | --- | --- | --- | --- | --- | --- |
|  |  |  |  | Measurement tool | Variable | Measurement tool | Variable |  |  |  |  |
| **Work status (Employment/RTW)** | | | | | | | | | | | |
| Check 2019 | H&N (Oropharyngeal squamous cell carcinoma) | 1 to 22 yr (Median = 6 yr) | >1 yr | MD Anderson Symptom Inventory-Head and Neck (MDASI-HN) module | Cont | Single question on employment | Cat | Mean scores | 1.4 (emp) vs 1.8 (unemp) | SD = 2 (emp) vs 2.3 (unemp) | p = 0.01. MDASI: higher scores indicate higher level of sx burden. |
| Tevaarwerk 2013 | Various (Breast 74%) | >6 m (6 to 12 m: 13%; 12 to 24 m: 24%; >24 m: 63%) | NR | MD Anderson Symptom Inventory-Modified (MDASI-ECOG) | Cat (<4, >=5) | Two questions on current employment status and if changes occurred due to illness | Cat (Unemployment/Non-RTW) | OR | - | - | Not included in final model based on univariate analysis and stepwise forward selection, no estimates reported as non-significant in univariate analysis. MDASI: threshold based on MDASI recommendations with >=5 indicating severe level. |

**Others: Hot flashes**

| Study | Cancer | Time since dx | Time since tx | Symptom | | Work outcome | | Effect measure | Estimate | Spread | Notes |
| --- | --- | --- | --- | --- | --- | --- | --- | --- | --- | --- | --- |
|  |  |  |  | Measurement tool | Variable | Measurement tool | Variable |  |  |  |  |
| **Work productivity** | | | | | | | | | | | |
| Lavigne 2008 | Breast | NR | >12 m (Mean = 36 m) | Not specified | Cat | Single question on absenteeism | Cont | Beta | - | - | Not included in final model based on backward selection, no estimates reported. |
| **Absenteeism** | | | | | | | | | | | |
| Lavigne 2008 | Breast | NR | >12 m (Mean = 36 m) | Not specified | Cat | Work Limitations Questionnaire (WLQ) | Cont | Beta | 2.18 | 95% CI: 1.1 to 3.26 | WLQ: higher scores indicate lower productivity. |

**Others: Rash**

| Study | Cancer | Time since dx | Time since tx | Symptom | | Work outcome | | Effect measure | Estimate | Spread | Notes |
| --- | --- | --- | --- | --- | --- | --- | --- | --- | --- | --- | --- |
|  |  |  |  | Measurement tool | Variable | Measurement tool | Variable |  |  |  |  |
| **Work status (Employment/RTW)** | | | | | | | | | | | |
| Check 2019 | H&N (Oropharyngeal squamous cell carcinoma) | 1 to 22 yr (Median = 6 yr) | >1 yr | MD Anderson Symptom Inventory-Head and Neck (MDASI-HN) module | Cont | Single question on employment | Cat | Mean scores | 0.3 (emp) vs 0.4 (unemp) | SD = 1 (emp) vs 1.3 (unemp) | p = 0.01. MDASI: higher scores indicate higher level of sx burden. |
| Tevaarwerk 2013 | Various (Breast 74%) | >6 m (6 to 12 m: 13%; 12 to 24 m: 24%; >24 m: 63%) | NR | MD Anderson Symptom Inventory-Modified (MDASI-ECOG) | Cat (<4, >=5) | Two questions on current employment status and if changes occurred due to illness | Cat (Unemployment/Non-RTW) | OR | - | - | Not included in final model based on univariate analysis and stepwise forward selection, no estimates reported as non-significant in univariate analysis. MDASI: threshold based on recommendations with >=5 indicating severe level. |

**Others: Sexual dysfunction**

| Study | Cancer | Time since dx | Time since tx | Symptom | | Work outcome | | Effect measure | Estimate | Spread | Notes |
| --- | --- | --- | --- | --- | --- | --- | --- | --- | --- | --- | --- |
|  |  |  |  | Measurement tool | Variable | Measurement tool | Variable |  |  |  |  |
| **Work status (Employment/RTW)** | | | | | | | | | | | |
| Lee 2017 | Breast | Up to 36 m | NR | EORTC-QLQ-Breast 23 subscale | Cont | Single question on employment at baseline, 12m, 24m, 36m post dx | Cat (Employment/RTW) | OR | - | - | Symptom burden assessed at 12 and 24 months. Not included in final model based on univariate analysis, no estimates reported. |
| Zomkowski 2020 | Breast | [Mean time from surgery = 48 m] | >1 m | EORTC-QLQ-Breast 23 subscale | Cont | Single question on employment | Cat | Mean scores | 62.96 (RTW) vs 72.38 (no RTW) | SD = 29.72 (RTW) vs 25.23 (no RTW) | p = 0.091. EORTC: higher scores indicate lower level of sx burden. |

**Others: Urinary problems**

| Study | Cancer | Time since dx | Time since tx | Symptom | | Work outcome | | Effect measure | Estimate | Spread | Notes |
| --- | --- | --- | --- | --- | --- | --- | --- | --- | --- | --- | --- |
|  |  |  |  | Measurement tool | Variable | Measurement tool | Variable |  |  |  |  |
| **Work status (Employment/RTW)** | | | | | | | | | | | |
| Bennett 2018 | Prostate | 18 to 42 m | NR | Expanded Prostate Cancer Index Composite (EPIC) item | Cat (No/ small, moderate/  big) | Single question on employment change | Cat (Employment loss) | OR | 2.02 | 95% CI: 1.37 to 2.97 | Not included in final model based on univariate analysis, no estimates reported. |
| Dahl 2015, 2016 | Prostate | NR | >3 yr | Expanded Prostate Cancer Index Composite (EPIC) item | Cat (No/ small, moderate/  big) | Single question on employment | Cat (Diminished employment) | OR | - | - | Not included in final model based on univariate analysis, no estimates for final model reported. Univariate estimates = 1.50 (0.62-3.65). |
| Nakamura 2017 | Gynae (Cervical) | NR | >1 yr (Mean = 4.5 yr) | National Cancer Institute Common Toxicity Criteria (CTC) | Cat (Present, ie stage II and above) | Single question on employment | Cat (Non RTW) | Proportion | 1.4% (Fail) vs 8.7% (RTW) | - | Demographics based on full cohort, which includes subjects not analysed for job change. Failure to RTW + RTW inconsistent with total N analysed. |
| **Work ability** | | | | | | | | | | | |
| Dahl 2015, 2016 | Prostate | NR | Mean = 3 yr | Expanded Prostate Cancer Index Composite (EPIC) item | Cat (>= 1 pad, no pad use) | Work Ability Index (WAI) - 1st qns only | Cat (Excellent, good, poor/moderate) | OR | Good: 1.57; poor/moderate: 2.32 | 95% CI: Good: 0.98 to 2.51 Poor/moderate: 1.36 to 3.97 | WAI: thresholds used based on questionnaire developer. |
